# Intrahost cytomegalovirus population genetics following antibody pretreatment in a monkey model of congenital transmission

**DOI:** 10.1371/journal.ppat.1007968

**Published:** 2020-02-14

**Authors:** Diana Vera Cruz, Cody S. Nelson, Dollnovan Tran, Peter A. Barry, Amitinder Kaur, Katia Koelle, Sallie R. Permar

**Affiliations:** 1 Computational Biology and Bioinformatics program / Duke Center for Genomic and Computational Biology, Duke University, Durham, North Carolina, United States of America; 2 Human Vaccine Institute, Duke University School of Medicine, Durham, North Carolina, United States of America; 3 Tulane National Primate Research Center, Tulane University, Covington, Louisiana, United States of America; 4 Center for Comparative Medicine, Department of Pathology and Laboratory Medicine, University of California, Davis, California, United States of America; 5 Department of Biology, Emory University, Atlanta, Georgia, United States of America; University of Wisconsin-Madison, UNITED STATES

## Abstract

Human cytomegalovirus (HCMV) infection is the leading non-genetic cause of congenital birth defects worldwide. While several studies have addressed the genetic composition of viral populations in newborns diagnosed with HCMV, little is known regarding mother-to-child viral transmission dynamics and how therapeutic interventions may impact within-host viral populations. Here, we investigate how preexisting CMV-specific antibodies shape the maternal viral population and intrauterine virus transmission. Specifically, we characterize the genetic composition of CMV populations in a monkey model of congenital CMV infection to examine the effects of passively-infused hyperimmune globulin (HIG) on viral population genetics in both maternal and fetal compartments. In this study, 11 seronegative, pregnant monkeys were challenged with rhesus CMV (RhCMV), including a group pretreated with a standard potency HIG preparation (*n* = 3), a group pretreated with a high-neutralizing potency HIG preparation (*n* = 3), and an untreated control group (*n* = 5). Targeted amplicon deep sequencing of RhCMV glycoprotein *B* and *L* genes revealed that one of the three strains present in the viral inoculum (UCD52) dominated maternal and fetal viral populations. We identified minor haplotypes of this strain and characterized their dynamics. Many of the identified haplotypes were consistently detected at multiple timepoints within sampled maternal tissues, as well as across tissue compartments, indicating haplotype persistence over time and transmission between maternal compartments. However, haplotype numbers and diversity levels were not appreciably different between control, standard-potency, and high-potency pretreatment groups. We found that while the presence of maternal antibodies reduced viral load and congenital infection, it had no apparent impact on intrahost viral genetic diversity at the investigated loci. Interestingly, some minor haplotypes present in fetal and maternal-fetal interface tissues were also identified as minor haplotypes in corresponding maternal tissues, providing evidence for a loose RhCMV mother-to-fetus transmission bottleneck even in the presence of preexisting antibodies.

## Author summary

Human cytomegalovirus (CMV) is the most common infectious cause of birth defects worldwide. Knowledge gaps remain regarding how maternal immunity impacts the genetic composition of CMV populations and the incidence of congenital virus transmission. Addressing these gaps is important to inform vaccine development efforts. Using viral DNA isolated from a monkey model of congenital CMV infection, we investigated the impact of passively-administered maternal antibodies on the genetic composition of the maternal virus population and that transmitted to the fetus. Our analysis focused on regions of two CMV genes that encode glycoproteins known to facilitate viral cellular entry and to be targeted by CMV-specific antibodies. We investigated viral haplotypes across sampled maternal tissues and amniotic fluid, finding no impact of preexisting CMV-specific antibodies on CMV genetic diversity despite the observation that antibodies reduce viral load and can confer protection against congenital transmission. We further found that some minor haplotypes identified in fetal and maternal-fetal interface tissues were also present in paired maternal tissues. This finding indicates that a large number of viral particles likely passed from dam to fetus during congenital transmission.

## Introduction

Human cytomegalovirus (HCMV) is a member of the β-herpesvirus family and a ubiquitous pathogen that establishes lifelong infection in its host. Seroprevalence rates for HCMV range from 45% in developed nations to 100% in developing nations [1]. While initial HCMV infection is typically asymptomatic in the setting of intact host immunity, congenitally infected infants, immune-compromised individuals, and transplant recipients can suffer adverse HCMV-related outcomes [2–4]. Indeed, HCMV impacts approximately 1 in 150 live-born infants worldwide, making this virus the leading infectious cause of congenital birth defects. Among infants infected at birth, 10–20% will develop long-term sequelae including sensorineural hearing loss, microcephaly, and cognitive impairment [2].

Congenital CMV infection during pregnancy can result from either primary infection or viral reactivation and/or superinfection (secondary infection). While congenital infection could be seeded from the maternal genital tract [5,6], most cases of transmission are thought to occur transplacentally [7,8]. High levels of maternal HCMV viremia and maternal infection earlier during gestation have been correlated with a greater risk of congenital infection and more severe congenital disease [9,10]. Following congenital infection, HCMV can be disseminated throughout the developing fetus with HCMV detectable in multiple fetal tissues in almost 50% of cases [11].

Recent whole-genome sequencing has revealed that, despite being a DNA virus, HCMV exhibits substantial population-level genetic diversity [12–15]. Across hosts, levels of HCMV genetic diversity can be quite variable: some studies have found infected individuals to harbor low levels of HCMV genetic diversity [16–18] while others have found infected individuals to harbor extensive HCMV diversity [13,16,17,19,20]. Mixed infections and strain recombination appear to play key roles in the generation of HCMV diversity in those individuals with high viral diversity, including congenitally infected neonates, immunocompromised children, and organ transplant recipients [15,17]. *De novo* point mutations are also thought to play a contributing role in the generation of observed intrahost genetic diversity [18,19,21]. The occurrence of these mutations has been highlighted in studies that have characterized low-frequency intrahost HCMV variants in congenital CMV cases [19]. Other studies, in contrast, have not found strong evidence for *de novo* mutations arising in intrahost CMV populations [16]. These studies and others [16–18], however, have consistently shown that dynamic (and at times, dramatic) changes in the genetic composition of CMV populations over the course of infection are possible. This is not always the case, however, as the genetic composition of HCMV populations appear to remain stable in some infected individuals [18]. Furthermore, differences in the genetic composition of HCMV populations between tissues have also been identified [13,19,22]. Such differences may be due to tissue-specific adaptations, as viral genomes obtained from the same anatomical compartment across different hosts have been found to exhibit characteristic genetic similarities [13].

One of the challenges of HCMV research is that herpesviruses are highly species-specific [23], which has led to a reliance on human clinical trials [7]. Yet, congenital virus transmission can be modeled using both guinea pigs and nonhuman primate models [7,24]. In particular, rhesus macaques and their corresponding endogenous rhesus CMV (RhCMV) are a highly-relevant model for understanding adult/fetal HCMV pathogenesis [25,26] and congenital infection [9,27], as the physiology/immunology of rhesus monkey pregnancy is highly analogous to humans [25], there is extensive protein homology between RhCMV and HCMV [28], and certain mechanisms of viral immune evasion are conserved between these viruses [29,30]. Previously, our group demonstrated that the depletion of CD4^+^ T cells followed by intravenous RhCMV inoculation of seronegative pregnant monkeys resulted in consistent RhCMV congenital infection and a high rate of fetal loss [24]. We subsequently tested the impact of preexisting antibodies on the incidence and severity of congenital CMV transmission in this monkey model via passive infusion of hyperimmune globulin (HIG) prior to RhCMV inoculation. This study established that preexisting RhCMV-specific antibodies (“standard-potency” HIG) can prevent fetal loss in the absence of functional CD4+ T cell immunity and that highly-neutralizing antibodies (“high-potency” HIG) may block congenital transmission altogether [9]. Furthermore, this previous work demonstrated that potently-neutralizing antibodies present at the time of primary infection can alter viral dynamics *in vivo* [9].

In this investigation, we focus on understanding the dynamics of transplacental transmission as well as on the impact of HIG pretreatment on the genetic composition of RhCMV populations found across maternal and fetal tissue compartments. Our analysis is based on RhCMV sequence data derived from maternal compartment samples (plasma, saliva, and urine), samples from the maternal-fetal interface (amniotic fluid and placenta), and fetal tissue samples (fetal heart, brain, lungs, kidney and spleen), where available. Due to the large genome size of RhCMV and a desire to identify viral haplotypes, we focused our approach on amplicon sequencing of variable regions of antibody-targeted glycoprotein genes *gB* and *gL* to explore the effects of preexisting antibodies on viral evolution and tissue compartmentalization.

We hypothesized that HIG pretreatment might have one of two possible effects on RhCMV diversity at the sequenced *gB* and *gL* gene regions. One possibility is that HIG pretreatment could increase the strength of purifying selection, and thus decrease observed levels of genetic diversity at these loci. Indeed, previous studies have highlighted the prominent role that purifying selection plays in intra- and inter-host CMV evolution [12,19]. Given our previous finding that HIG pretreatment of dams reduces peak plasma viral load and the risk of congenital transmission, we may expect HIG pretreatment to strengthen *in vivo* selection pressures and thus increase the strength of purifying selection. A second, distinct possibility is that HIG pretreatment could place RhCMV populations under immune selection, and thus increase observed levels of *gB* and *gL* genetic diversity. Indeed, a previous study has found evidence for positive selection at CMV loci under immune selection [19] and other studies have found evidence for diversifying selection in genes encoding envelope glycoproteins that are targeted by the immune response [12–14]. Surprisingly, our population genetic analyses at these loci indicate no systematic differences in genetic diversity or the number of minor CMV haplotypes across maternal tissues in dams from pretreated versus control groups. However, we did find low-frequency (minor) haplotypes that persisted over time within and between maternal compartments in each of the groups studied. We further found multiple low-frequency haplotypes that were shared between maternal tissues and fetal/maternal-fetal interface tissues, indicating the presence of a relatively loose transmission bottleneck between mother and fetus. These findings contribute to the deeper understanding of maternal and congenital infection dynamics that might inform the development of therapeutic interventions to prevent congenital CMV infection.

## Methods

### Study setting

The primary focus of the study analyzed here was to investigate the ability of preexisting maternal antibodies to inhibit congenital CMV transmission. The study consisted of three groups of monkeys: a control group that received no hyperimmune globulin (HIG) pretreatment (*n* = 5, 3 of which were historical controls [27]), a “standard” pretreatment group (*n* = 3), and a “high-potency” pretreatment group (*n* = 3). All eleven pregnant RhCMV-seronegative dam monkeys were first depleted of CD4^+^ T cells using an anti-CD4 monoclonal antibody, and one week later intravenously inoculated with RhCMV, as previously described [9]. The three dams in the standard HIG pretreatment group each received a single dose of a standard HIG preparation 1 hour prior to viral inoculation. The three dams in the high-potency pretreatment group each received an initial dose 1 hour prior to viral inoculation and a second dose 3 days later. Both doses in the high-potency group used a high-potency HIG preparation by screening serum donor monkeys for serum RhCMV neutralizing activity, as described in [9].

The RhCMV inoculum was a mixture of three different strains: UCD52, UCD59, and 180.92, at relative frequencies of 25%, 25%, and 50% (by infectious viral titer), respectively. Both UCD52 and UCD59 are derived from serial propagation on primary monkey kidney epithelial cells, which express epithelial cell morphology and cell-specific markers [31] and contain a full-length UL128-UL131 coding capacity [32]. Previous studies have shown that inoculation of RhCMV-naïve rhesus macaques with UCD52 and UCD59 results in similar patterns of viremia and shedding in bodily fluids, comparable to those observed in colony-reared macaques naturally infected with circulating strains of endemic RhCMV [32–34]. On the other hand, CMV strain 180.92 was derived from serial propagation on rhesus fibroblasts, and is known to be a mixed virus with the dominant strain containing deletions in the UL/b’ region while retaining an intact UL128-UL131 locus. Experimental inoculation of rhesus macaques with 180.92 showed rapid emergence of a minor wild-type like variant but only limited tissue dissemination and viral excretion of the defective UL/b' virus strain [35].

For each of the 11 dams studied, samples were taken from maternal blood plasma, urine, saliva, and amniotic fluid at multiple time points following infection. Sample availability varied across dams for reasons such as early fetal loss or low sample volume, previously described in [9]. A subset of the available samples had virus populations that were successfully sequenced and form the basis of our analysis **(S1 Table)**. The remainder of these samples did not have successful viral sequencing due to either low viral loads or inadequate sample quality prior to library construction. In addition, virus populations in placental tissue from one control group monkey and two standard pretreatment group monkeys, as well as tissues from one congenitally infected fetus (from a standard pretreatment group dam) were successfully sequenced **(S2 Table)**.

RhCMV viral load was quantified from each sample using qPCR, as described in [9]. For all samples, multiple viral load measurements (3 to 18) were taken to ensure that samples with relatively low levels of virus present were identified as being positive for RhCMV. Viral load on the log_10_ scale was calculated as the mean of the individual log_10_ viral load sample measurements. When viral load was below the limit of detection (100 viral copies per ml for plasma and amniotic fluid and 100 viral copies per total DNA μg for urine and saliva), we set its value to half of the detection limit.

### Animal study ethics statement

The animal protocol titled “Maternal immune correlates with protection against congenital cytomegalovirus transmission in rhesus monkeys” was approved by the Tulane University and the Duke University Medical Center Institutional Animal Care and Use Committees (IACUC) under the protocol numbers P0285 and A186-15-06, respectively. Indian-origin rhesus macaques were housed at the Tulane National Primate Research Center and maintained in accordance with institutional and federal guidelines for the care and use of laboratory animals, specifically the USDA Animal Welfare regulations, PHS Policy on Humane Care and Use of Laboratory Animals[36], the NIH/NRC Guide for the Care and Use of Laboratory Animals, Association for Assessment and Accreditation of Laboratory Animal Care accreditation guidelines, as well as Tulane University and Duke University IACUC care and use policies. Tulane National Primate Research Center has strict policies to minimize pain and distress. The monkeys were observed on a daily basis and were administered tiletamine/zolazepam (Telazol), or ketamine if they showed signs of discomfort, pain or distress. In case of illness, the protocol involved analgesics administration and supplemental nutritional support and/or fluid therapy as needed.

Housing conditions were determined by the time and type of RhCMV inoculation, aiming to avoid horizontal transmission of RhCMV from other colony members, where RhCMV is endemic. RhCMV-seronegative pregnant macaques were housed in pairs after RhCMV inoculation if inoculated concurrently with the same viral isolate. Otherwise, single housing in BL2 containment facilities was required. The monkeys were maintained in a standard environment enrichment setting which included manipulable items, swings, food supplements (fruit, vegetables, treats), task-oriented feeding methods as well as human interaction with caretakers and research staff. Dams were released into the colony after 2 or 3 weeks following C-section.

Anesthesia was considered for all procedures considered to cause more than slight pain in humans, including routine sample collection. The agents used included: ketamine, butorphanol, Telazol, buprenorphine, carprofen, meloxicam, and midazolam as needed. The criteria for end-point was defined as loss of 25% of body weight from maximum body weight during protocol, major organ failure or medical conditions unresponsive to treatment and surgical complications unresponsive to immediate intervention. Policies stated that animals deemed at endpoint would be euthanized by overdose of pentobarbital under the direction of the attending veterinarian, consistent with the recommendations of the American Veterinary Medical Association guidelines on euthanasia.

### PCR amplification, viral sequencing, and analysis pipeline

We PCR-amplified two variable regions within the genes encoding glycoprotein *B* (gB) and glycoprotein *L (gL)* of RhCMV for subsequent next-generation sequencing. The *gB* amplicon was 408 nucleotides long and the *gL* amplicon 399 nucleotides long. The primer sequences for each amplicon are specified in [9]. The *gB* and *gL* regions sequenced for this study were selected to amplify all three strains present in the inoculum (UCD52, UCD59, and 180.92) in an unbiased manner. Indeed, we previously confirmed the absence of primer bias against these strains [9]. By sequencing two regions of approximately 400 nucleotides, we were able to identify *gB* and *gL* haplotypes instead of characterizing variants across a number of sites without knowledge of their linkage. For each of a given sample’s two amplified loci, our goal was to process two technical replicates. A small number of samples (6 in total), however, only had a single successfully sequenced replicate, while several samples had more than two successfully sequenced replicates (**S1 and S2 Tables**). As described previously [9], each replicate sample was independently PCR-amplified and sequenced following library preparation. All plasma samples required only a single round of PCR, whereas the samples from urine, saliva, amniotic fluid, placental tissues, and fetal tissues all required nested PCR. All replicates were sequenced on an Illumina MiSeq platform, using paired end reads of 300 bases.

To identify viral haplotypes and quantify their frequencies, we first used PEAR [37] to reconstruct (for each available technical replicate) the targeted locus by merging the paired-end reads corresponding to each sequenced fragment. The fused reads were then filtered using the *extractor* tool from the SeekDeep pipeline [38], which filters sequences according to their length, overall quality scores, and presence of primer sequences. Haplotype reconstruction for a given technical replicate was performed on the filtered sequences using the *qluster* tool from SeekDeep, which performs an iterative process of removing spurious, low abundance sequence groups by adding them to more abundant, genetically similar sequence groups when the genetic mismatch between groups occurs at nucleotide positions with low quality.

To obtain a set of haplotypes and their frequencies for a given sample, we combined identified haplotypes across technical replicates. Specifically, for a haplotype to be considered present in a sample, we required it to be detected in both sample replicates. Haplotypes that did not meet this criterion were merged with their genetically-closest haplotype in the sample, and the count of this genetically closest haplotype in the sample was increased accordingly. When only a single replicate was available, we could not perform this step and therefore kept all identified haplotypes present in the single available replicate. When more than two technical replicates were available, we restricted our analyses to the two replicates that were the most similar to one another genetically, based on correlation of haplotype frequencies (see below).

### Quality assurance and error reduction in sequencing data

We performed additional tests and required additional criteria to be met to ensure the quality of each sample that would undergo subsequent analysis. First, to reduce the number of spurious haplotypes in a given sample, we set a frequency threshold that sample haplotypes were required to exceed. This threshold was set to 0.436% based on analysis of plasmid controls. Specifically, we constructed two synthetic plasmids, one containing the *gB* gene and the other containing the *gL* gene. Two technical replicates from each plasmid were sequenced using the same protocol as for the RhCMV samples. Plasmids were run separately from the monkey samples. Because a single haplotype should be present in these plasmid control populations, any shared low-frequency haplotype is likely a product of PCR amplification error or sequencing error. We found 19 minor haplotypes in the *gB* plasmid control sample after merging technical replicates. These haplotypes ranged in frequency from 0.01% to 0.59% (**S1 Fig**). We found 29 minor haplotypes in the *gL* plasmid control sample after merging technical replicates. These haplotypes ranged in frequency from 0.03% to 0.42% (**S1 Fig**). Our chosen frequency threshold of 0.436% was set at the 0.95 quantile of the combined minor haplotype distributions from the *gB* and *gL* plasmids.

As a second quality assurance step, we performed chimera detection on the haplotypes in each merged sample. A haplotype was classified as a chimera if there was a combination of partial alignments to two observed (and higher frequency) haplotypes in the same sample. Detected chimeras were discarded. These chimeras contributed to only a small fraction of the total reads in each sample (ranging from 1.95% to 7.82% of the reads across all samples).

As a third quality assurance step, we restricted our analysis to those samples that had a Pearson correlation score exceeding 0.70 between the frequencies of the shared haplotypes across technical replicates on the log_10_ scale. For those samples with only a single technical replicate, we could not perform this step and instead included the sample in our analysis only if the read count exceeded 5000.

**S1 Table** shows the final set of maternal tissue and amniotic fluid samples that were included in our analyses, for both the *gB* and the *gL* loci. **S2 Table** shows the set of samples from the maternal-fetal interface (other than the amniotic fluid samples) and from fetal samples that were included in our analyses. In addition to these samples, the genetic composition of the inoculum was analyzed. Each of the three viral stocks comprising the inoculum (UCD52, UCD59, 180.92) was independently sequenced. Two successfully sequenced replicates were available for each of the three stock samples.

### Strain classification and nucleotide diversity calculations

Each identified haplotype in a sample was classified as belonging to one of the three strains that comprised the inoculum (UCD52, UCD59, or 180.92) based on its genetic distance to the reference sequences of these three strains. The reference sequences of the targeted *gB* and *gL* regions were obtained from [32] for strains UCD52 and UCD59 and from [39] for strain 180.92. Nucleotide diversity *π* present in a sample was calculated for each strain independently using the commonly used Nei-Gojobori equation, as described in [40]. All identified haplotypes, across all sequenced samples, are listed in **S1 Appendix and S2 Appendix**. The frequencies of these haplotypes in each of the sequenced samples, including the inoculum, are given in **S3 Appendix and S4 Appendix.**

### Statistical analysis and software

Data processing, analysis, and visualization were performed in R. Pairwise comparisons between groups were performed using non-parametric tests as indicated. For the visualization of the haplotype networks, we employed the R package *RCy3* version 1.2.0 that interfaces R 3.4 with Cytoscape.

### Data and code availability

Sequence data in fastq format from all the samples are available in SRA under the Bioproject PRJNA386504. All the R code used in the analysis of the sequence data is publicly available on GitHub: dverac/SNAPP.

## Results

### Maternal viral load dynamics, congenital transmission, and strain dominance

As previously described in [9], dams in the high-potency HIG pretreatment group had reduced peak viral loads in maternal plasma relative to dams in the control group following primary maternal infection (**S2A Fig**). Viral kinetics in the saliva and urine were also delayed in the high-potency pretreatment group compared to the control group (**S2C and S2D Fig**) [9]. Interestingly, and as previously noted [9], only dams with a peak plasma viral load exceeding 5.0 log_10_ viral copies/mL transmitted the virus to the amniotic fluid compartment. This included all 5 dams in the control group, 2 out of 3 dams in the standard pretreatment group, but none of the 3 dams in the high-potency pretreatment group. Viral dynamics in the amniotic fluid, when detectable, did not appear to differ between the control group monkeys and the standard HIG pretreatment group monkeys (**S2B Fig**).

Of the three viral strains used in the RhCMV inoculum, UCD52 became dominant in the overwhelming majority of tissue compartments, regardless of pretreatment group status (**S3 Fig**) [9]. Because of this, it is likely that UCD52 has higher viral fitness *in vivo*, relative to both UCD59 and 180.92. The reason for UCD52 dominance over UCD59 is unknown: RhCMV-naïve macaques inoculated with UCD52 and UCD59 have similar patterns of viremia and shedding in bodily fluids [32–34]. That strain 180.92 did not become dominant is not surprising given previous findings of only low-level viral shedding and limited tissue dissemination of this strain in inoculated RhCMV-naïve macaques [35]. Given the dominance of the UCD52 strain in the overwhelming majority of samples, we focused our remaining analyses on haplotypes that were classified as belonging to the dominant UCD52 strain.

### Minor RhCMV haplotypes and levels of genetic diversity during acute maternal infection

Across the majority of analyzed samples, we found that the dominant *in vivo* UCD52 haplotype was the canonical UCD52 reference haplotype of the viral inoculum. This was the case both for the *gB* locus and the *gL* locus, and across all groups and compartments studied.

**Fig 1:**
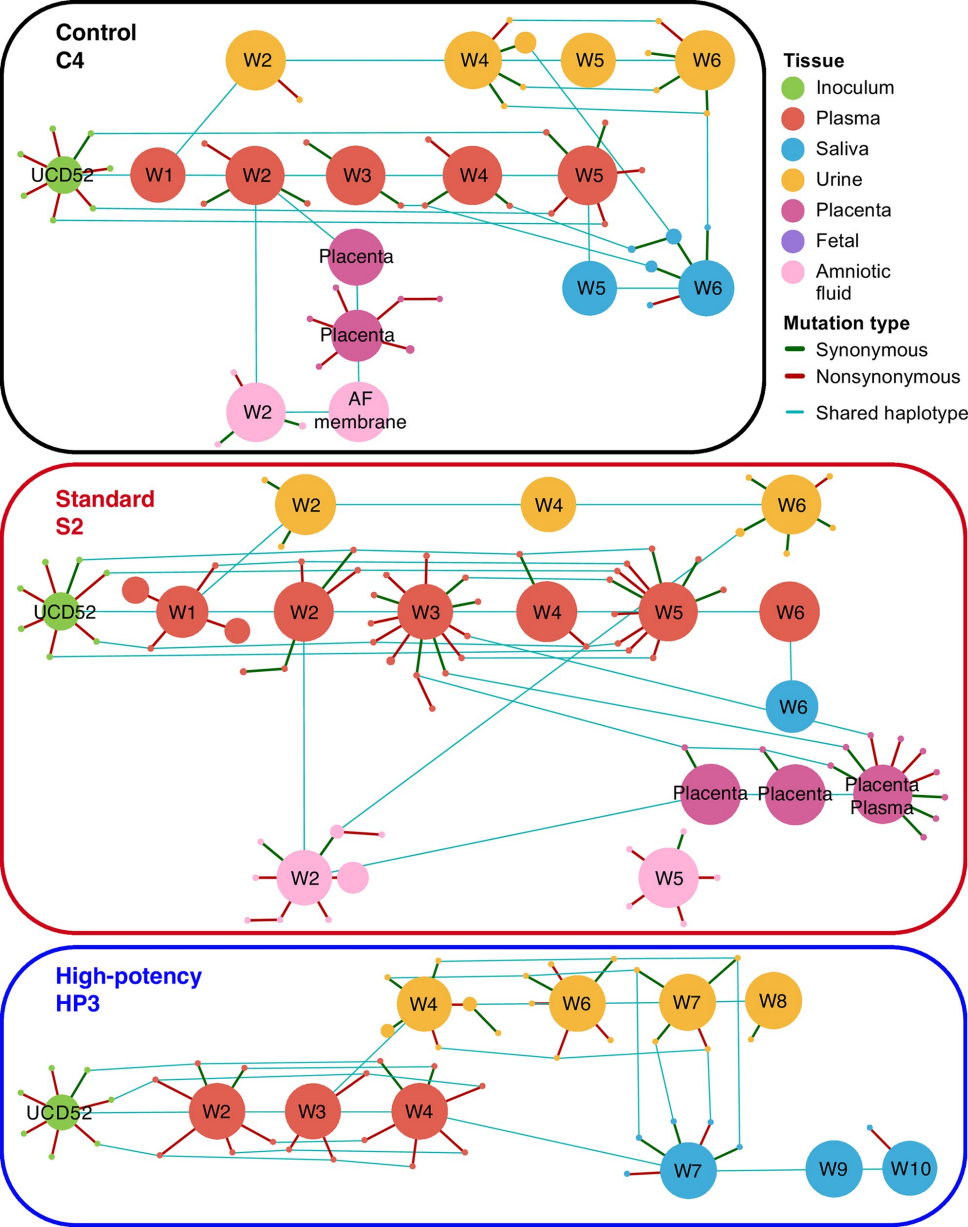
UCD52 haplotype networks for the *gB* locus across sampled tissues from three representative monkeys. Haplotype networks are shown for one control group dam (C4), one standard pretreatment group dam (S2), and one high-potency pretreatment group dam (HP3). Unique haplotypes are shown as circles (nodes). Node sizes scale with haplotype frequency. Green and red lines (edges) connect haplotypes that differ by a single nucleotide. Green edges depict synonymous mutations and red edges depict nonsynonymous mutations. Samples are labeled by collection week. Blue lines connect shared haplotypes across samples. The UCD52 inoculum stock is included in the haplotype network of each monkey.

**Fig 2:**
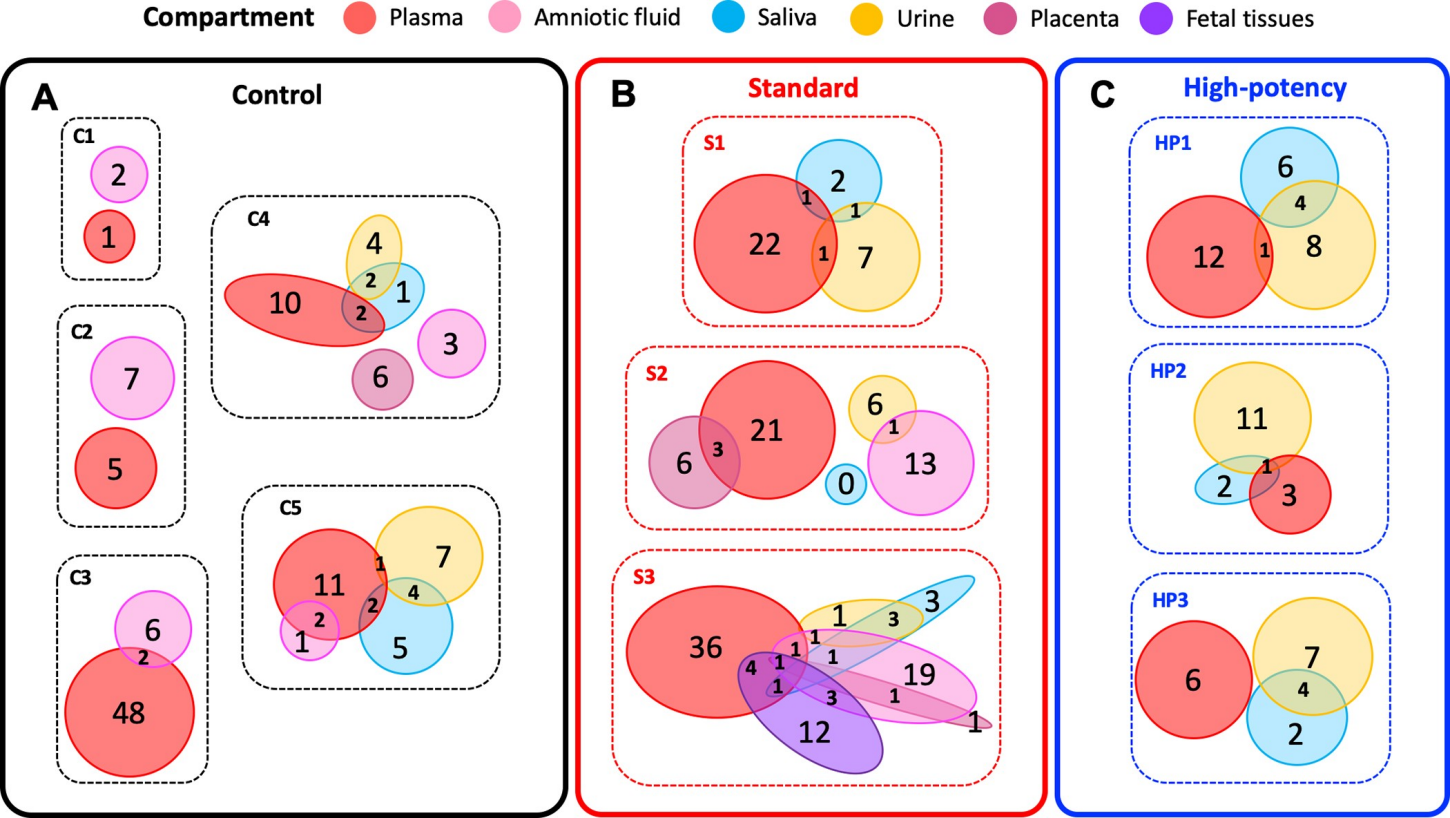
The number of minor UCD52 haplotypes at the *gB* locus that are either shared or unique across compartments, by monkey. Here, the set of minor haplotypes for a given compartment includes all timepoint samples from that compartment. Patterns of minor haplotype sharing for (A) control group monkeys, (B) standard pretreatment group monkeys, and (C) high-potency pretreatment group monkeys. Compartments are color-coded as in Fig 1.

Our analysis of amplified sequences from the *gB* locus identified 7 minor haplotypes in the UCD52 inoculum, as well as a large number of minor haplotypes in maternal and fetal compartments (**S3 Appendix**, **Fig 1**, **S4–S11 Figs**). The number of haplotypes identified in a sample was not positively correlated with the sample’s viral load (**S12 Fig**), indicating that the number of observed haplotypes was not restricted by sample viral load. The identified minor haplotypes differed from the canonical UCD52 *gB* haplotype by typically only a single (either synonymous or nonsynonymous) nucleotide mutation. These minor haplotypes ranged in frequency from just above the sequencing error cut-off frequency of 0.436% up to 43.27%, with a median frequency of 0.80%. Maternal samples differed in the number of identified *gB* haplotypes they contained, ranging from 1 to 33, with a median of 5 haplotypes per sample. Minor haplotypes identified in the maternal samples that were not identified in the UCD52 inoculum potentially arose through intrahost *de novo* mutation. Alternatively, these minor haplotypes may have been present in the inoculum at frequencies below 0.436%. Within individual dams, we observed that some of the minor haplotypes were shared across timepoints from the same compartment and/or across compartments (**Fig 1**, **S4–S11 Figs**). This finding indicates that some of these minor haplotypes persist over a timespan of weeks in a given compartment and that some of these minor haplotypes are likely transmitted across anatomic compartments. Of the minor haplotypes that were shared across compartments, most were shared between the plasma and one other compartment (**Fig 1**, **Fig 2**, **S4–S11 Figs**). This pattern may be due to plasma being a source of viral haplotypes for other compartments; alternatively, it may simply be due to a larger number of plasma samples being successfully sequenced relative to those from other compartments (**S1 Table**). Interestingly, in 6 out of the 8 monkeys that had both urine and saliva sequences available, there were also minor *gB* haplotypes that appeared to be shared exclusively between urine and saliva samples. These haplotypes were generally found first in urine and then in a later week in the saliva, suggesting potential oral auto-inoculation from virus shed in urine.

**Fig 3:**
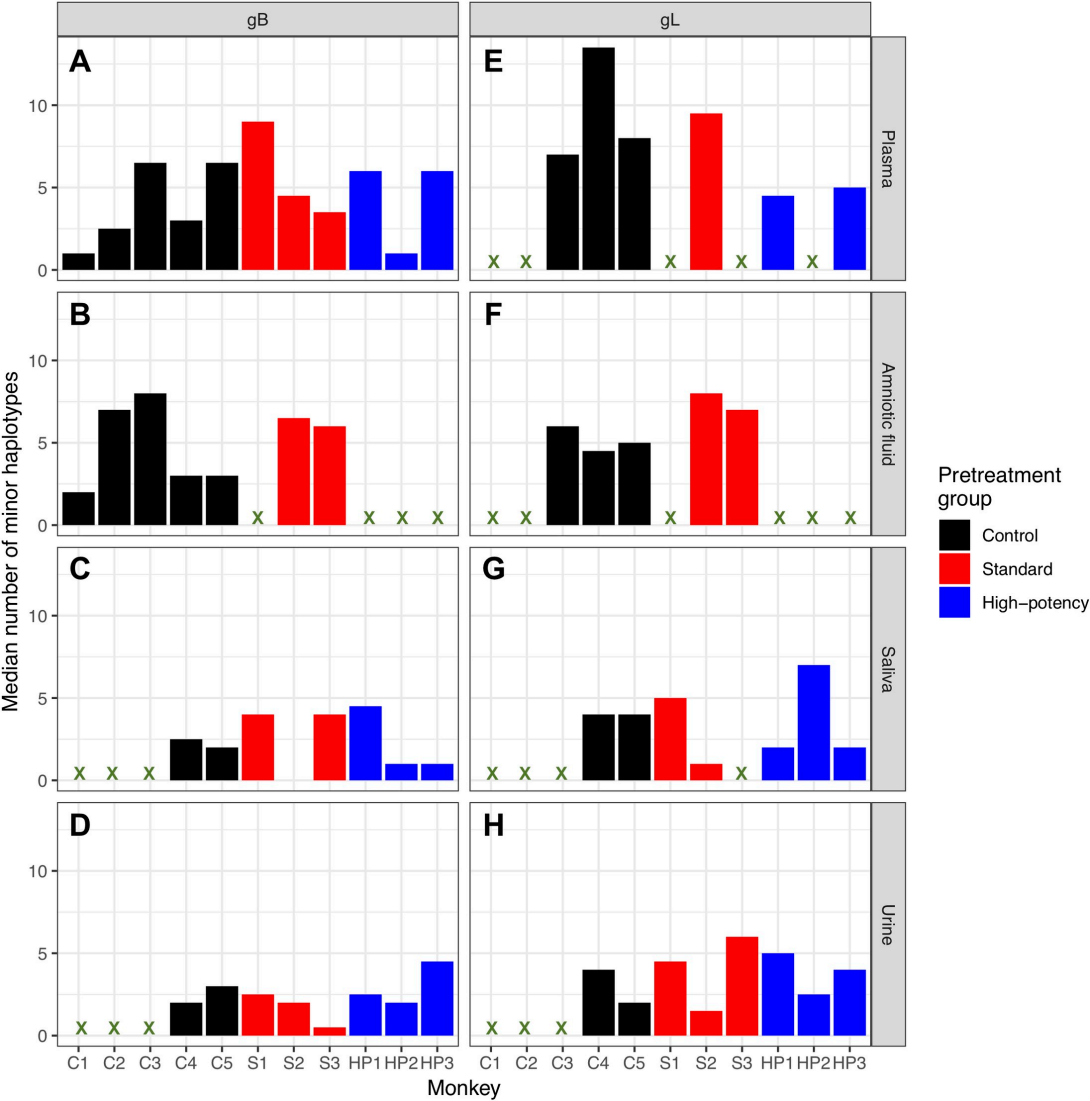
Median number of minor haplotypes, by locus, tissue, and pretreatment group. The panels A,B,C and D show the median number of *gB* minor haplotypes; panels E,F,G and H show the median number of *gL* minor haplotypes. Rows show tissues: plasma (A,E), amniotic fluid (B,F), saliva (C,G), and urine (D,H). Marker symbols correspond with those in S2 Fig. We found no significant differences in the median number of minor haplotypes across pretreatment groups for any tissue, at either locus (Mann Whitney test; *p* > 0.1).

To assess whether the number of identified *gB* haplotypes differed by pretreatment group, we calculated the median number of minor UCD52 haplotypes in each available tissue for each of the 11 dams. This quantity reflects a measure of haplotype richness. We found no significant differences in the median number of minor *gB* haplotypes by tissue across any pair of pretreatment groups (all Mann-Whitney *U* tests, *p* > 0.1; **Fig 3A–3D**). We further calculated the proportion of minor *gB* haplotypes that differed from the canonical haplotype by at least one nonsynonymous mutation, by tissue and monkey. No significant differences between these proportions were found between tissues, indicating no clear trend of positive selection or relaxed purifying selection in certain tissues over others at the *gB* locus. No significant differences between these proportions were found between pretreatment groups on a tissue-by-tissue basis, again indicating no clear trend of positive selection or relaxed purifying selection in one group over others at the *gB* locus.

The UCD52 haplotype patterns observed at the *gL* locus are consistent with those at the *gB* locus. Specifically, minor *gL* haplotypes generally differed from one of the two dominant *gL* haplotypes present in the inoculum by a single nucleotide (**S13–S22 Figs**). Similar to the frequencies observed for *gB* haplotypes, minor *gL* haplotypes were present at frequencies as low as 0.44% and up to 48.16%, with a median frequency of 1.05%. Samples differed in the number of identified minor *gL* haplotypes they contained, ranging from 2 to 29 with a median of 6 minor haplotypes per sample. Again, no correlation was found between the number of haplotypes identified in a sample and the sample’s viral load (**S23 Fig**). Some of the identified minor *gL* haplotypes appeared to persist within the same tissue over time, and some were shared across tissue compartments. Similar to our findings at the *gB* locus, most of the minor haplotypes that were shared across compartments were shared between the plasma and one other compartment (**S24 Fig**). Minor *gL* haplotypes shared between urine and saliva compartments again suggested auto-inoculation. Finally, consistent with the findings from the *gB* locus, the median number of *gL* minor haplotypes observed in any tissue did not differ between pretreatment groups (**Fig 3**). We again found no significant differences between tissues or pretreatment groups in the proportion of minor *gL* haplotypes that differed from the canonical reference haplotype by at least one nonsynonymous mutation, consistent with the lack of pattern at the *gB* locus.

**Fig 4:**
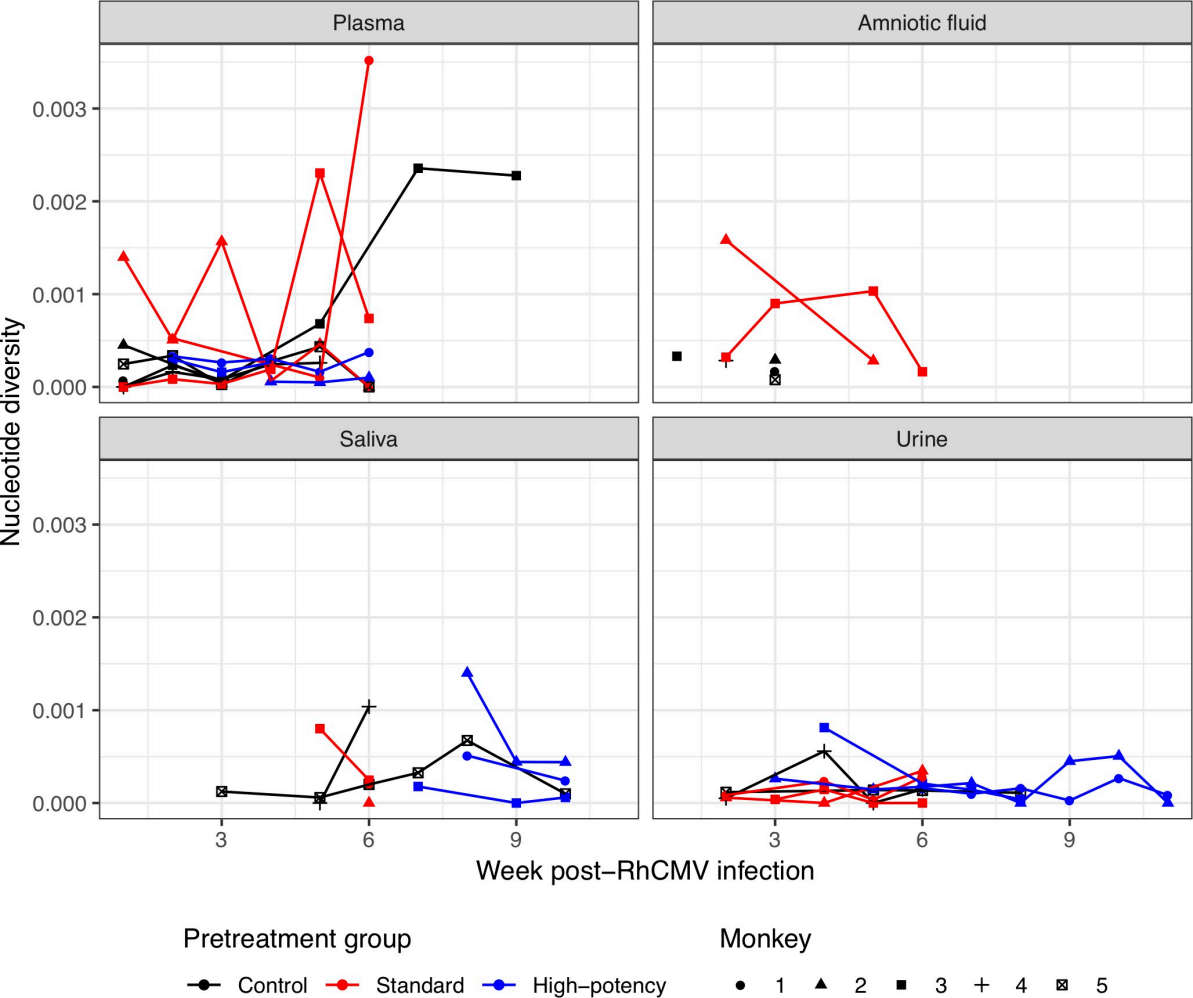
Pairwise genetic diversity *π* over time, by tissue, for the *gB* locus. Levels of UCD52 genetic diversity in each monkey varied over time, for each tissue. Marker symbols correspond with those in S2 Fig.

We next assessed whether HIG pretreatment had an impact on RhCMV genetic diversity, as measured by pairwise nucleotide diversity *π* for each sample’s UCD52 viral population. Levels of viral genetic diversity varied significantly between monkeys, compartments, and across weeks (**Fig 4** for *gB*, **S25 Fig** for *gL*). Despite this variation, median levels of *gB* viral genetic diversity did not differ by pretreatment group for any tissue (all Mann-Whitney U tests, *p* > 0.1) besides the amniotic fluid. In this compartment, median levels of *gB* viral genetic diversity appeared to be slightly higher in standard pretreatment group monkeys than in control animals (Mann-Whitney U test, *p* = 0.095). Median levels of *gL* viral genetic diversity did not differ by pretreatment group for any tissue (all Mann-Whitney U tests > 0.1; **S25 Fig**).

Because error rates may be variable across samples due to differences in PCR protocol (in particular, single versus nested PCR), and because the plasmid controls may underestimate the error rate of the samples due to higher template copy numbers in the plasmid controls, we reanalyzed the sequence data at both the *gB* and the *gL* loci using a more conservative threshold of 0.88% (double that of the 0.436% threshold). At this more conservative threshold, we identified fewer minor haplotypes and arrived at lower levels of genetic diversity. However, the conclusions we arrived at using the 0.436% threshold were robust to the more conservative threshold of 0.88%. Specifically, there was evidence for some minor haplotypes persisting across time in a given tissue, and for some minor haplotypes being shared between compartments (**S5 Appendix**). The extent to which minor haplotypes were shared across samples from the same dam was reduced, but still noticeable, particularly at the *gL* locus. There were no significant differences in levels of RhCMV genetic diversity by HIG pretreatment group or by tissue compartment, with the exception of viral genetic diversity in the amniotic fluid being slightly higher in the standard pretreatment group monkeys than those of the control group (Mann-Whitney U test, *p* = 0.095). As a further sensitivity analysis, we reanalyzed the sequence data at both the *gB* and the *gL* locus using a less conservative threshold of 0.22% (half that of the 0.436% threshold). At this 0.22% threshold, we identified a larger number of minor haplotypes and arrived at slightly higher levels of genetic diversity. However, the conclusions we arrived at using the 0.436% threshold were again robust at the less conservative threshold of 0.22%. There was extensive evidence for minor haplotypes persisting across time in a given tissue, and for some minor haplotypes being shared between compartments (**S5 Appendix**). Again, there were no significant differences in levels of RhCMV genetic diversity by HIG pretreatment group or by tissue compartment, with the exception of viral genetic diversity in the amniotic fluid being slightly higher in the standard pretreatment group monkeys than those of the control group (*p* = 0.095).

### Genetic diversity and compartmentalization of maternal RhCMV variants identified in placenta and amniotic fluid

We next sought to determine the extent to which minor UCD52 haplotypes were shared between maternal compartments and compartments comprising the maternal-fetal interface (amniotic fluid and placental tissues). As reported above, we found that some minor *gB* and *gL* UCD52 haplotypes were shared between maternal plasma samples and amniotic fluid samples (**Figs 1** and **2**; **S3 Appendix** and **S4 Appendix)**. Similarly, some of the minor *gB* and *gL* UCD52 haplotypes found in placental tissue samples were also present in maternal plasma samples (**Fig 2**). Specifically, between placental tissues and plasma samples, we observed 6 shared minor *gL* haplotypes in C4 (**S15 Fig**), 3 shared minor *gB* haplotypes in S2 (**Fig 1**), 1 shared minor *gL* haplotype in S2 (**S18 Fig**), and 1 shared minor *gB* haplotype in S3 (**S9 Fig**). As these minor shared haplotypes are mostly present at marginal frequencies in maternal tissues (median frequency in plasma for shared haplotypes: 1.28%, minimum 0.47%, in *gB*, S2; maximum 24.05% in *gL*, S2) (**S3 Appendix** and **S4 Appendix)**, the bottleneck between mother and placental tissues is likely relatively large. This reasoning is based on the observation that transmission of low-frequency minor haplotypes would only be evident if the number of virions transmitting is large [41,42], that is, when the transmission bottleneck is loose. Since multiple low-frequency minor haplotypes appear to be shared between maternal plasma and placental tissue samples, and each of these haplotypes is unlikely to transmit between tissues unless the bottleneck is loose, this strongly indicates that the number of virions reaching placental tissues is considerably high. Interestingly, a large number of minor *gB* and *gL* UCD52 haplotypes were found in amniotic fluid samples (**Fig 2**, **S24 Fig**; **S3 Appendix** and **S4 Appendix)**, most of which were not observed in maternal tissues. This indicates that *de novo* viral mutations may occur in the fetus and subsequently be shed into the amniotic fluid. In the one case in which placental plasma was available for analysis (dam S2 in **Fig 1**; **S18 Fig**), we found considerably more minor haplotypes in both *gB* and *gL* gene regions in this sample compared with the paired placental tissue and many of these minor haplotypes were not observed in maternal plasma.

### Genetic diversity and compartmentalization of fetal RhCMV variants

**Fig 5:**
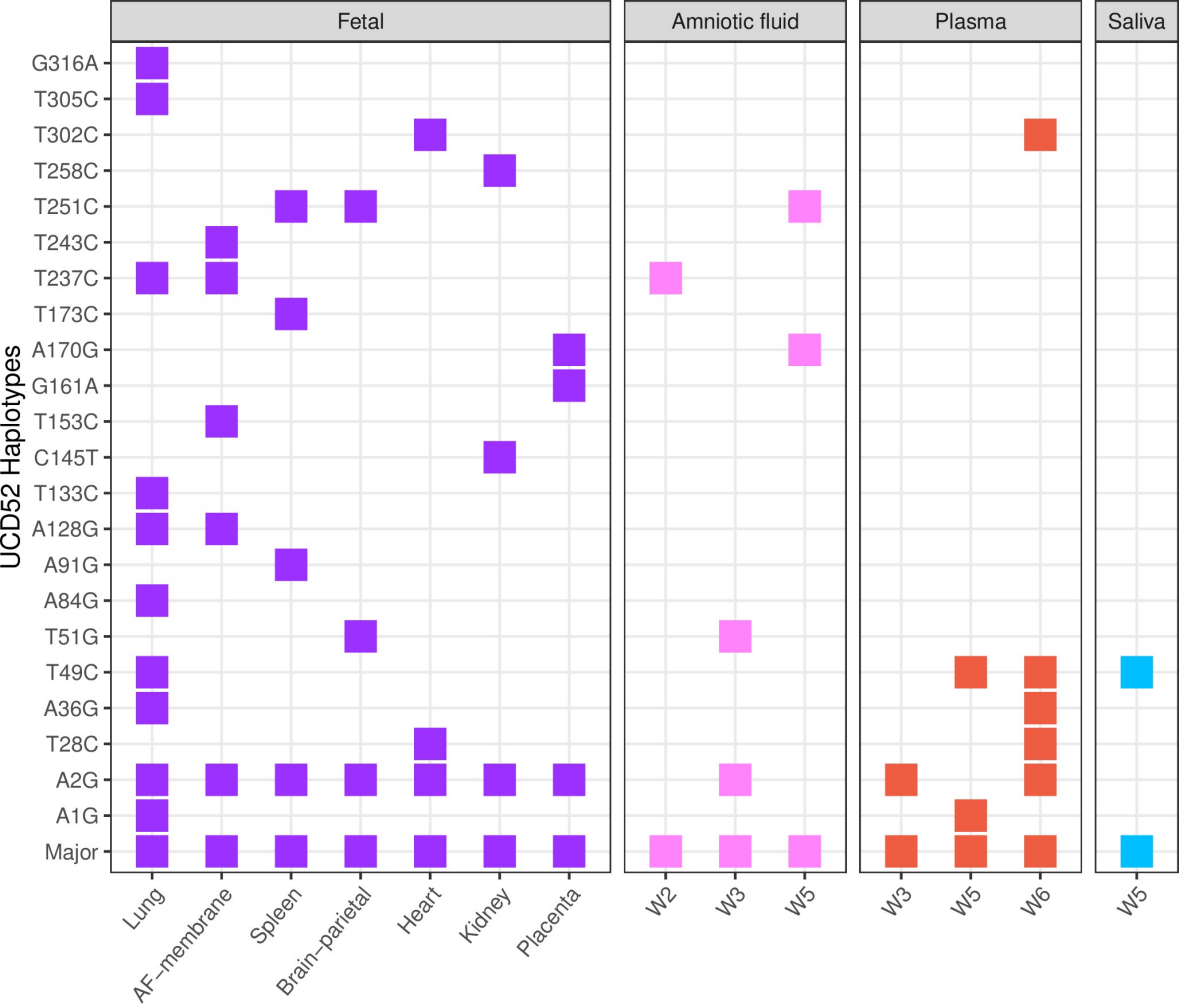
Minor UCD52 haplotypes found in fetal tissues, and their presence in maternal compartments of dam S3. Each row depicts a haplotype found in at least one fetal tissue (purple), harvested at 6 weeks post-RhCMV infection. Rows are ordered by their mutated nucleotide position when compared to the major haplotype, which is located in the bottom row. A square denotes that a given haplotype was found in a given sample.

Congenital infection was confirmed in two of three dams in the standard pretreatment group and in all five control dams, whereas congenital infection did not occur in any of the three high-potency pretreatment group dams. All five control dams experienced fetal loss within 2–3 weeks of maternal infection and fetal tissues were often not recovered. In standard pretreatment group dam S3, nearly all the fetal tissues harvested at 6 weeks post-RhCMV infection tested positive for RhCMV, including fetal lung, brain, kidney, spleen, heart, placenta, amniotic fluid, and amniotic membrane. Similar to our observation in the maternal tissue compartments, a single major UCD52 haplotype (the canonical reference haplotype) was present in all fetal tissue samples. Multiple minor UCD52 haplotypes were also detected in these samples (**S9 Fig**; **Fig 5**). Intriguingly, a second, minor haplotype was found across all the fetal tissues, present at low frequencies of ≤1%. This haplotype was also observed in one of the paired dam’s three amniotic fluid samples (week 3, frequency of 0.8%), placenta (frequency of 0.85%), and in two of the paired dam’s plasma samples (at weeks 3 and 6; frequencies of 0.58% and 0.66%, respectively) (**Fig 5**). Of the remaining 21 minor UCD52 haplotypes in fetal tissues, 4 were also present in amniotic fluid samples and 5 in plasma samples of paired dam S3 (**Fig 5**). Plasma haplotypes detected as late as weeks 5 and 6 post-inoculation contribute to those shared haplotypes. In an attempt to gauge whether the minor fetal haplotypes may have all already been present in the viral inoculum, we determined the overlap between minor haplotypes in the inoculum and those in the fetal samples: none of the minor fetal haplotypes were identified in the UCD52 inoculum. Moreover, we found that only 0–5 minor haplotypes were shared between the fetal tissues and non-paired dams. In comparison, 10 minor haplotypes were shared between fetal tissues and maternal plasma/amniotic fluid samples from the paired dam S3. These findings indicate that the minor haplotypes identified in fetal tissues likely originated *de novo*, either in the fetal tissues themselves or in maternal tissues.

To determine whether patterns of genetic diversity were similar between fetal tissues and other tissues, we then calculated pairwise genetic diversity *π* from each available fetal tissue. We observed lower diversity in the fetal tissues compared to that in both the amniotic fluid (*p* = 0.025) and the plasma at late weeks post-infection (Weeks 4 to 6, *p* = 0.095). We further observed higher diversity in the fetal tissues compared to that in plasma during the first three weeks post-infection (Weeks 1 to 3, *p* = 0.024). These observations together again suggest that the maternal viral population contributes to viral diversity in the fetus and that congenital transmission may be subject to a loose bottleneck. Transmission of RhCMV from maternal plasma to fetal tissues may also occur over an extended period of time, as minor low-frequency haplotypes identified in fetal tissues were shared with those identified in maternal plasma samples between weeks 3 and 6 (**S9 Fig**).

## Discussion

In this study, we characterized the population genetics of RhCMV in a monkey model of congenital CMV transmission and examined the impact of preexisting maternal virus-specific antibodies on maternal and fetal viral populations. For this study, we used amplicon sequencing of two distinct genetic loci within the genes encoding RhCMV glycoproteins B and L. Unique aspects of this study include serial sampling from multiple maternal compartments over the time course of acute RhCMV infection, sequencing of RhCMV-infected tissues at the maternal-fetal interface, and sequencing of RhCMV from fetal tissues. We found that the overwhelming majority of maternal and fetal tissue samples were dominated by UCD52. The reason for the dominance of this singular strain across HIG pretreatment groups is unclear, although it undoubtedly indicates that UCD52 is genetically more effective at *in vivo* replication than either of the co-inoculated variants UCD59 and 180.92. Given the dominance of UCD52 in all groups and tissues, we focused subsequent analyses on characterizing the genetic variation of this strain in available samples. In the majority of samples from maternal tissues, maternal-fetal interface, and fetal tissues, the major *gB* and *gL* UCD52 variant detected was the canonical UCD52 reference sequence. However, most samples also had minor, low-frequency UCD52 haplotypes present, with some of these minor haplotypes persisting over time within a tissue and occasionally shared between tissues.

Although monkeys that received a high-potency antibody preparation had significantly lower peak viral loads compared to control group monkeys, in our analysis we found no evidence for a relationship between HIG pretreatment and the number of minor UCD52 haplotypes present in maternal plasma. We further found no evidence for a relationship between HIG pretreatment and levels of UCD52 nucleotide diversity. Together, these results suggest that preexisting antibodies can reduce overall viral load but do not appear to restrict replication of specific UCD52 viral variants or limit viral diversity in the two loci we studied, *gB* and *gL*. We also found no significant differences between the three groups when comparing the number of minor UCD52 haplotypes found in either saliva or urine, at either the *gB* or the *gL* locus. While we previously assessed and reported lower *maximum* plasma viral diversity levels in monkeys pretreated with HIG compared to the control group [9], here, we included a more in-depth analysis across timepoints to report the *median* viral diversity levels across monkeys, and did not find any lasting impact of preexisting antibodies on maternal viral diversity.

Our identification of shared, minor UCD52 haplotypes between maternal plasma samples, amniotic fluid, placental tissue, and fetal tissues is consistent with previous studies investigating the population genetics of HCMV in newborns [19,43], which together point towards a large number of virions being vertically transmitted between mother and fetus. While previous studies have indirectly estimated vertical transmission bottleneck sizes for HCMV by inferring effective population size [21,44], in this study we were unable to quantify transmission bottleneck sizes between mother and fetus due to low levels of haplotype diversity and noise arising from haplotype frequencies being near the limit of detection threshold. Nevertheless, based on the identification of minor UCD52 haplotypes across maternal, maternal-fetal interface, and fetal tissues, our analysis suggests that diversity in a given tissue is likely generated through a combination of viral haplotypes being passed to that compartment, along with *de novo*, local generation of viral mutations.

Recently, Sackman and coauthors proposed a model for congenital HCMV transmission that involves two successive transmission events: maternal virus infection of placental tissues followed by continued transmission of the placental viral population to fetal circulation [45]. This model is supported by observations of the sustained presence of HCMV in the placenta and umbilical cord, which would potentially allow for transmission between placental tissues and fetal tissues over a longer time interval [46]. Our results are consistent with this proposed model. Specifically, in our analysis of fetal samples from dam S3, we identified minor haplotypes in fetal tissues that were also present in maternal plasma at various time-points during infection (**S9 Fig**). Furthermore, we observed that multiple haplotypes in this dam’s amniotic fluid were also observed in maternal plasma and other maternal compartments. Since amniotic fluid haplotypes derive from both intrauterine and fetal viral populations, this finding again provides support for a loose transmission bottleneck from mother to fetus.

Our conclusions are limited by multiple factors. First, as is common for experimental monkey challenge studies and particular to studies of a selective colony of RhCMV seronegative breeding animals, we are limited by the small number of animals in each group and by sample availability. PCR amplification failure further limited the number of samples available for analysis. Second, only a very small portion of the RhCMV genome was sequenced. The *gB* and *gL* regions of approximately 400 nucleotides long sequenced in this study were selected to amplify all three inoculum strains in an unbiased manner. This approach allowed us to identify complete haplotypes rather than single variants, such that we had full linkage information across the sequenced regions. It also allowed us to sequence samples with low levels of virus and to identify low frequency haplotypes. Unfortunately, sequencing of such a limited region of the RhCMV genome does not allow us to assess any potential impact of HIG pretreatment on alternate RhCMV loci that encode proteins known to be targeted by antibodies (e.g. *gM*/*gN*, *gO*, *gH*, and UL128-131a) [47–50].

Our ability to draw conclusions was further limited by the low levels of UCD52 genetic diversity observed in the samples. We used highly conservative haplotype-calling and error reduction methods to ensure that the haplotypes we identified were not false positives. As a result, however, we likely excluded many true haplotypes, which reduced the diversity levels we characterized and limited our ability to make inferences regarding transmission bottleneck sizes. Importantly, however, our sensitivity analyses using 0.22% and 0.88% for our haplotype-calling threshold did not qualitatively change our results. The consistency of results across this wide range of haplotype-calling thresholds is also comforting given that both nested and single round PCR were used on the samples, and these two PCR approaches will almost certainly have different error rates. Finally, our animal model of congenital CMV transmission involves maternal CD4^+^ T cell depletion, which results in consistent placental transmission. Our results therefore might not be applicable to immunocompetent individuals.

Despite these limitations, we were able to conclude that minor haplotypes persisted over time within single maternal tissue compartments and that these minor haplotypes were occasionally shared between anatomic compartments. Moreover, the observation of minor haplotypes that were shared across plasma, placenta, amniotic fluid, and fetal tissues point towards a loose transmission bottleneck between maternal tissues and fetus-associated tissues. These findings are consistent with those from human congenital CMV cases [13,19,44,51,52].

Patterns of *gB* and *gL* viral diversity within and across compartments, however, did not appear to differ between HIG pretreatment groups. This finding indicates that, although potently-neutralizing CMV-specific antibodies can effectively reduce viral population size and prevent congenital transmission [9], preexisting HIG had no appreciable impact on the genetic makeup of the *gB* and *gL* loci in maternal RhCMV populations. These findings are interesting given the growing evidence that preexisting HCMV-specific antibodies can reduce the incidence and severity of congenital HCMV [9,53–55], perhaps suggesting a model wherein congenital virus transmission is dependent upon the overall quantity of maternal systemically-circulating virus rather than antibody selection of specific variants at the maternal-fetal interface. Further studies, ideally starting with an inoculum containing higher levels of viral diversity, may be required to provide a deeper understanding of the extent of antibody-mediated immune-pressure on CMV populations, as well as the effect of antibodies on viral transmission dynamics across the placenta. Results from these studies will be critical to more effectively anticipate the effect of CMV vaccines and therapeutic interventions on congenital CMV transmission potential and the propensity for this virus to evolutionarily circumvent these interventions.

## Supporting information

S1 Fig The use of synthetic plasmids to define a haplotype-calling threshold to exclude spurious haplotypes from samples. Minor haplotypes were identified from the synthetic plasmid control samples as described in the Methods section, for both the *gB* locus and the *gL* locus. The figure shows, for each locus, the fraction of identified minor haplotypes (y-axis) that fall at the haplotype frequency shown on the x-axis or below. The vertical red line shows the frequency threshold of 0.436% that was used to call minor haplotypes.
(TIF)

S2 Fig Viral load dynamics measured in dams experimentally infected with RhCMV. Virus was measured in (A) plasma, (B) amniotic fluid, (C) saliva, and (D) urine. Monkeys are color-coded according to pretreatment group: control (black), standard pretreatment group (red), and high-potency pretreatment group (blue). Monkey ID numbers correspond to those provided in S1 Table. Virus was detected in the plasma, saliva, and urine of all 11 monkeys. Virus was only detected in the amniotic fluid of the 5 control group monkeys and in 2 of the 3 standard HIG group monkeys. Viral load levels shown here are average values when more than one measurement was available (Methods).
(TIF)

S3 Fig Strain composition of the RhCMV population in various maternal compartments over time. The proportion of the RhCMV population belonging to strain UCD52 is shown for maternal plasma, saliva, and urine. Strain frequencies were calculated for the *gB* locus (left column) and for the *gL* locus (right column). Green squares in the plasma subplots denote the fraction of the viral inoculum that was UCD52 (25%).
(TIF)

S4 Fig Haplotype networks for the *gB* locus across sampled tissues from monkey C1. Colorcoding of nodes and edges are as in Fig 1, which show haplotype networks for C4, S2, and HP3.
(TIF)

S5 Fig Haplotype networks for the *gB* locus across sampled tissues from monkey C2. Colorcoding of nodes and edges are as in Fig 1, which show haplotype networks for C4, S2, and HP3.
(TIF)

S6 Fig Haplotype networks for the *gB* locus across sampled tissues from monkey C3. Colorcoding of nodes and edges are as in Fig 1, which show haplotype networks for C4, S2, and HP3.
(TIF)

S7 Fig Haplotype networks for the *gB* locus across sampled tissues from monkey C5. Colorcoding of nodes and edges are as in Fig 1, which show haplotype networks for C4, S2, and HP3.
(TIF)

S8 Fig Haplotype networks for the *gB* locus across sampled tissues from monkey S1. Colorcoding of nodes and edges are as in Fig 1, which show haplotype networks for C4, S2, and HP3.
(TIF)

S9 Fig Haplotype networks for the *gB* locus across sampled tissues from monkey S3. Colorcoding of nodes and edges are as in Fig 1, which show haplotype networks for C4, S2, and HP3.
(TIF)

S10 Fig Haplotype networks for the *gB* locus across sampled tissues from monkey HP1. Colorcoding of nodes and edges are as in Fig 1, which show haplotype networks for C4, S2, and HP3.
(TIF)

S11 Fig Haplotype networks for the *gB* locus across sampled tissues from monkey HP2. Colorcoding of nodes and edges are as in Fig 1, which show haplotype networks for C4, S2, and HP3.
(TIF)

S12 Fig The relationship between viral load and the number of *gB* haplotypes found in each sample. The correlation between viral load and the number of *gB* haplotypes was not significantly positive for any of the four analyzed compartments (plasma, amniotic fluid, saliva, urine).
(TIF)

S13 Fig Haplotype networks for the *gL* locus across sampled tissues from monkey C1. Colorcoding of nodes and edges are as in Fig 1.
(TIF)

S14 Fig Haplotype networks for the *gL* locus across sampled tissues from monkey C3. Colorcoding of nodes and edges are as in Fig 1.
(TIF)

S15 Fig Haplotype networks for the *gL* locus across sampled tissues from monkey C4. Colorcoding of nodes and edges are as in Fig 1.
(TIF)

S16 Fig Haplotype networks for the *gL* locus across sampled tissues from monkey C5. Colorcoding of nodes and edges are as in Fig 1.
(TIF)

S17 Fig Haplotype networks for the *gL* locus across sampled tissues from monkey S1. Colorcoding of nodes and edges are as in Fig 1.
(TIF)

S18 Fig Haplotype networks for the *gL* locus across sampled tissues from monkey S2. Colorcoding of nodes and edges are as in Fig 1.
(TIF)

S19 Fig Haplotype networks for the *gL* locus across sampled tissues from monkey S3. Colorcoding of nodes and edges are as in Fig 1.
(TIF)

S20 Fig Haplotype networks for the *gL* locus across sampled tissues from monkey HP1. Colorcoding of nodes and edges are as in Fig 1.
(TIF)

S21 Fig Haplotype networks for the *gL* locus across sampled tissues from monkey HP2. Colorcoding of nodes and edges are as in Fig 1.
(TIF)

S22 Fig Haplotype networks for the *gL* locus across sampled tissues from monkey HP3. Colorcoding of nodes and edges are as in Fig 1.
(TIF)

S23 Fig The relationship between viral load and the number of *gL* haplotypes found in each sample. The correlation between viral load and the number of *gL* haplotypes was not significantly positive for any of the four analyzed compartments (plasma, amniotic fluid, saliva, urine).
(TIF)

S24 Fig The number of UCD52 minor *gL* haplotypes that are either shared or unique across compartments, by monkey. Here, the set of minor haplotypes for a given compartment includes all timepoint samples from that compartment. Patterns of minor haplotype sharing for (A) control group monkeys, (B) standard pretreatment group monkeys, and (C) high-potency pretreatment group monkeys. Compartments are colorcoded as in Fig 1.
(TIF)

S25 Fig Pairwise genetic diversity *π* over time, by tissue, for the *gL* locus. Marker symbols correspond with those in S2 Fig.
(TIF)

S1 Table Sampled tissues and their times of sampling for each of the 11 studied dams. Dams are separated by pretreatment group: control (C1-C5), standard (S1-S3), and high-potency (HP1-HP3). In addition to the C1-C5, S1-S3, and HP1-HP3 identifiers, individual monkeys are identified according to names previously used in [9] and [27]. Cells are colored according to the legend provided. Text in the white-colored cells indicate which loci were successfully sequenced and included in our analyses (*gB* = glycoprotein B region; *gL* = glycoprotein L region). Numbers in the cells, when present, indicate the number of sample replicates that were available for analysis, when not two. A single round of PCR was used for all plasma samples. Nested PCR was used for all other samples.
(PDF)

S2 Table Fetal-maternal interface and fetal tissues analyzed in the study. All listed samples had two successfully sequenced replicates and underwent nested PCR.
(PDF)

S1 Appendix List of unique haplotypes identified at the *gB* locus across all samples, including the inoculum. Each row corresponds to a unique haplotype. Each haplotype has listed the strain it belongs to (UCD52, UCD59, or 180.92), an identifying haplotype number (e.g., H359), the number of mutations between it and the canonical reference strain, and the types of these mutations (synonymous or nonsynonymous).
(CSV)

S2 Appendix List of unique haplotypes identified at the *gL* locus across all samples, including the inoculum. Each row corresponds to a unique haplotype. Each haplotype has listed the strain it belongs to, an identifying haplotype number, the number of mutations between it and the canonical reference strain, and the types of these mutations.
(CSV)

S3 Appendix List of identified *gB* haplotypes, by sample. Samples are defined by the monkey ID, tissue of origin, and collection week post-RhCMV infection. The relative frequency of each haplotype in a sample is provided.
(CSV)

S4 Appendix List of identified *gL* haplotypes, by sample. Samples are defined by the monkey ID, tissue of origin, and collection week post-RhCMV infection. The relative frequency of each haplotype in a sample is provided.
(CSV)

S5 Appendix Haplotype-calling threshold sensitivity analysis. Each page shows the number and recurrence dynamics of minor UCD52 haplotypes in a single dam (or dam-fetus pair) at a single locus (*gB* or *gL*). Each column corresponds to a distinct frequency threshold at which haplotypes are called: 0.22% (left), 0.436% (middle), and 0.88% (right). Top panel: The number of minor UCD52 haplotypes called per sequenced sample, by tissue and week post-infection. Bottom panel: The dynamics of minor UCD52 haplotypes identified in more than one sample. Each dot represents a recurrent haplotype. Lines connecting dots indicate recurrent haplotypes in the same tissue. Haplotypes that recur in different tissues are not connected by lines. These figures together indicate that a subset of minor haplotypes persist over time and are shared between compartments. This result is robust across a large range of haplotype-calling thresholds (0.22–0.88%).
(PDF)
