# Supplementary figures and images for: Intrahost cytomegalovirus population genetics following antibody pretreatment in a monkey model of congenital transmission
Source: PLoS Pathog. 2020 Feb 14;16(2):e1007968. doi: 10.1371/journal.ppat.1007968 (PMC7046290; doi:10.1371/journal.ppat.1007968)

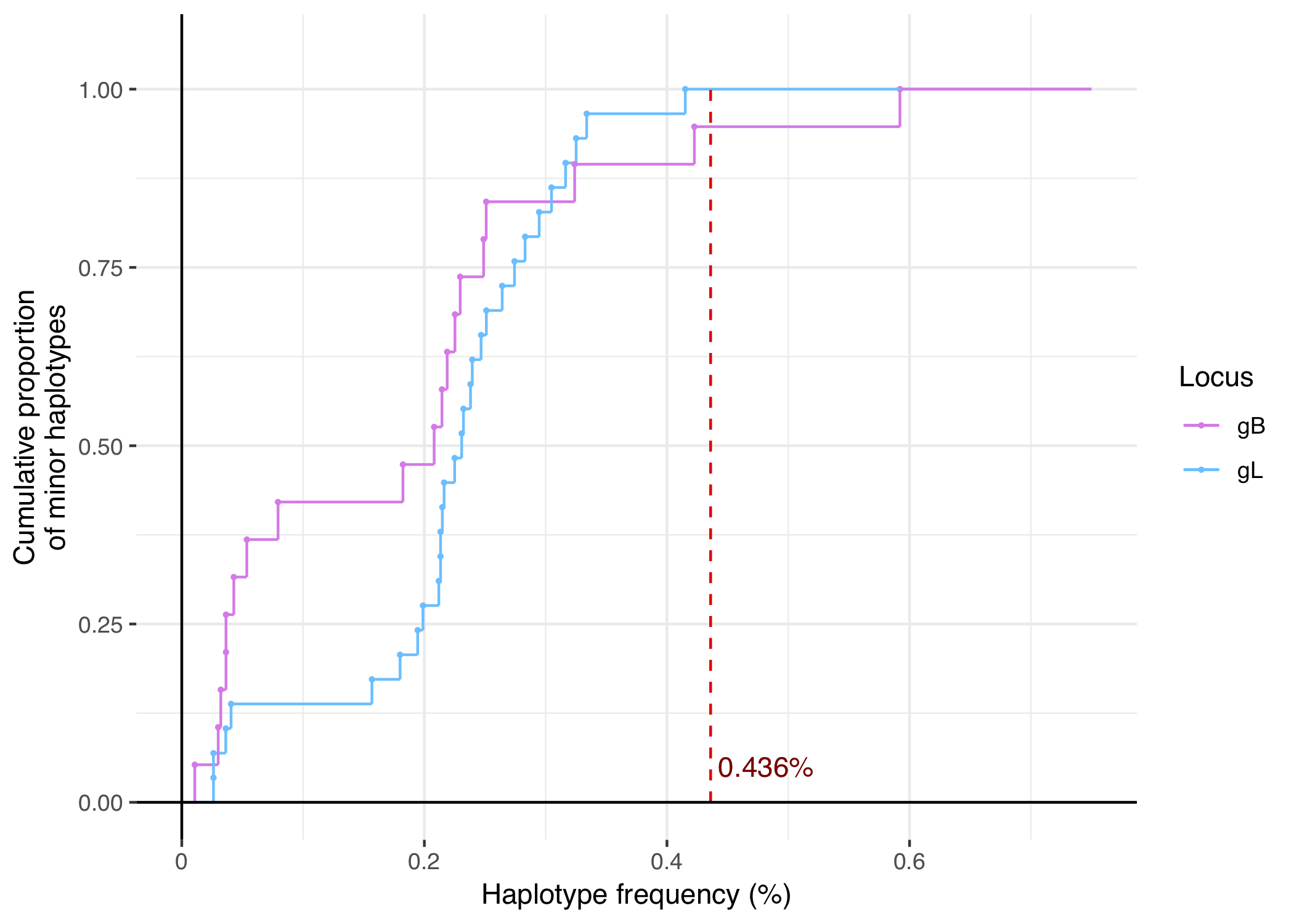

Supplement: S1 Fig — Minor haplotypes were identified from the synthetic plasmid control samples as described in the Methods section, for both the gB locus and the gL locus. The figure shows, for each locus, the fraction of identified minor haplotypes (y-axis) that fall at the haplotype frequency shown on the x-axis or below. The vertical red line shows the frequency threshold of 0.436% that was used to call minor haplotypes. (TIF) [file ppat.1007968.s001.tif]

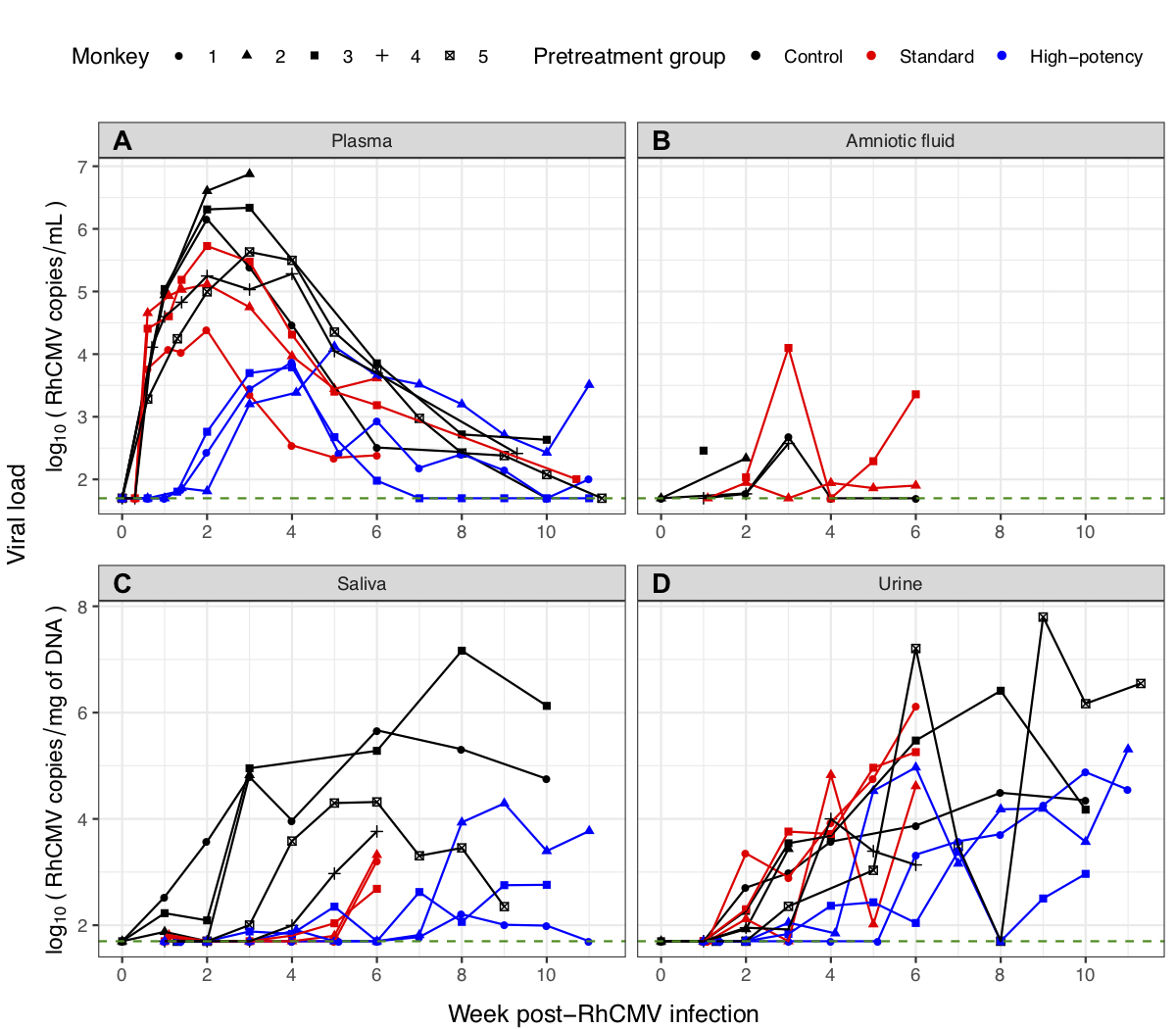

Supplement: S2 Fig — Virus was measured in (A) plasma, (B) amniotic fluid, (C) saliva, and (D) urine. Monkeys are color-coded according to pretreatment group: control (black), standard pretreatment group (red), and high-potency pretreatment group (blue). Monkey ID numbers correspond to those provided in S1 Table. Virus was detected in the plasma, saliva, and urine of all 11 monkeys. Virus was only detected in the amniotic fluid of the 5 control group monkeys and in 2 of the 3 standard HIG group monkeys. Viral load levels shown here are average values when more than one measurement was available (Methods). (TIF) [file ppat.1007968.s002.tif]

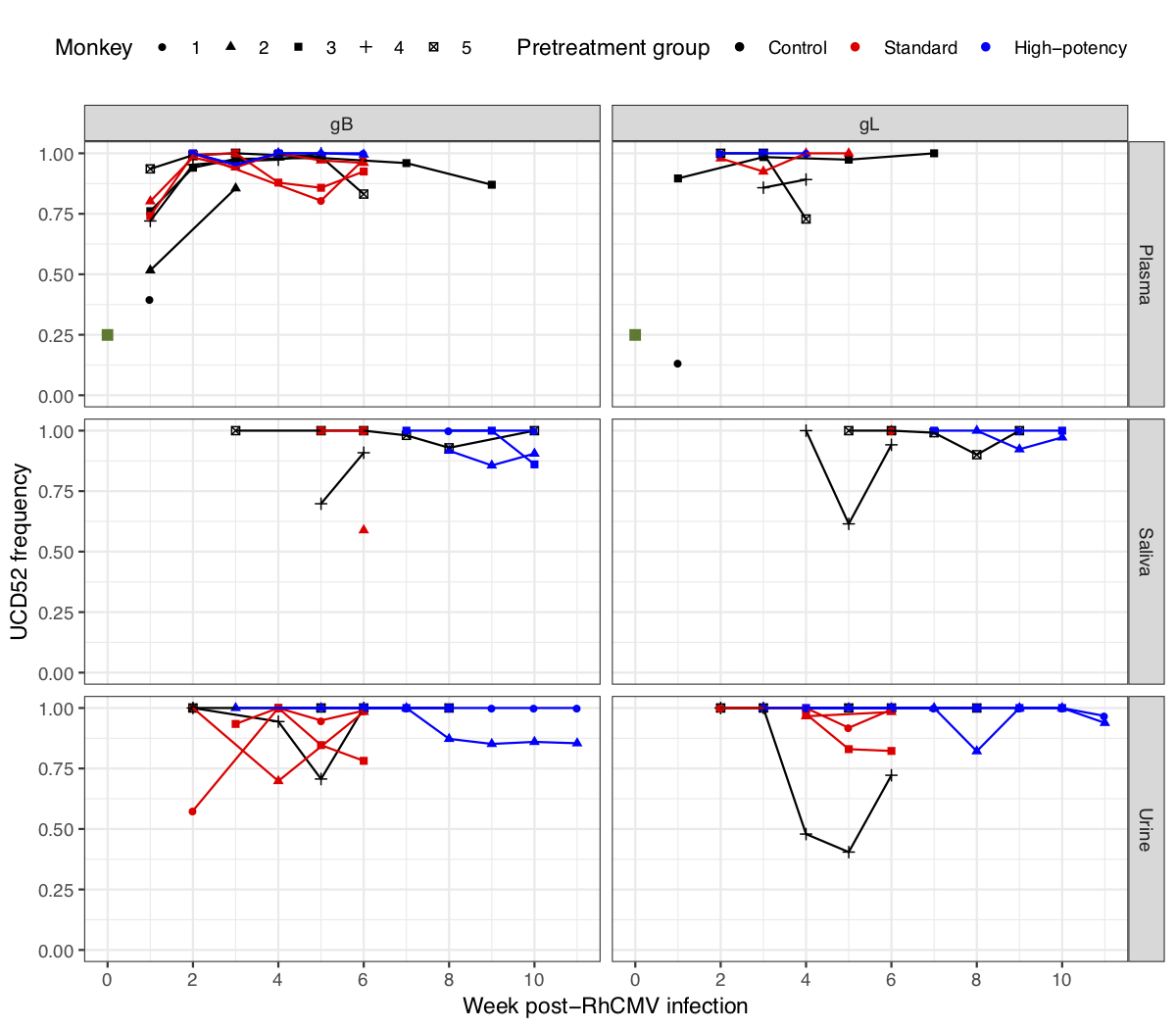

Supplement: S3 Fig — The proportion of the RhCMV population belonging to strain UCD52 is shown for maternal plasma, saliva, and urine. Strain frequencies were calculated for the gB locus (left column) and for the gL locus (right column). Green squares in the plasma subplots denote the fraction of the viral inoculum that was UCD52 (25%). (TIF) [file ppat.1007968.s003.tif]

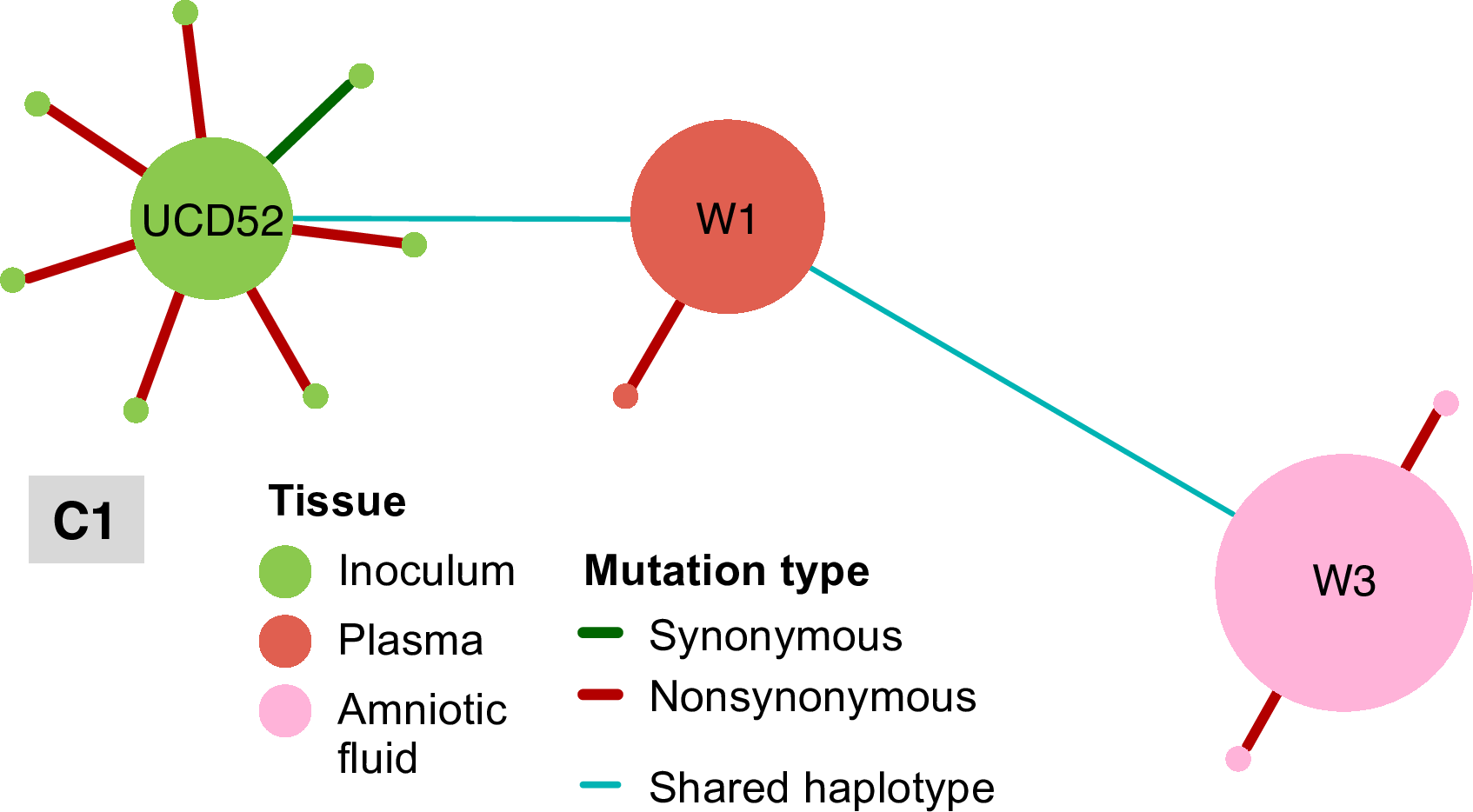

Supplement: S4 Fig — Colorcoding of nodes and edges are as in Fig 1, which show haplotype networks for C4, S2, and HP3. (TIF) [file ppat.1007968.s004.tif]

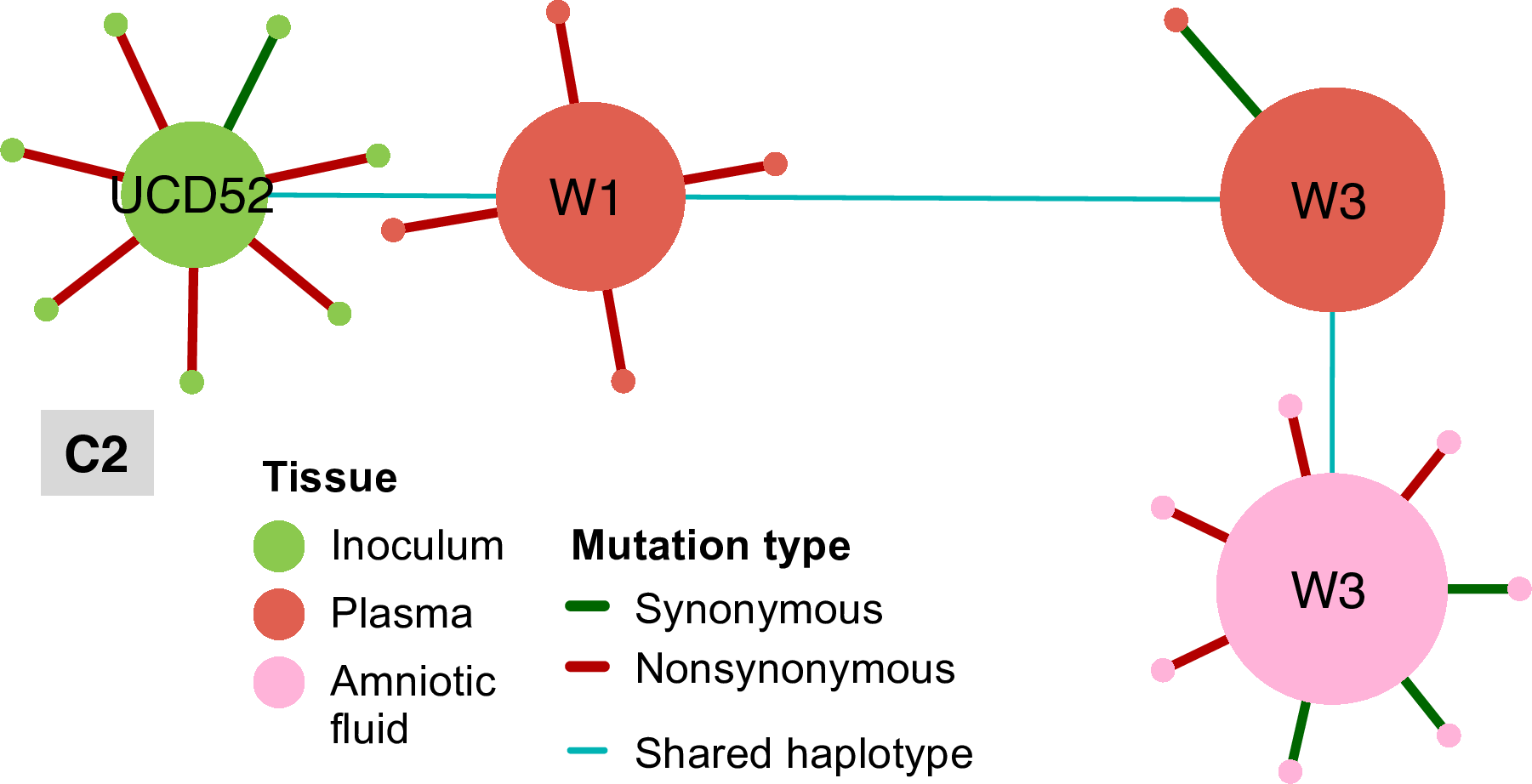

Supplement: S5 Fig — Colorcoding of nodes and edges are as in Fig 1, which show haplotype networks for C4, S2, and HP3. (TIF) [file ppat.1007968.s005.tif]

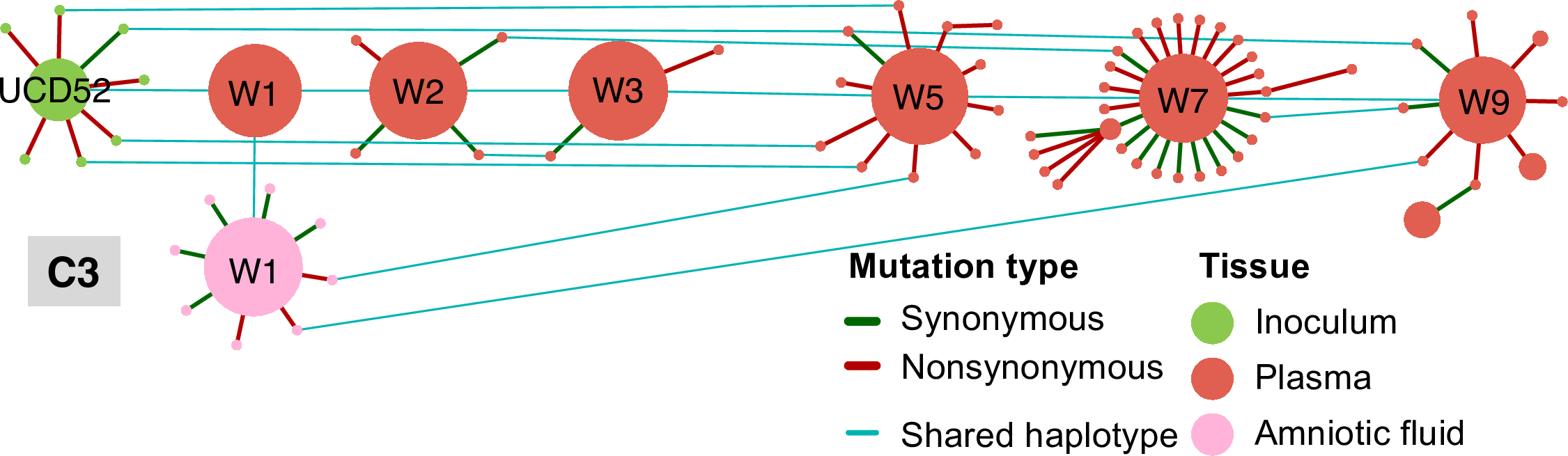

Supplement: S6 Fig — Colorcoding of nodes and edges are as in Fig 1, which show haplotype networks for C4, S2, and HP3. (TIF) [file ppat.1007968.s006.tif]

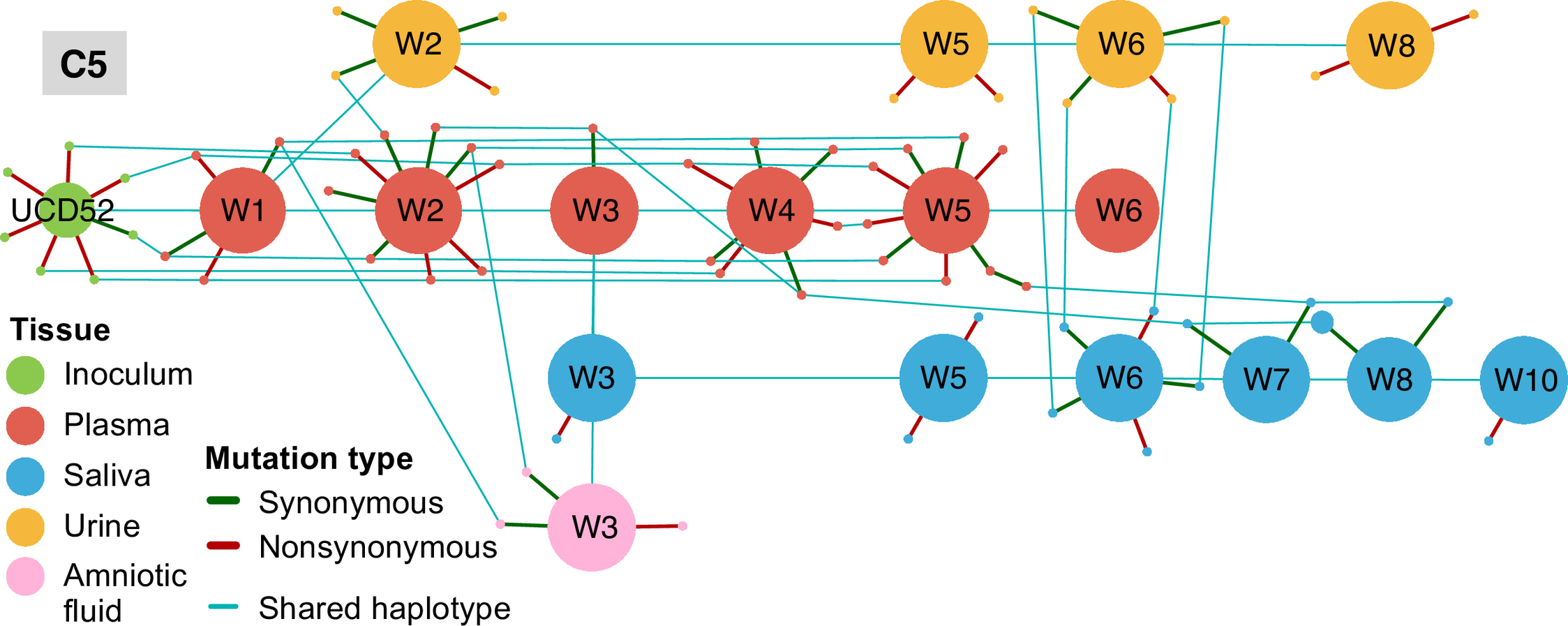

Supplement: S7 Fig — Colorcoding of nodes and edges are as in Fig 1, which show haplotype networks for C4, S2, and HP3. (TIF) [file ppat.1007968.s007.tif]

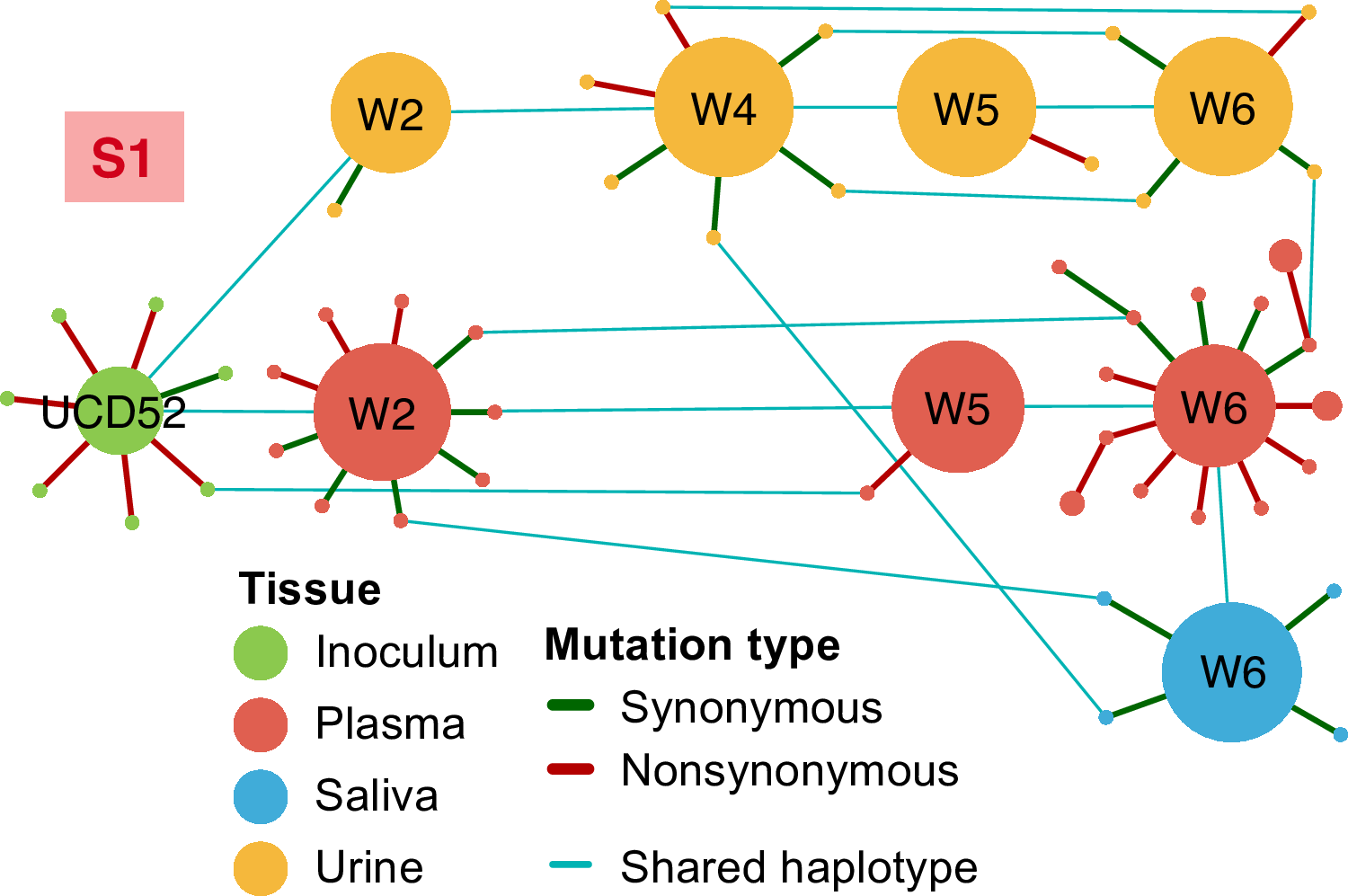

Supplement: S8 Fig — Colorcoding of nodes and edges are as in Fig 1, which show haplotype networks for C4, S2, and HP3. (TIF) [file ppat.1007968.s008.tif]

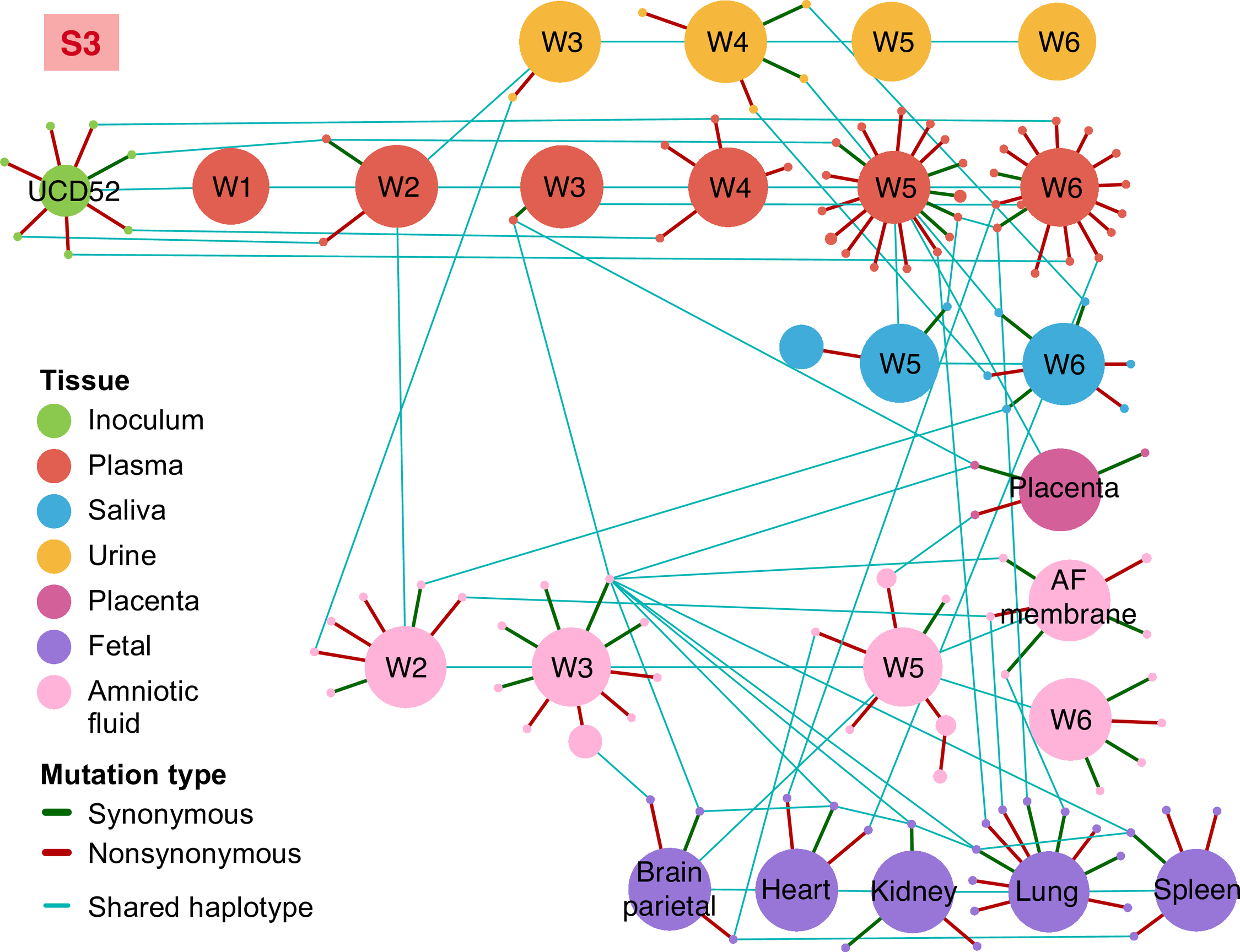

Supplement: S9 Fig — Colorcoding of nodes and edges are as in Fig 1, which show haplotype networks for C4, S2, and HP3. (TIF) [file ppat.1007968.s009.tif]

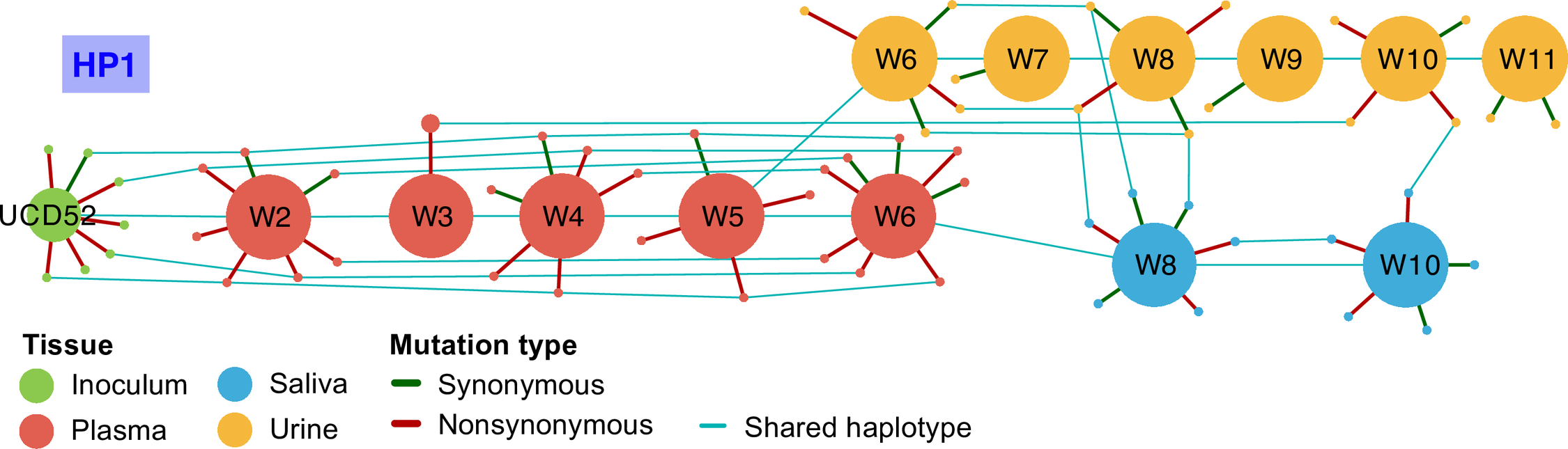

Supplement: S10 Fig — Colorcoding of nodes and edges are as in Fig 1, which show haplotype networks for C4, S2, and HP3. (TIF) [file ppat.1007968.s010.tif]

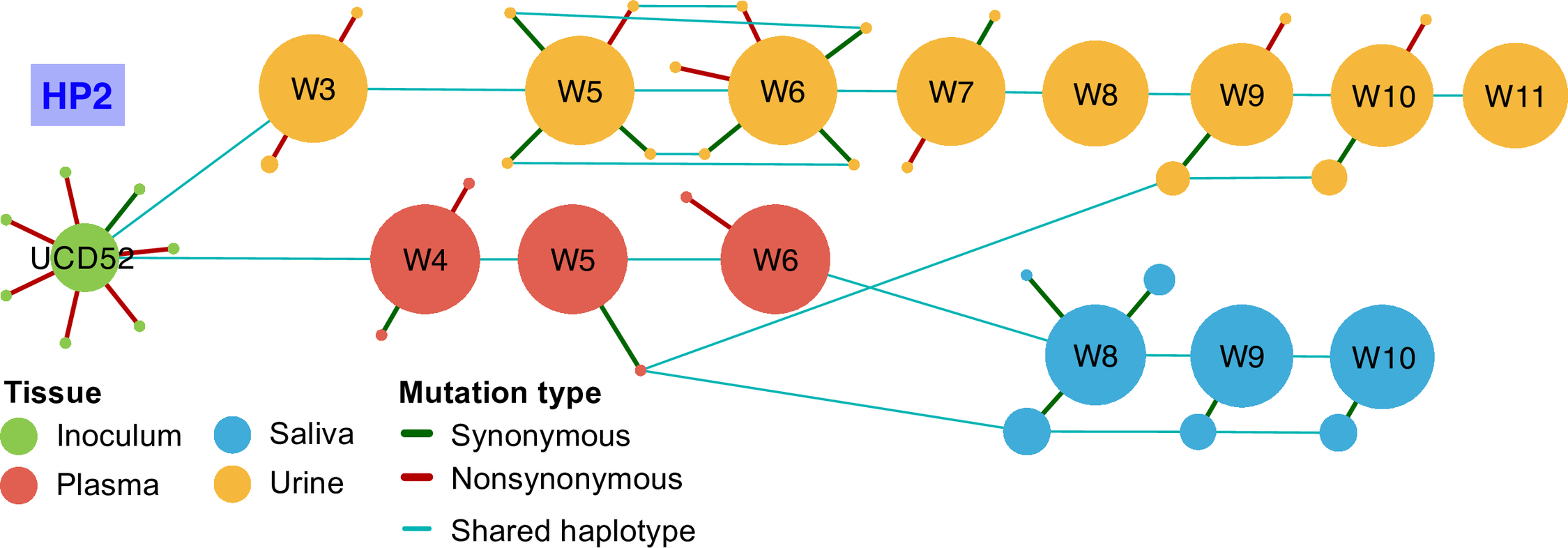

Supplement: S11 Fig — Colorcoding of nodes and edges are as in Fig 1, which show haplotype networks for C4, S2, and HP3. (TIF) [file ppat.1007968.s011.tif]

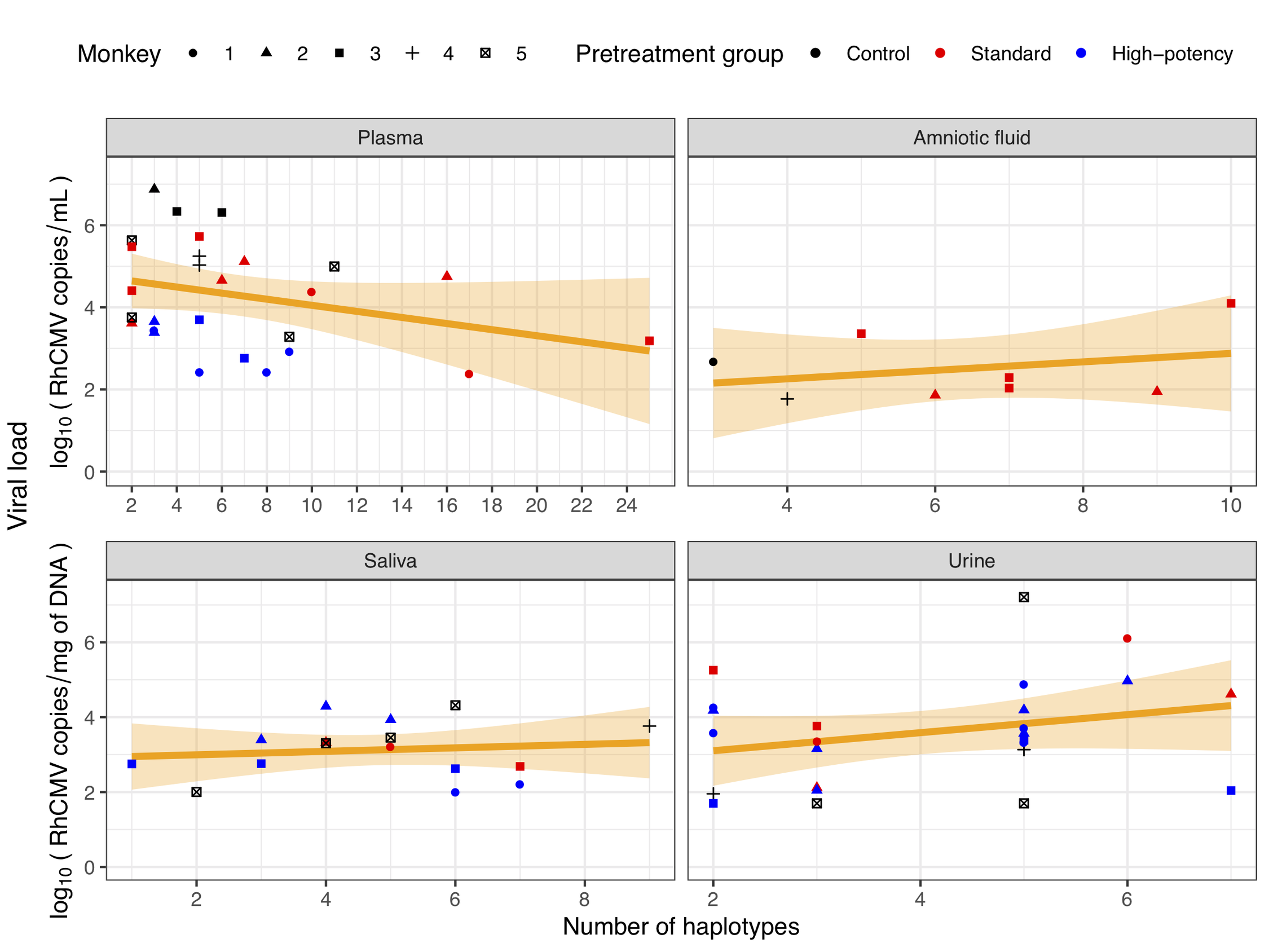

Supplement: S12 Fig — The correlation between viral load and the number of gB haplotypes was not significantly positive for any of the four analyzed compartments (plasma, amniotic fluid, saliva, urine). (TIF) [file ppat.1007968.s012.tif]

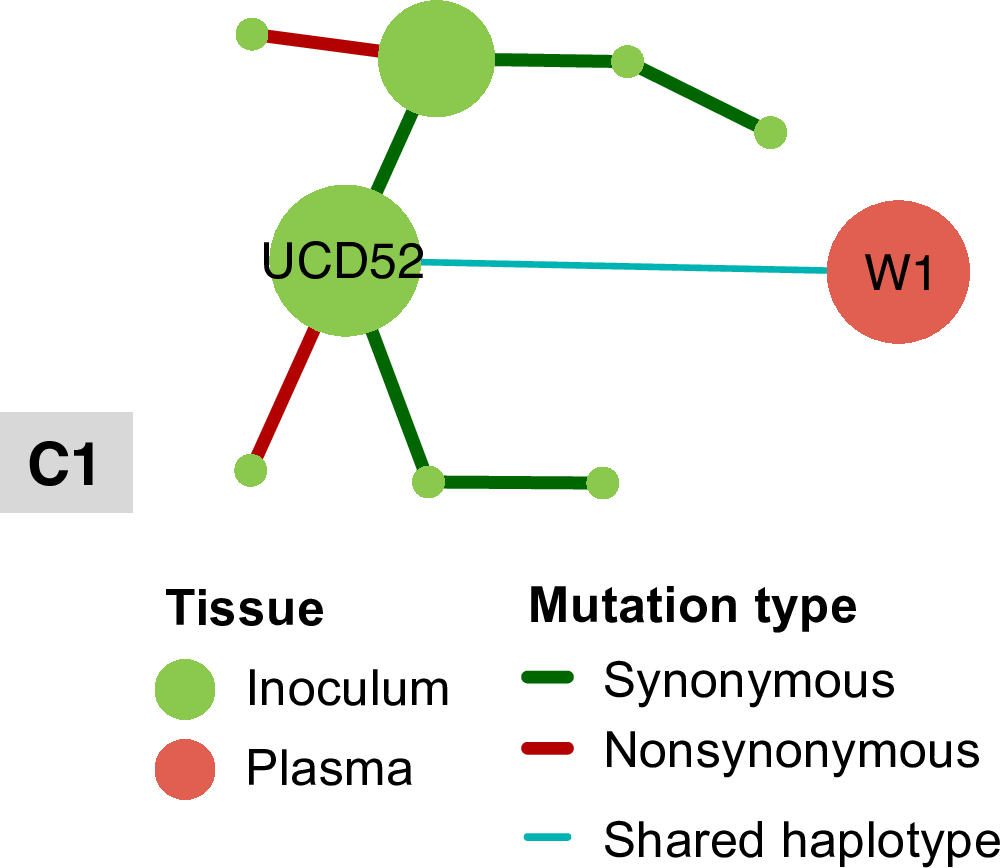

Supplement: S13 Fig — Colorcoding of nodes and edges are as in Fig 1. (TIF) [file ppat.1007968.s013.tif]

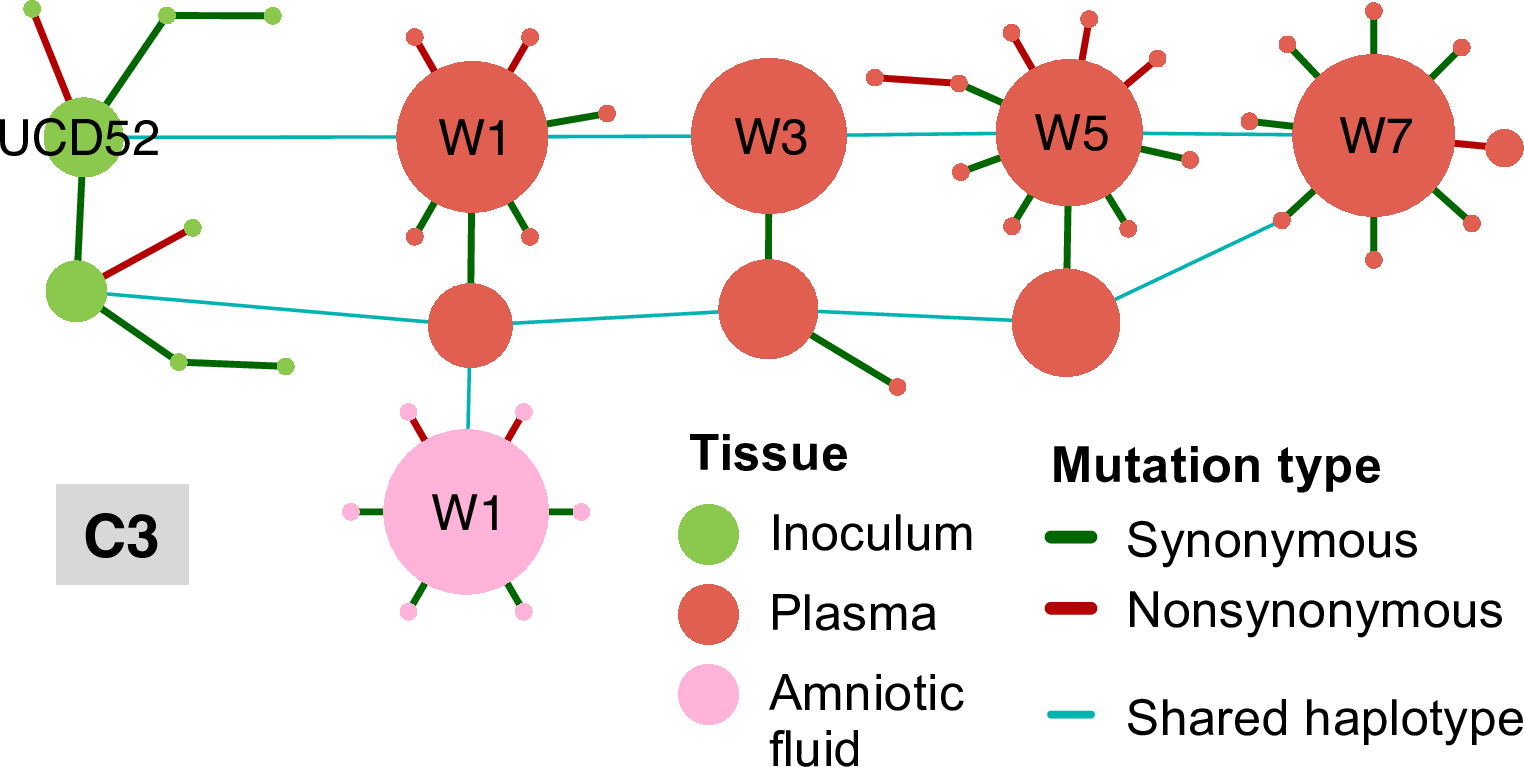

Supplement: S14 Fig — Colorcoding of nodes and edges are as in Fig 1. (TIF) [file ppat.1007968.s014.tif]

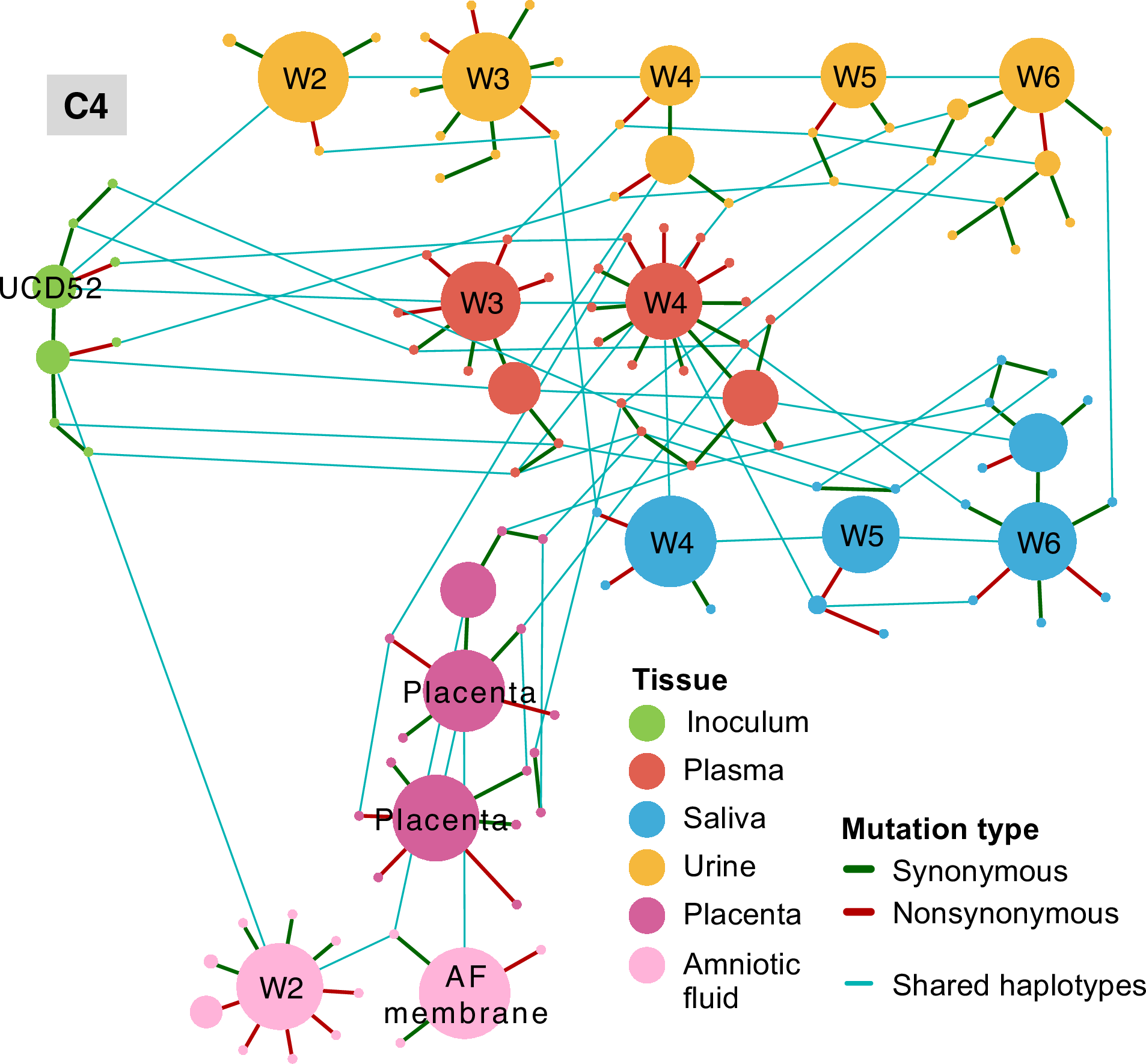

Supplement: S15 Fig — Colorcoding of nodes and edges are as in Fig 1. (TIF) [file ppat.1007968.s015.tif]

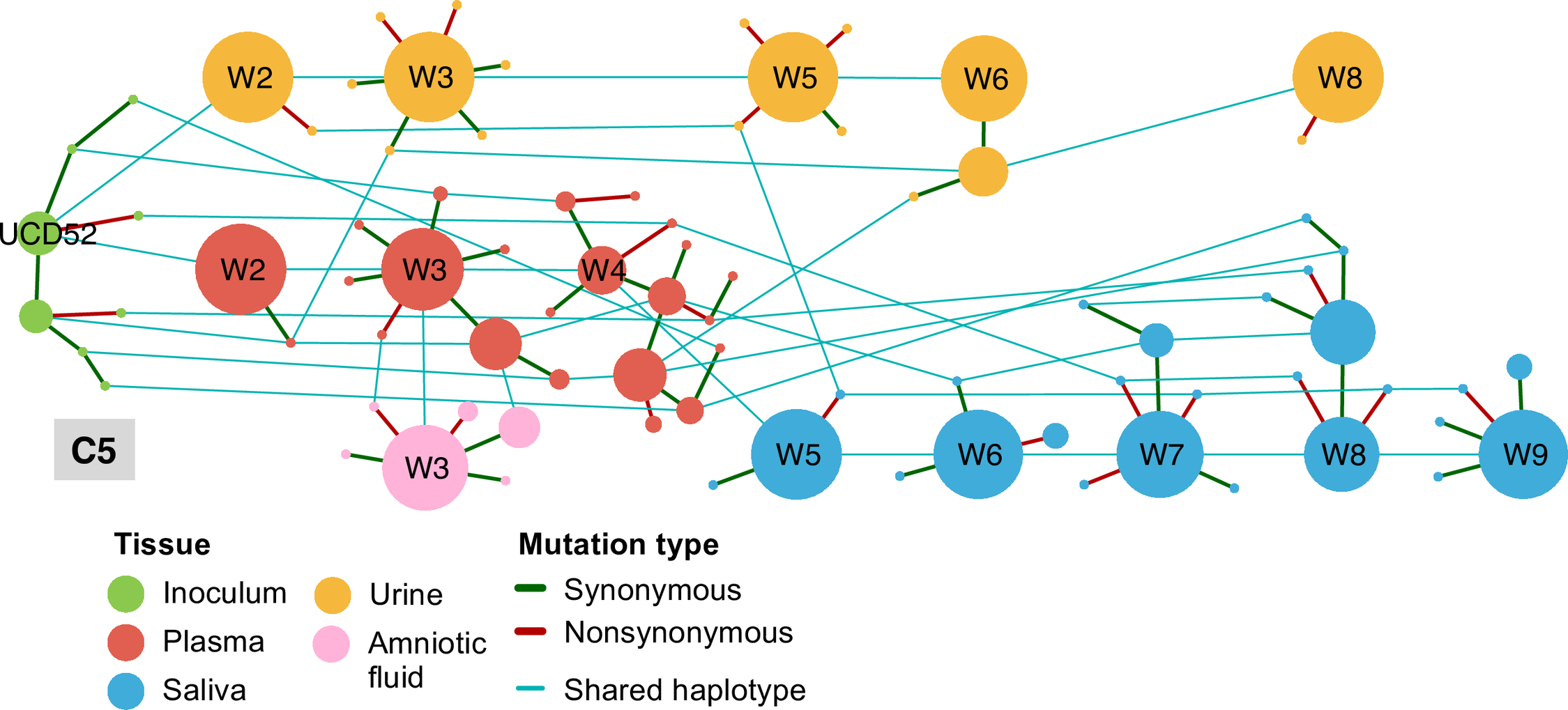

Supplement: S16 Fig — Colorcoding of nodes and edges are as in Fig 1. (TIF) [file ppat.1007968.s016.tif]

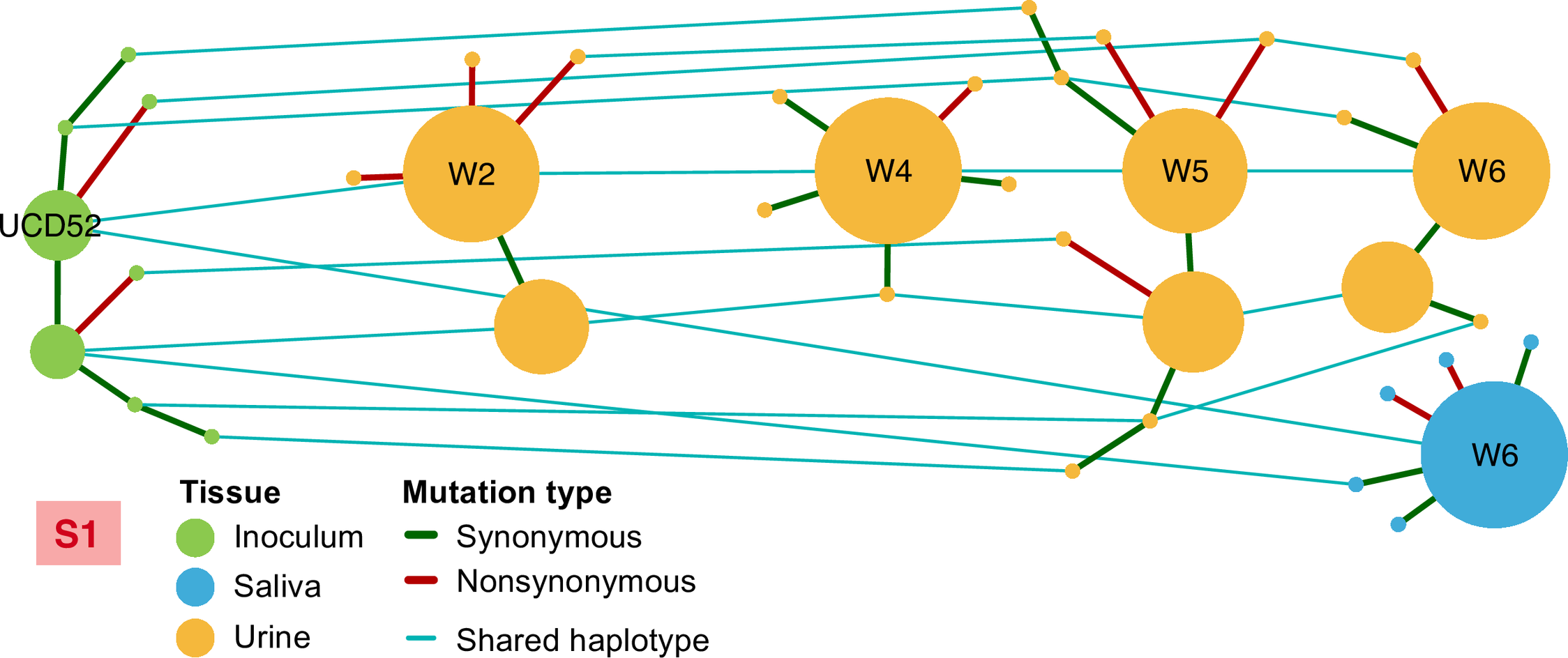

Supplement: S17 Fig — Colorcoding of nodes and edges are as in Fig 1. (TIF) [file ppat.1007968.s017.tif]

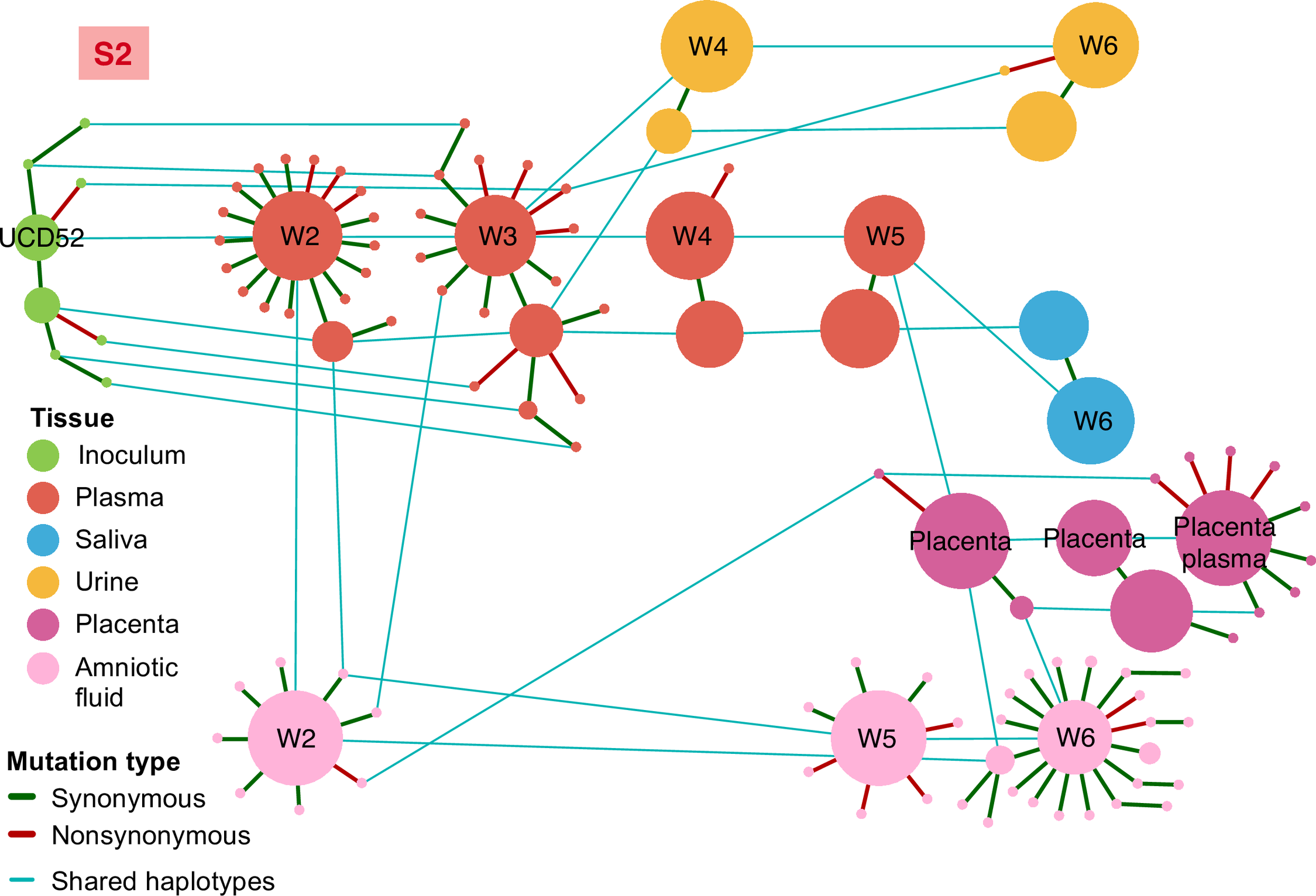

Supplement: S18 Fig — Colorcoding of nodes and edges are as in Fig 1. (TIF) [file ppat.1007968.s018.tif]

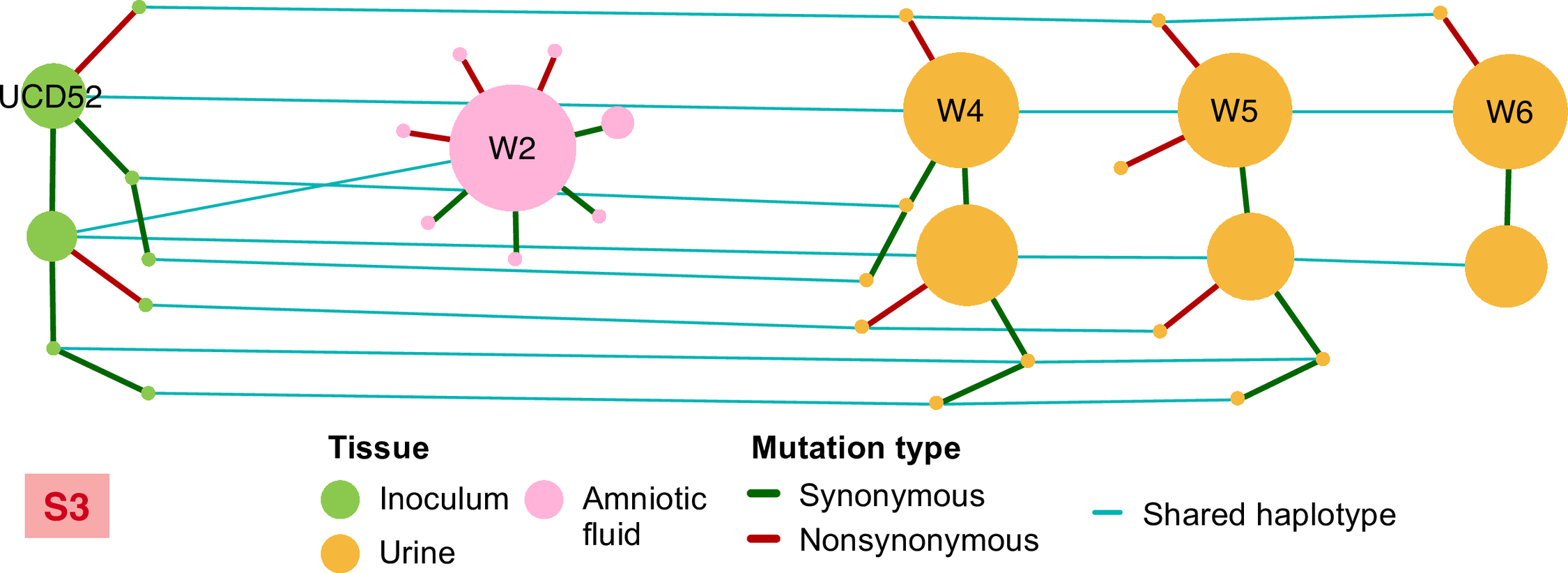

Supplement: S19 Fig — Colorcoding of nodes and edges are as in Fig 1. (TIF) [file ppat.1007968.s019.tif]

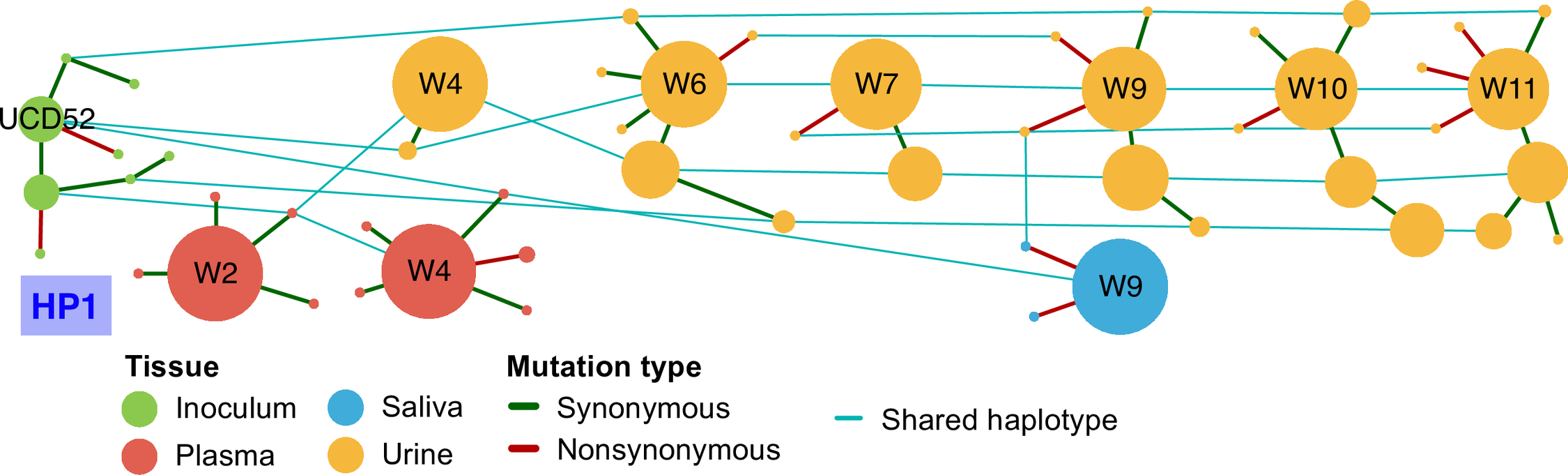

Supplement: S20 Fig — Colorcoding of nodes and edges are as in Fig 1. (TIF) [file ppat.1007968.s020.tif]

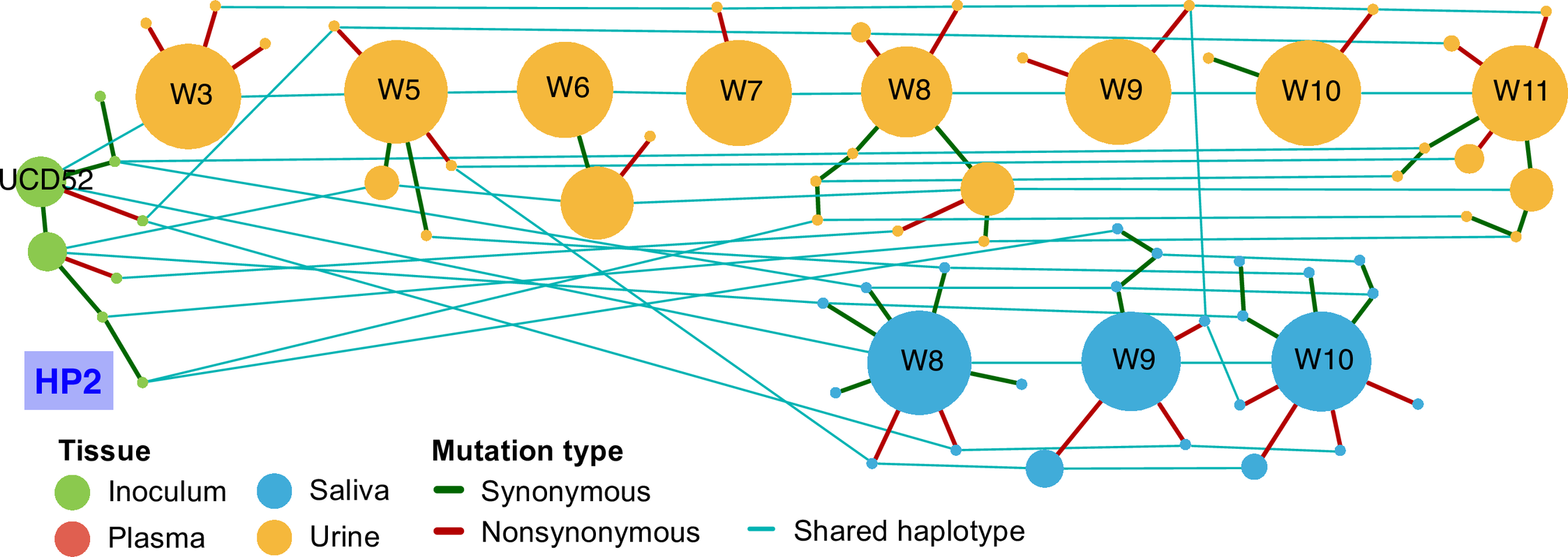

Supplement: S21 Fig — Colorcoding of nodes and edges are as in Fig 1. (TIF) [file ppat.1007968.s021.tif]

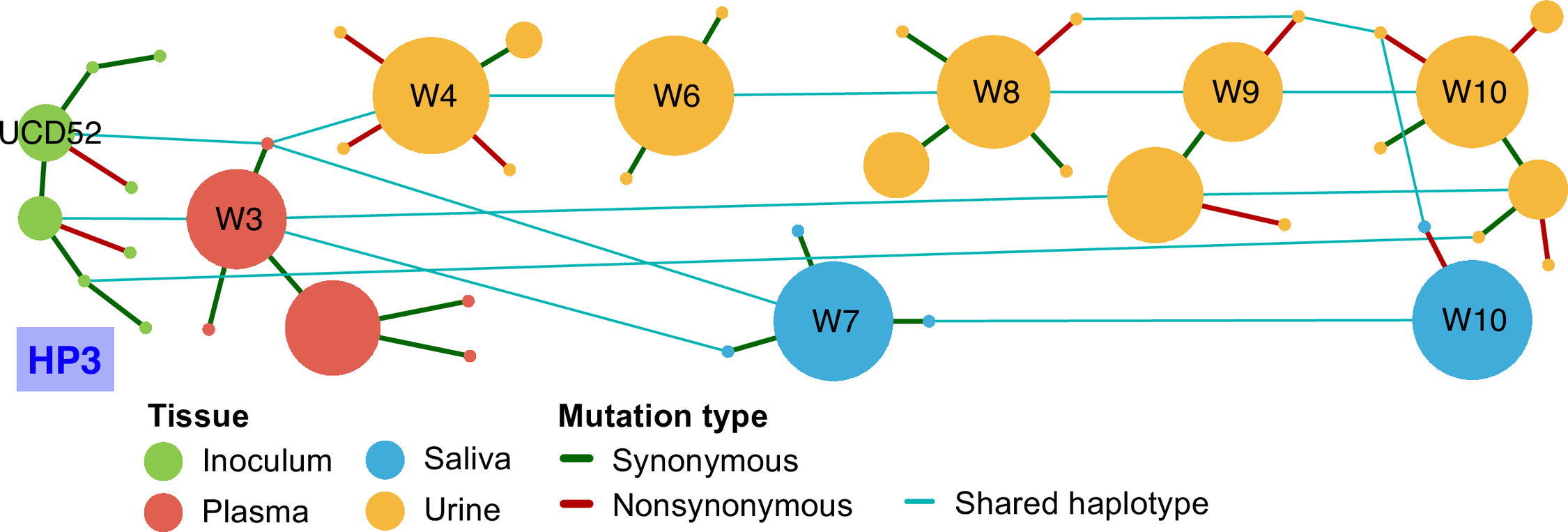

Supplement: S22 Fig — Colorcoding of nodes and edges are as in Fig 1. (TIF) [file ppat.1007968.s022.tif]

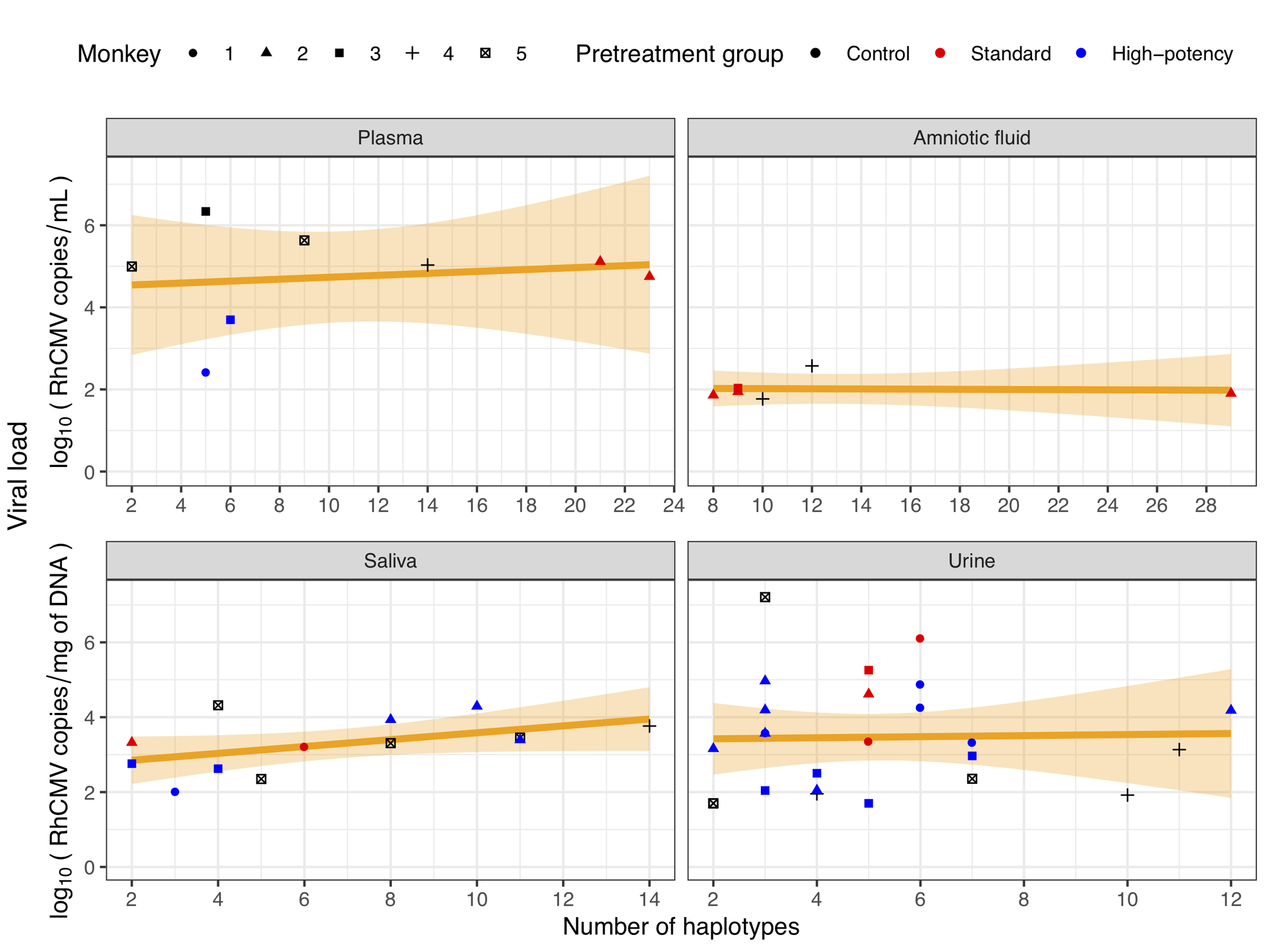

Supplement: S23 Fig — The correlation between viral load and the number of gL haplotypes was not significantly positive for any of the four analyzed compartments (plasma, amniotic fluid, saliva, urine). (TIF) [file ppat.1007968.s023.tif]

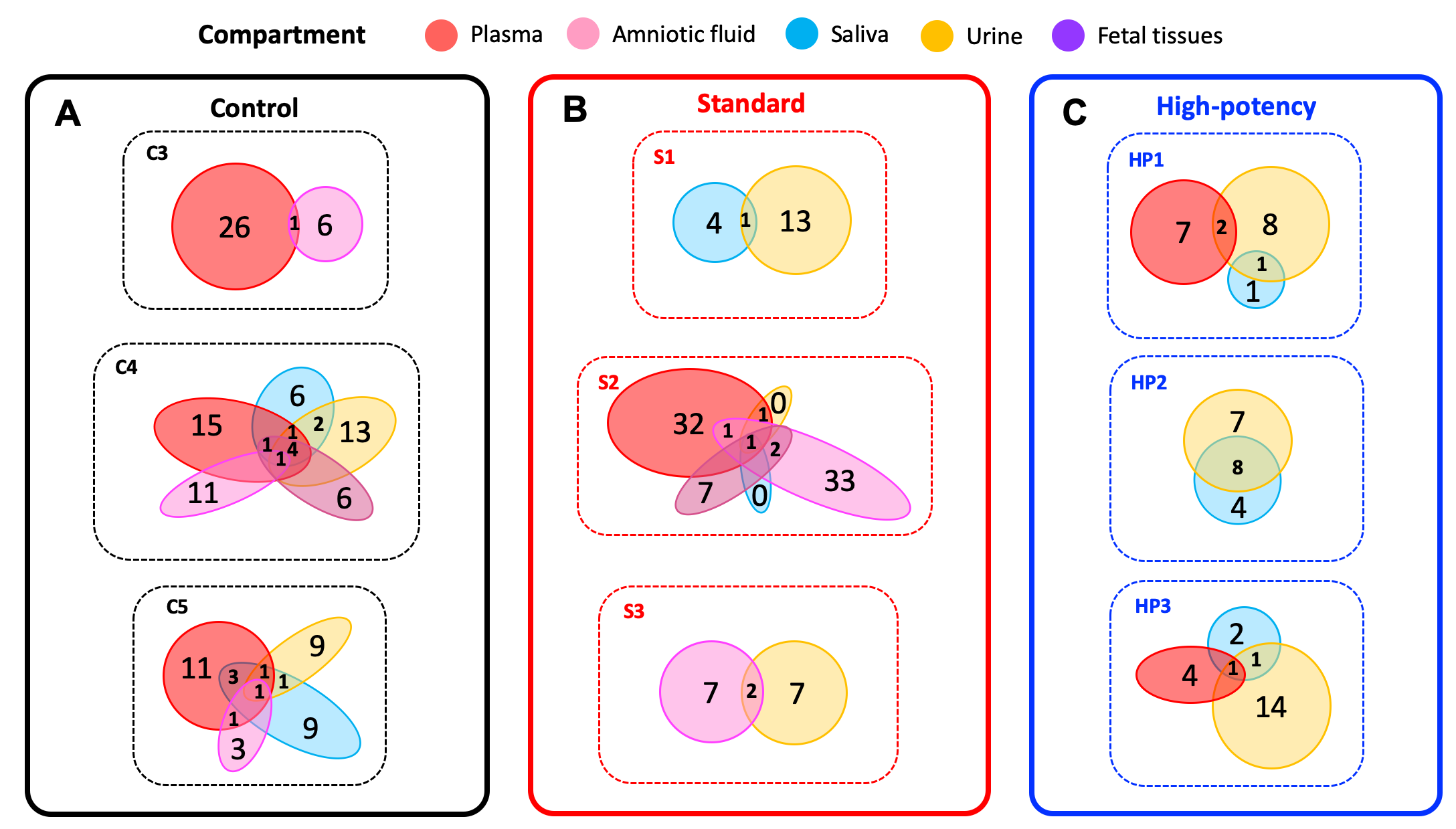

Supplement: S24 Fig — Here, the set of minor haplotypes for a given compartment includes all timepoint samples from that compartment. Patterns of minor haplotype sharing for (A) control group monkeys, (B) standard pretreatment group monkeys, and (C) high-potency pretreatment group monkeys. Compartments are colorcoded as in Fig 1. (TIF) [file ppat.1007968.s024.tif]

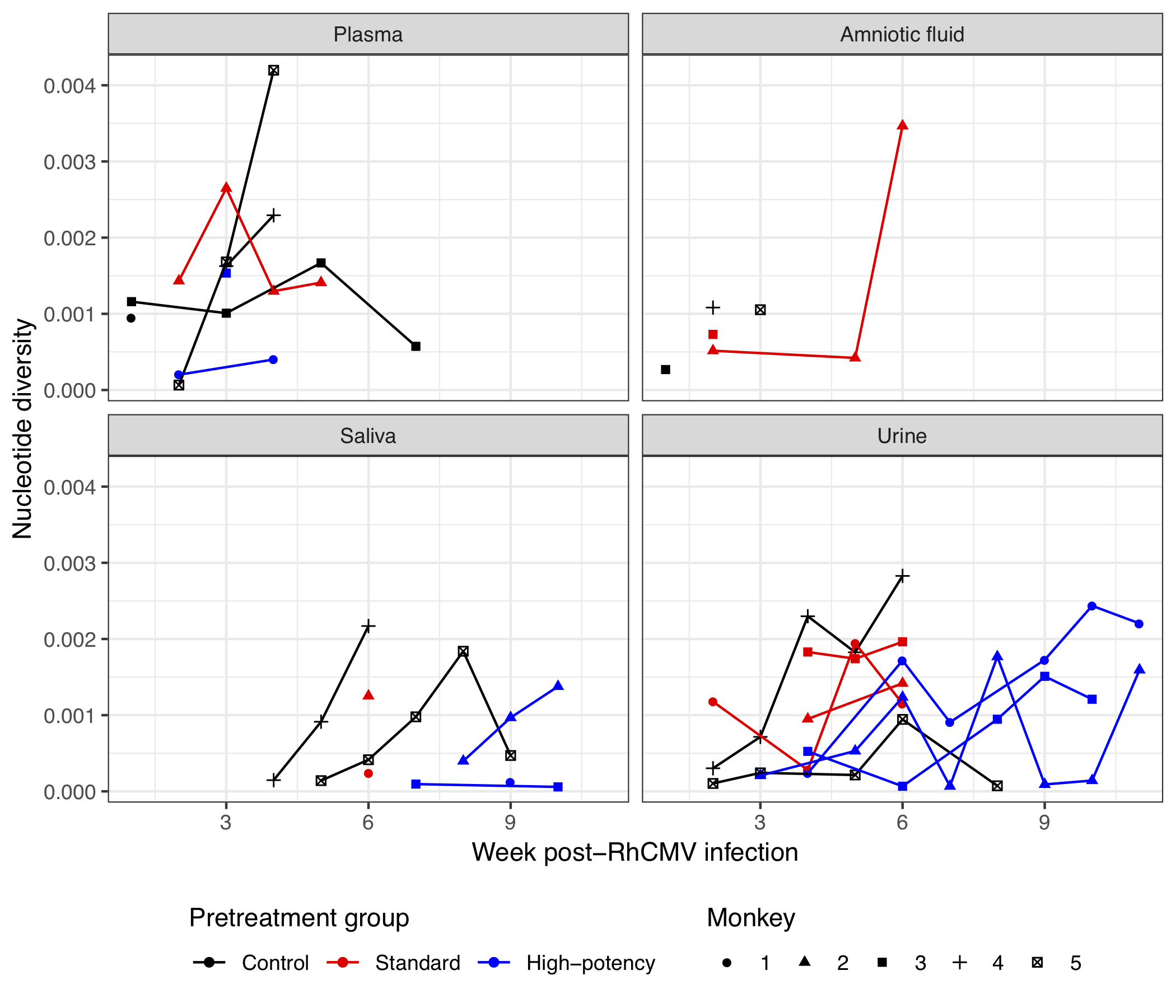

Supplement: S25 Fig — Marker symbols correspond with those in S2 Fig. (TIF) [file ppat.1007968.s025.tif]
