# Supplementary material for: Intrahost cytomegalovirus population genetics following antibody pretreatment in a monkey model of congenital transmission
Source: PLoS Pathog. 2020 Feb 14;16(2):e1007968. doi: 10.1371/journal.ppat.1007968 (PMC7046290; doi:10.1371/journal.ppat.1007968)
Supplement: S1 Table — Dams are separated by pretreatment group: control (C1-C5), standard (S1-S3), and high-potency (HP1-HP3). In addition to the C1-C5, S1-S3, and HP1-HP3 identifiers, individual monkeys are identified according to names previously used in [9] and [27]. Cells are colored according to the legend provided. Text in the white-colored cells indicate which loci were successfully sequenced and included in our analyses (gB = glycoprotein B region; gL = glycoprotein L region). Numbers in the cells, when present, indicate the number of sample replicates that were available for analysis, when not two. A single round of PCR was used for all plasma samples. Nested PCR was used for all other samples. (PDF) [file ppat.1007968.s026.pdf]

Pretreatment group	Monkey	Tissue	Week post-RhCMV infection										
			1	2	3	4	5	6	7	8	9	10	11
Control	C1 (145-97)	Plasma	gB(1),gL(4)	neither				neither					
		Amn. fluid			gB								
	C2 (174-97)	Plasma	gB(1)	neither	gB(4)								
		Amn. fluid			gB								
	C3 (369-09)	Plasma	gB,gL	gB	gB,gL		gB,gL		gB(3),gL		gB		neither
		Amn. fluid	gB,gL										
	C4 (GM04)	Plasma	gB	gB	gB,gL	gB,gL	gB(1)						
		Amn. fluid		gB,gL	gL								
		Saliva				gL(1)	gB,gL	gB,gL					
		Urine		gB,gL	gL	gB,gL	gB,gL	gB,gL					
	C5 (HD79)	Plasma	gB	gB,gL	gB,gL	gB,gL	gB	gB					
		Amn. fluid			gB(3),gL								
		Saliva			gB		gB,gL	gB,gL	gB,gL	gB,gL	gL	gB	
		Urine		gB,gL	gL		gB,gL	gB,gL		gB,gL			
Standard	S1 (JC65)	Plasma	neither	gB	neither	gB	gB	gB(3)					
		Saliva						gB(4),gL(4)					
		Urine		gB,gL		gB,gL	gB,gL	gB,gL					
	S2 (GI73)	Plasma	gB	gB(4),gL(1)	gB(3),gL(1)	gB,gL	gB,gL	gB					
		Amn. fluid		gB,gL		neither	gB,gL	gL					
		Saliva						gB,gL					
		Urine		gB		gB,gL		gB,gL					
	S3 (IM67)	Plasma	gB	gB(4)	gB(4)	gB	gB	gB					
		Amn. fluid		gB,gL	gB	neither	gB	gB					
		Saliva				neither	gB	gB					
		Urine		neither	gB	gB,gL	gB,gL	gB,gL					
High-potency	HP1 (JM52)	Plasma		gB,gL	gB(3)	gB,gL	gB	gB					
		Saliva								gB	gL	gB	
		Urine				gL		gB,gL	gB,gL	gB	gB,gL	gB,gL	gB,gL
	HP2 (HR73)	Plasma		neither	neither	gB	gB	gB					
		Saliva				neither				gB,gL	gB,gL	gB,gL	neither
		Urine			gB,gL		gB,gL	gB,gL	gB,gL	gB,gL	gB,gL	gB,gL	gB,gL
	HP3 (HD82)	Plasma		gB	gB,gL	gB	neither	neither					
		Saliva					neither		gB,gL		gB	gB,gL	
		Urine				gB,gL	neither	gB,gL	gB	gB,gL	gL	gL	

Sample not taken
Sample depleted
Sample not sequenced due to low viral load
Failed PCR amplification
