# Supplementary material for: Intrahost cytomegalovirus population genetics following antibody pretreatment in a monkey model of congenital transmission
Source: PLoS Pathog. 2020 Feb 14;16(2):e1007968. doi: 10.1371/journal.ppat.1007968 (PMC7046290; doi:10.1371/journal.ppat.1007968)
Supplement: S2 Table — All listed samples had two successfully sequenced replicates and underwent nested PCR. (PDF) [file ppat.1007968.s027.pdf]

Pretreatment group	Monkey	Tissue	Week	Samples
Control	C4	Amn. fluid membrane	3	gB, gL
		Placenta -1	3	gB, gL
		Placenta -2	3	gB, gL
Standard	S2	Placenta - 1	6	gB, gL
		Placenta - 2	6	gB, gL
		Placenta plasma	6	gB, gL
	S3	Amn. fluid membrane	6	gB
		Placenta - 2	6	gB
		Lung	6	gB
		Brain - parietal	6	gB
		Spleen	6	gB
		Heart	6	gB
		Kidney	6	gB
