# Supplementary material for: Intrahost cytomegalovirus population genetics following antibody pretreatment in a monkey model of congenital transmission
Source: PLoS Pathog. 2020 Feb 14;16(2):e1007968. doi: 10.1371/journal.ppat.1007968 (PMC7046290; doi:10.1371/journal.ppat.1007968)
Supplement: S5 Appendix — Each page shows the number and recurrence dynamics of minor UCD52 haplotypes in a single dam (or dam-fetus pair) at a single locus (gB or gL). Each column corresponds to a distinct frequency threshold at which haplotypes are called: 0.22% (left), 0.436% (middle), and 0.88% (right). Top panel: The number of minor UCD52 haplotypes called per sequenced sample, by tissue and week post-infection. Bottom panel: The dynamics of minor UCD52 haplotypes identified in more than one sample. Each dot represents a recurrent haplotype. Lines connecting dots indicate recurrent haplotypes in the same tissue. Haplotypes that recur in different tissues are not connected by lines. These figures together indicate that a subset of minor haplotypes persist over time and are shared between compartments. This result is robust across a large range of haplotype-calling thresholds (0.22–0.88%). (PDF) [file ppat.1007968.s032.pdf]

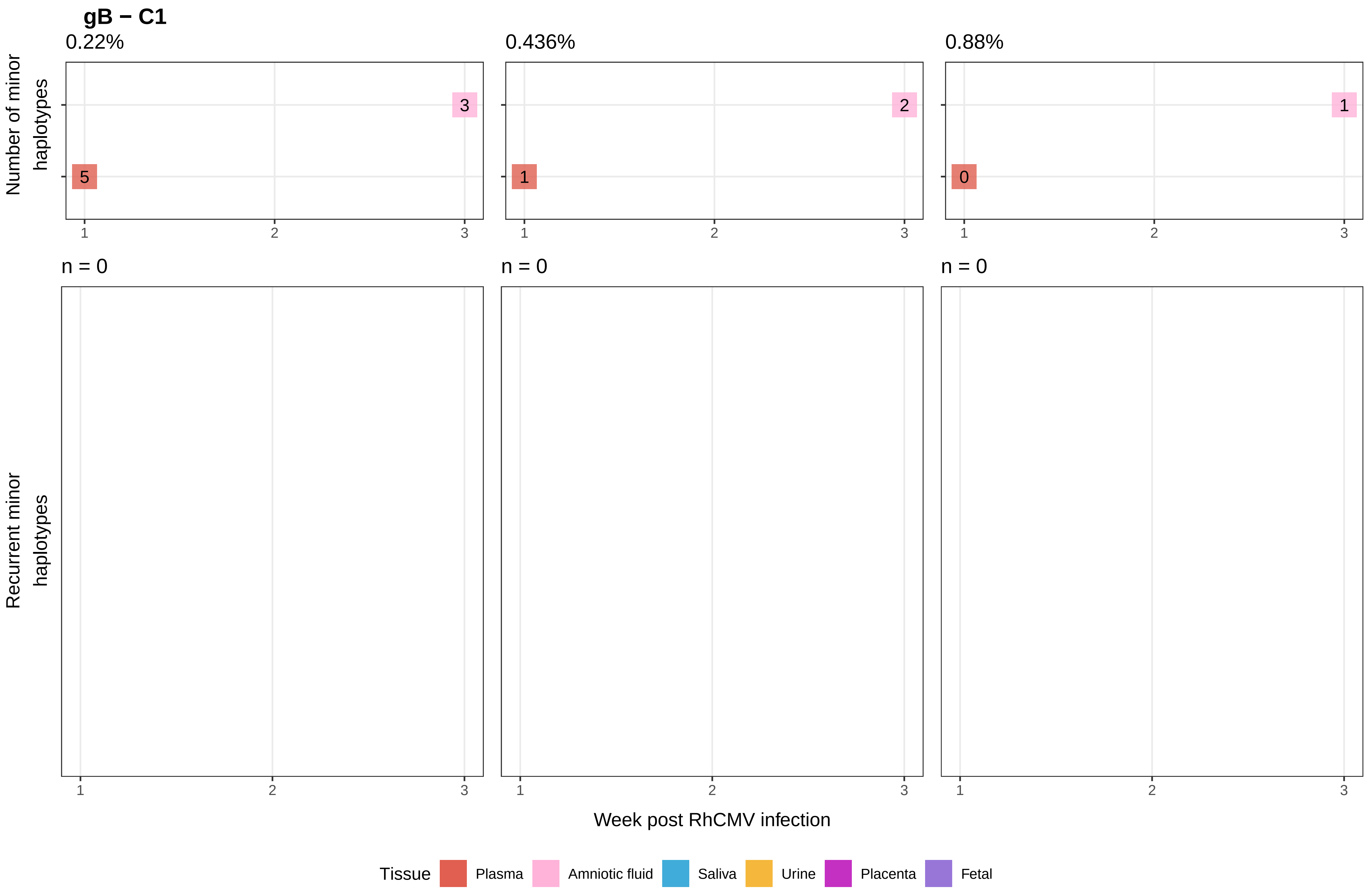
gB − C1
0.22%
0.436%
0.88%
Number of minor haplotypes
3
5
2
1
1
0
n = 0
n = 0
n = 0
Recurrent minor haplotypes
1
2
3
Week post RhCMV infection
Tissue
Plasma
Amniotic fluid
Saliva
Urine
Placenta
Fetal

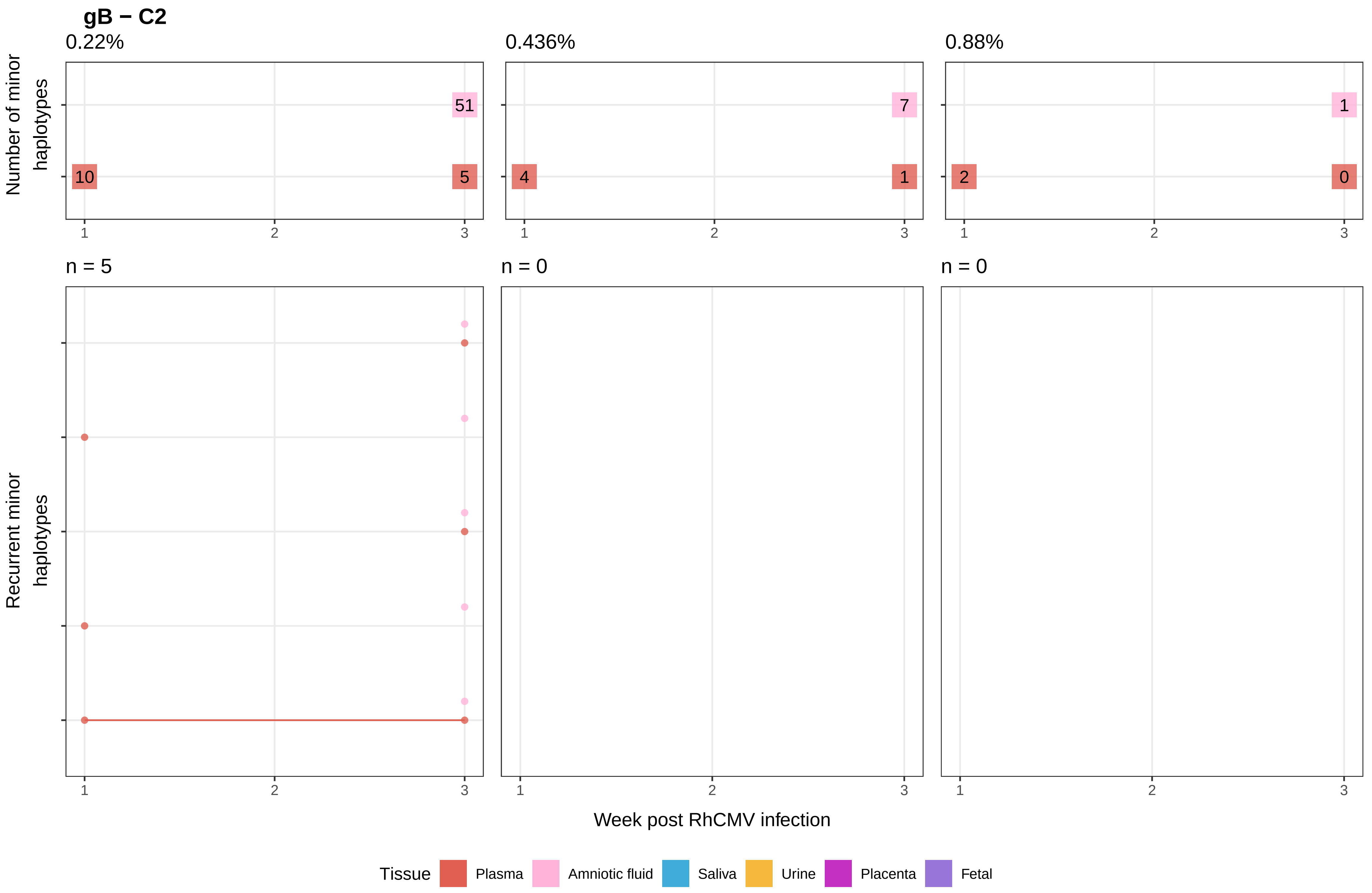

gB − C2
0.22%
0.436%
0.88%
Number of minor haplotypes
51
10
5
7
4
1
1
2
0
n = 5
n = 0
n = 0
Recurrent minor haplotypes
1
2
3
Week post RhCMV infection
Tissue
Plasma
Amniotic fluid
Saliva
Urine
Placenta
Fetal

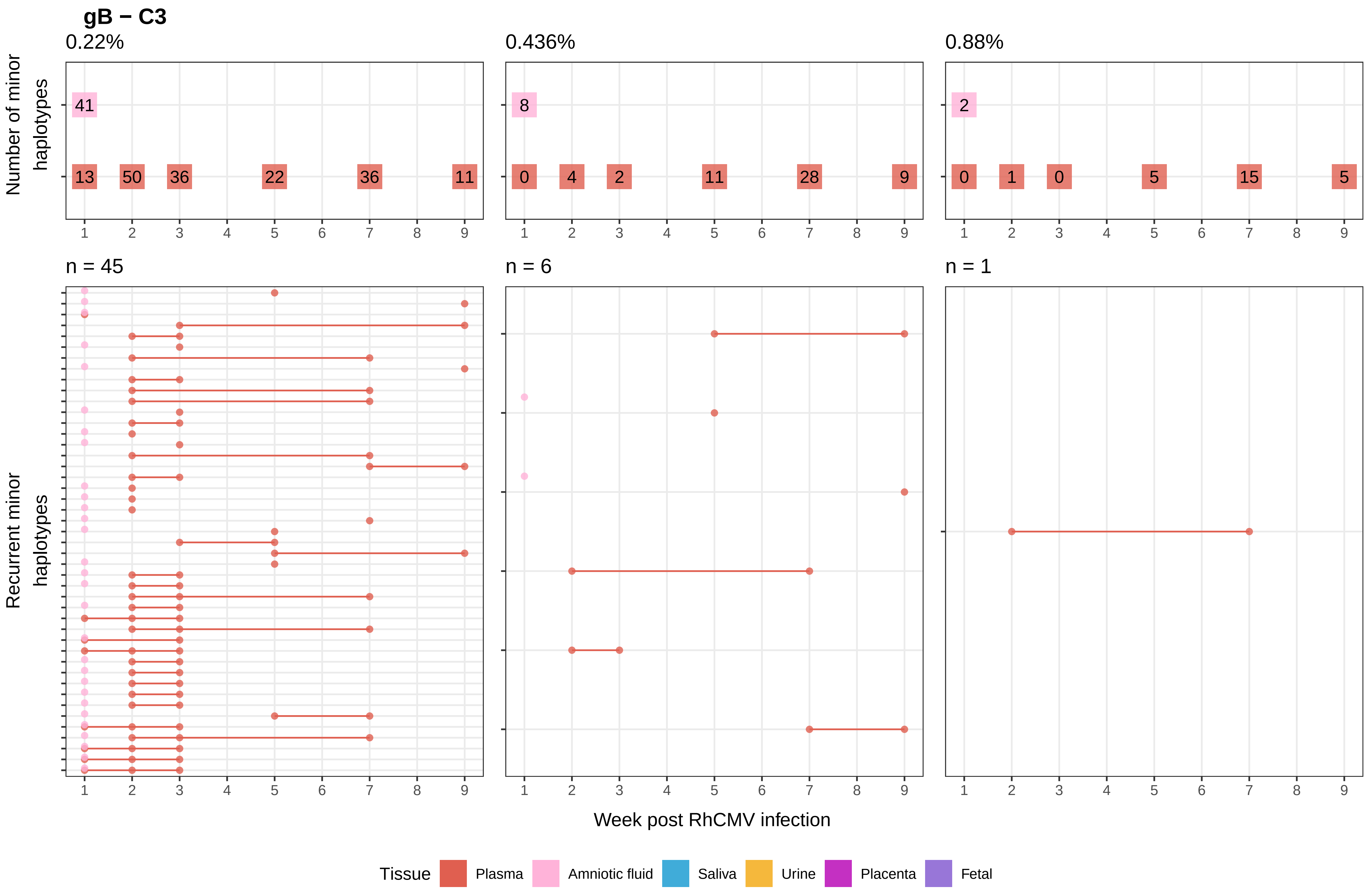

gB − C3
0.22%
0.436%
0.88%
Number of minor haplotypes
41
13
50
36
22
36
11
8
0
4
2
11
28
9
2
0
1
0
5
15
5
n = 45
n = 6
n = 1
Recurrent minor haplotypes
Week post RhCMV infection
Tissue
Plasma
Amniotic fluid
Saliva
Urine
Placenta
Fetal

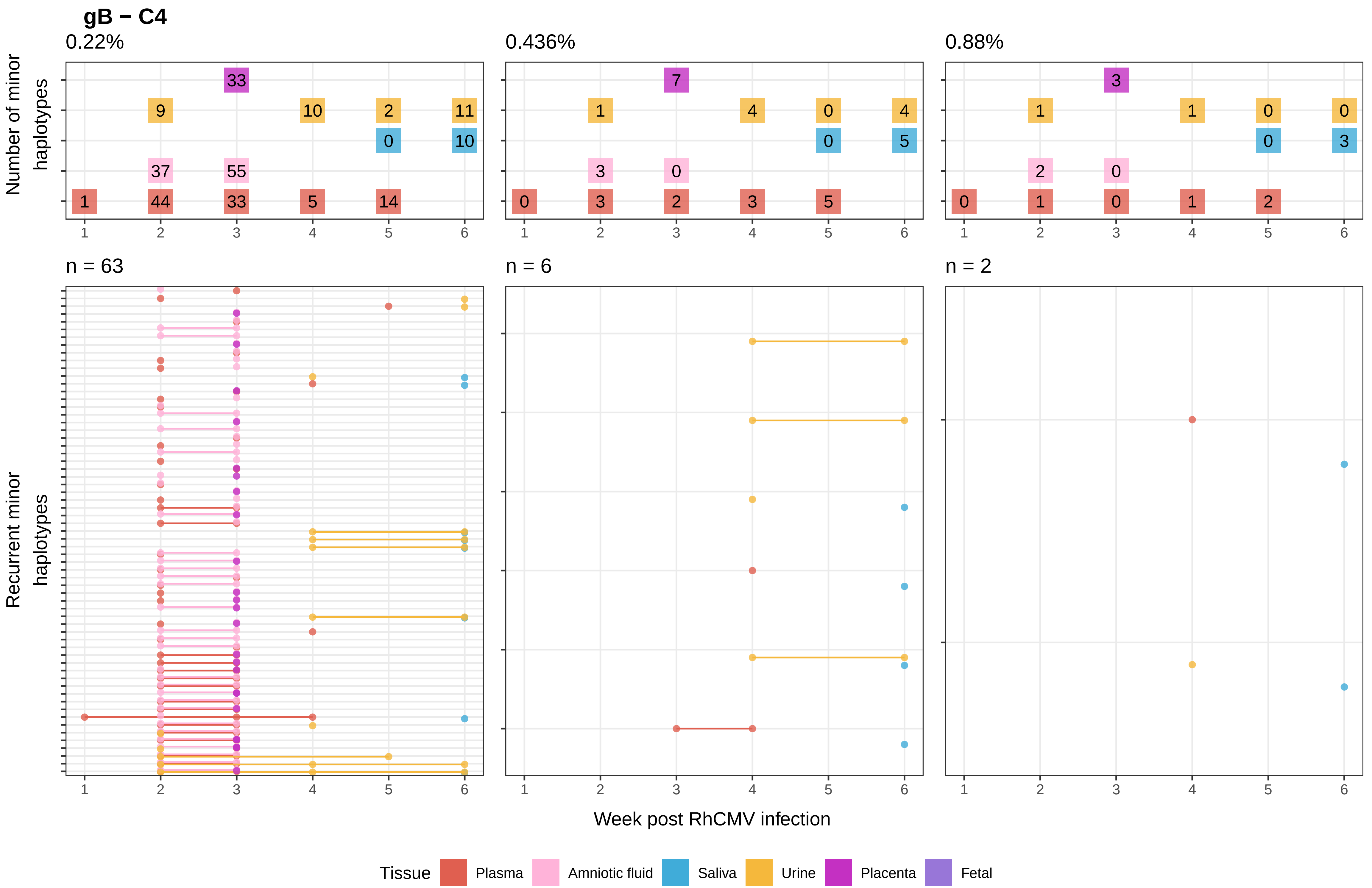

gB − C4
0.22%
0.436%
0.88%
Number of minor haplotypes
33
9
10
2
11
0
10
37
55
1
44
33
5
14
7
1
4
0
4
0
5
3
0
0
3
2
3
5
3
1
1
0
0
0
3
2
0
0
1
0
1
2
n = 63
n = 6
n = 2
Recurrent minor haplotypes
1
2
3
4
5
6
Week post RhCMV infection
Tissue
Plasma
Amniotic fluid
Saliva
Urine
Placenta
Fetal

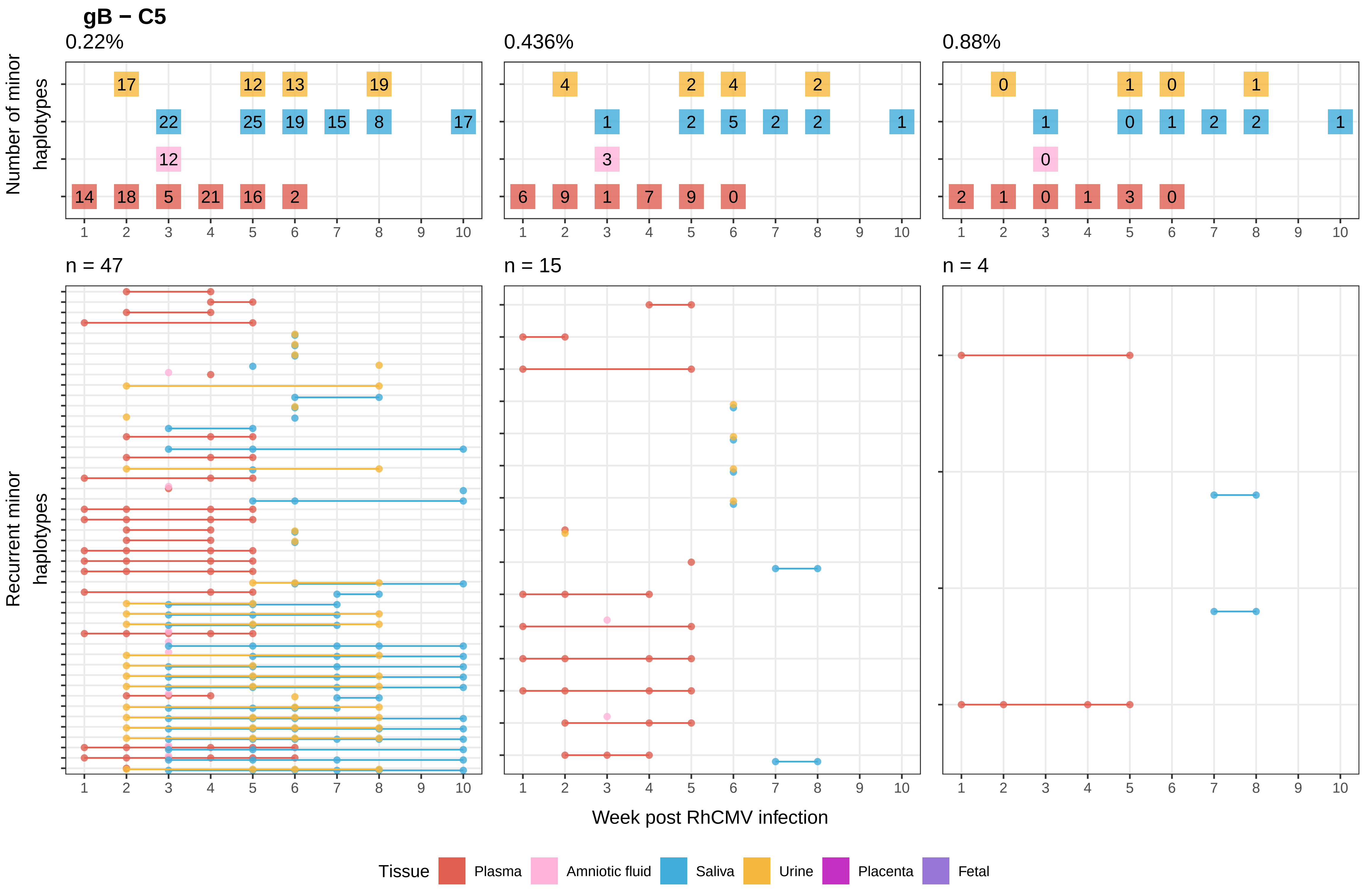
gB − C5
0.22%
0.436%
0.88%
Number of minor haplotypes
n = 47
n = 15
n = 4
Recurrent minor haplotypes
Week post RhCMV infection
Tissue
Plasma
Amniotic fluid
Saliva
Urine
Placenta
Fetal

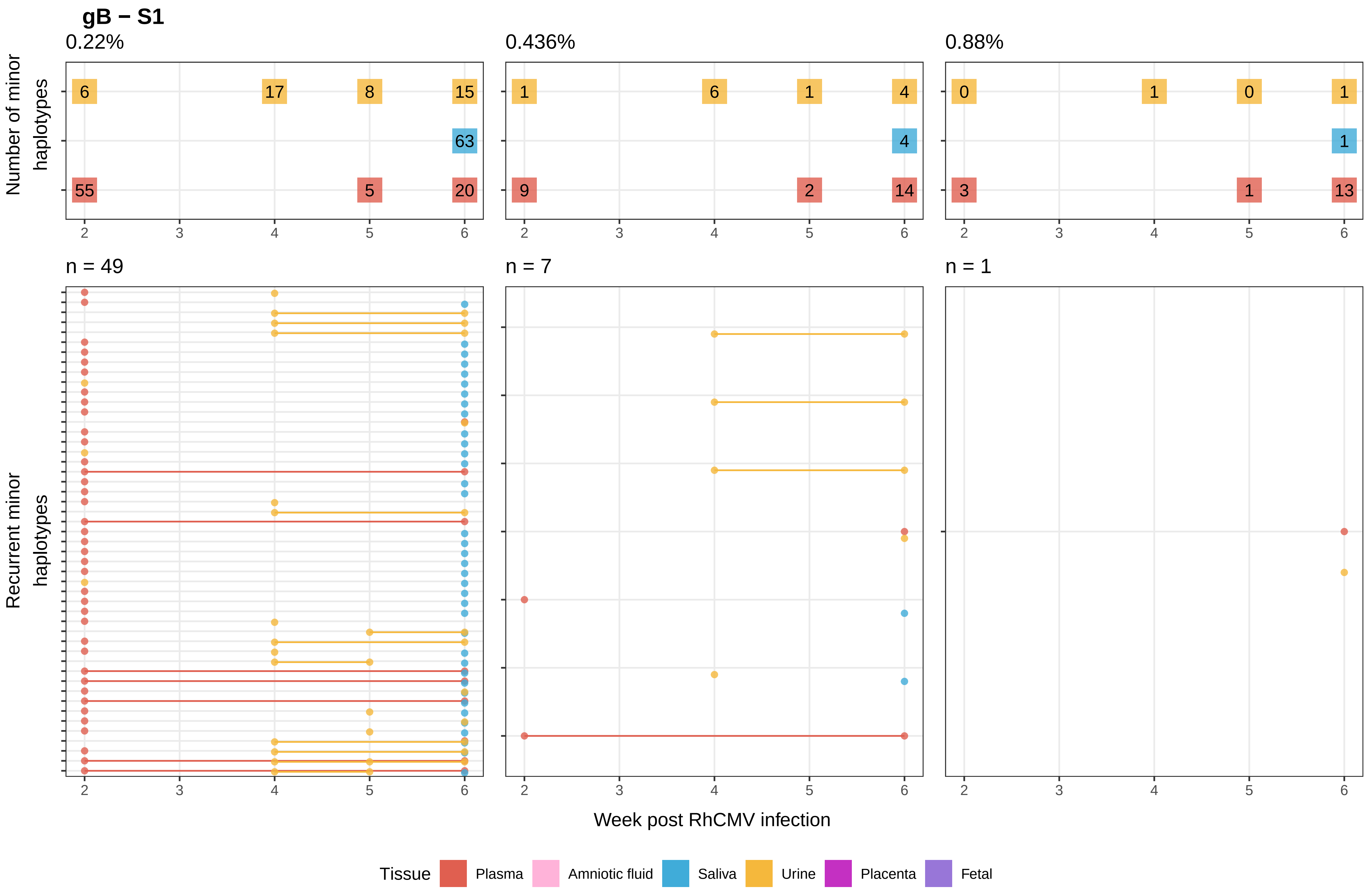
gB − S1
0.22%
0.436%
0.88%
Number of minor haplotypes
6
17
8
15
63
55
5
20
1
6
1
4
4
9
2
14
0
1
0
1
1
3
1
13
n = 49
n = 7
n = 1
Recurrent minor haplotypes
Week post RhCMV infection
Tissue
Plasma
Amniotic fluid
Saliva
Urine
Placenta
Fetal

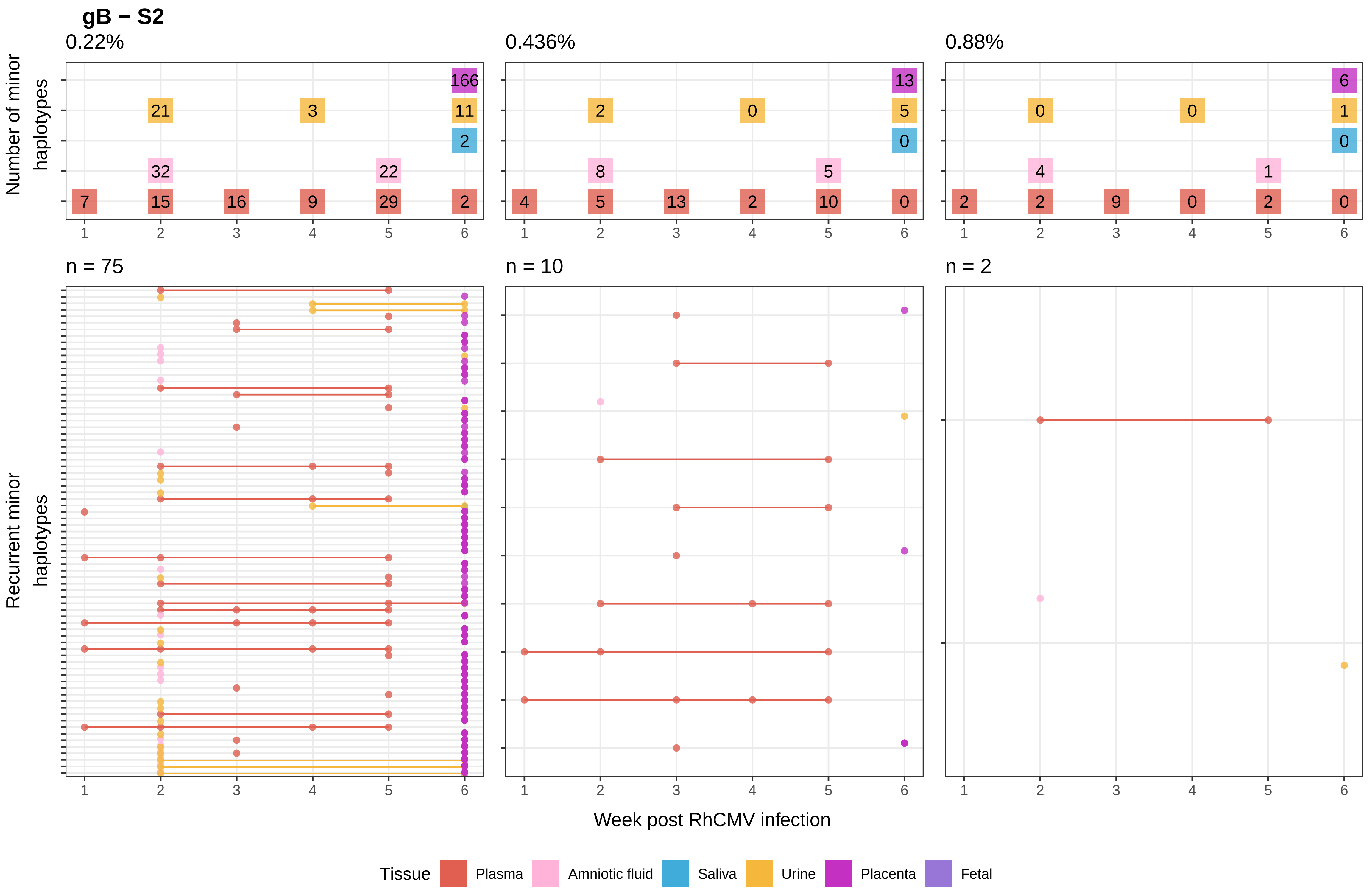

gB − S2
0.22%
0.436%
0.88%
Number of minor haplotypes
n = 75
n = 10
n = 2
Recurrent minor haplotypes
Week post RhCMV infection
Tissue
Plasma
Amniotic fluid
Saliva
Urine
Placenta
Fetal

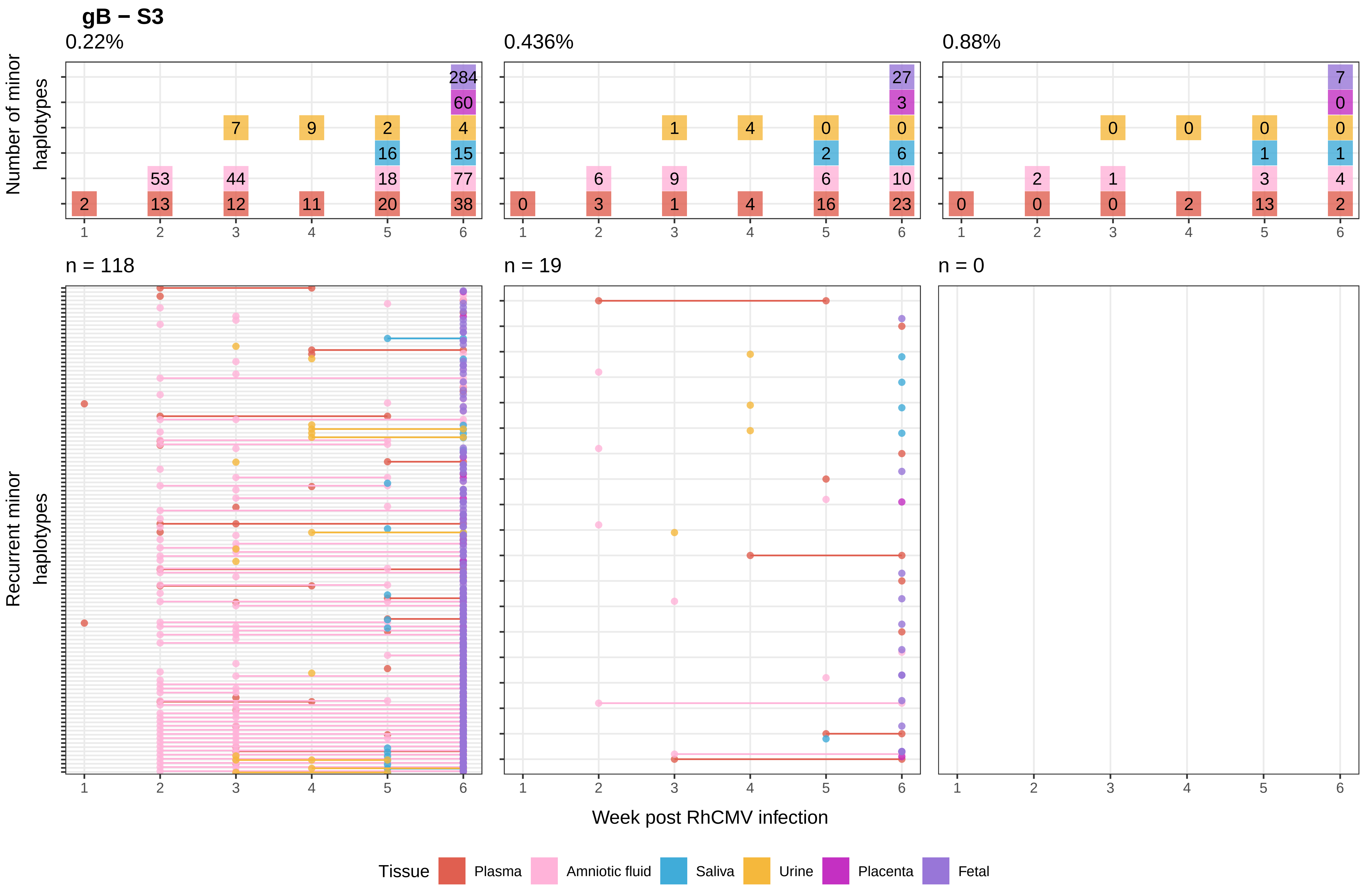

gB − S3
0.22%
0.436%
0.88%
Number of minor haplotypes
284
60
7
9
2
4
16
15
53
44
18
77
2
13
12
11
20
38
27
3
1
4
0
0
2
6
6
9
6
10
0
3
1
4
16
23
7
0
0
0
0
0
1
1
2
1
3
4
0
0
0
2
13
2
n = 118
n = 19
n = 0
Recurrent minor haplotypes
Week post RhCMV infection
Tissue
Plasma
Amniotic fluid
Saliva
Urine
Placenta
Fetal

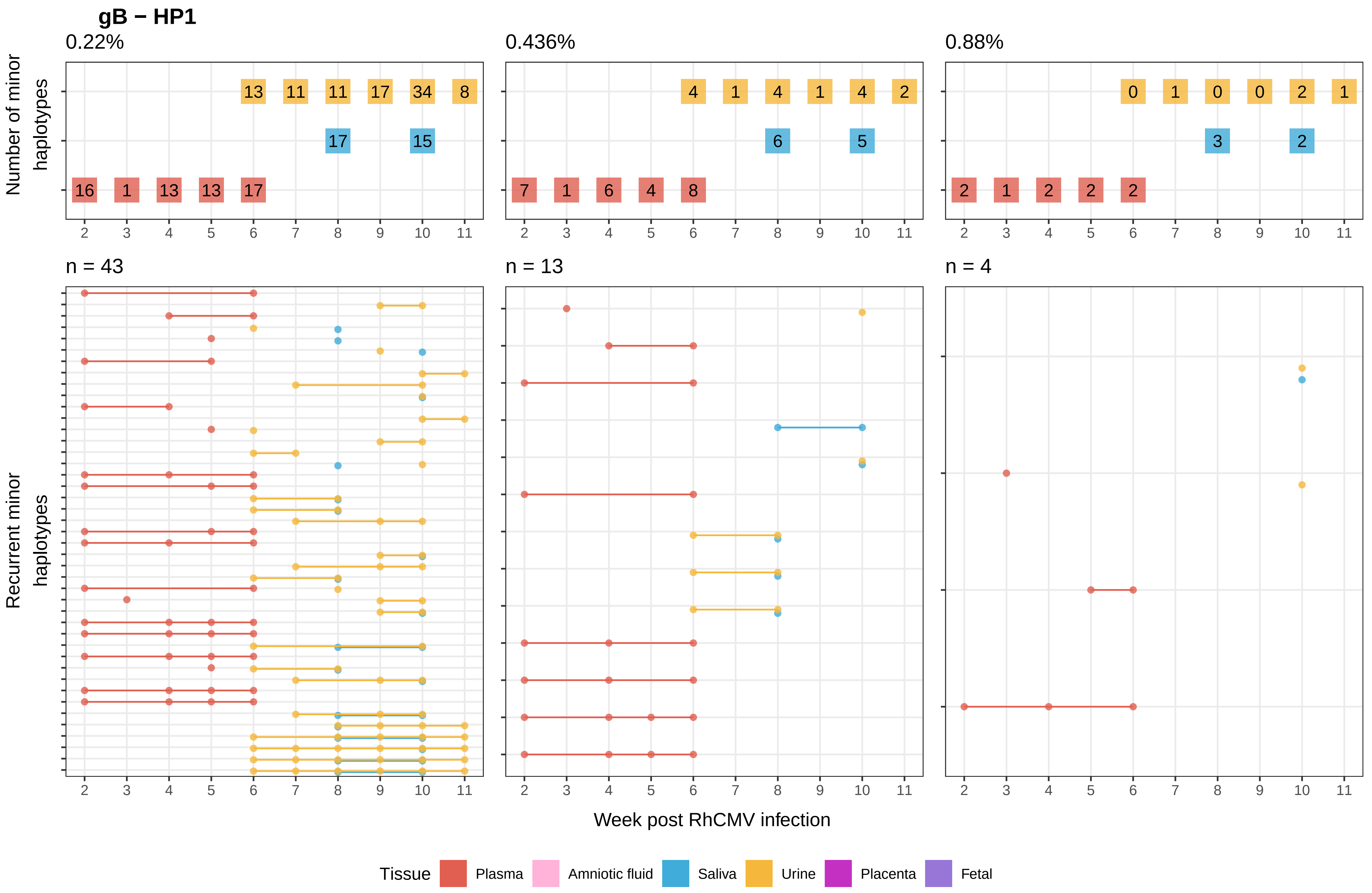
gB − HP1
0.22%
0.436%
0.88%
Number of minor haplotypes
13 11 11 17 34 8
17 15
16 1 13 13 17
4 1 4 1 4 2
6 5
7 1 6 4 8
0 1 0 0 2 1
3 2
2 1 2 2 2
n = 43
n = 13
n = 4
Recurrent minor haplotypes
Week post RhCMV infection
Tissue
Plasma
Amniotic fluid
Saliva
Urine
Placenta
Fetal

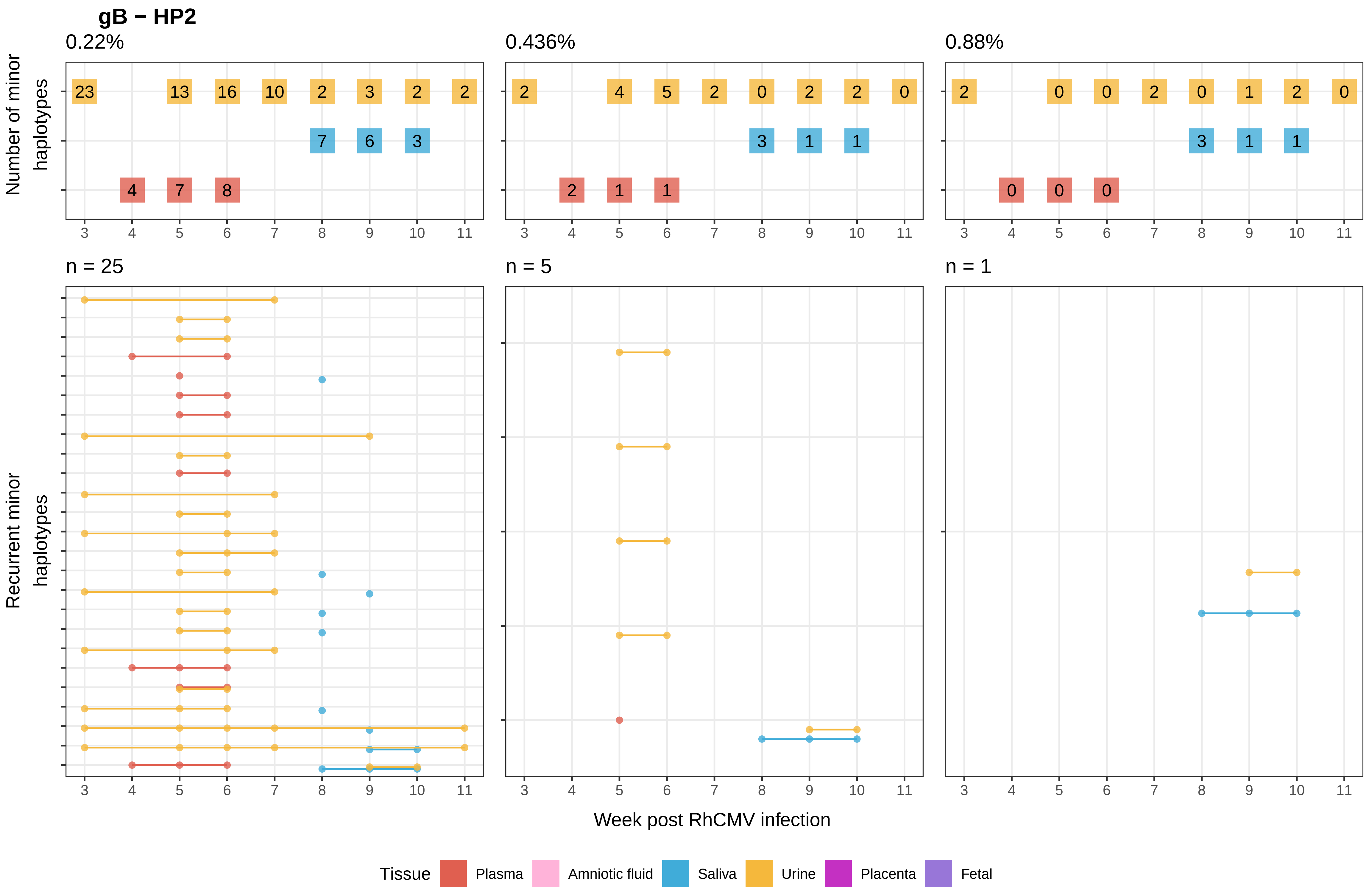
gB − HP2
0.22%
0.436%
0.88%
Number of minor haplotypes
23 13 16 10 2 3 2 2
7 6 3
4 7 8
2 4 5 2 0 2 2 0
3 1 1
2 1 1
2 0 0 2 0 1 2 0
3 1 1
0 0 0
n = 25
n = 5
n = 1
Recurrent minor haplotypes
3 4 5 6 7 8 9 10 11
Week post RhCMV infection
Tissue
Plasma
Amniotic fluid
Saliva
Urine
Placenta
Fetal

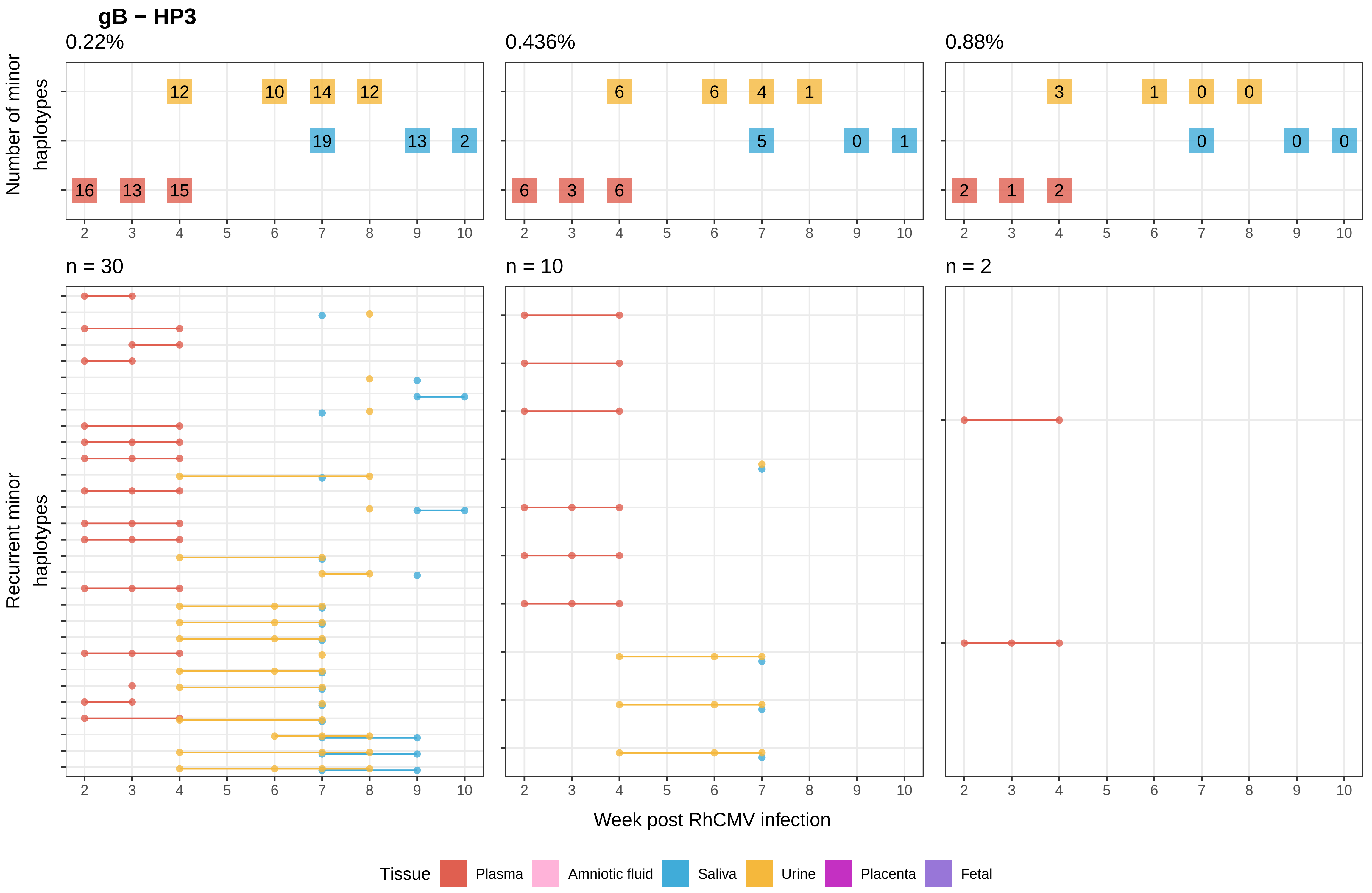

gB − HP3
0.22%
0.436%
0.88%
Number of minor haplotypes
12
10
14
12
19
13
2
16
13
15
6
6
4
1
5
0
1
6
3
6
3
1
0
0
0
0
0
2
1
2
n = 30
n = 10
n = 2
Recurrent minor haplotypes
Week post RhCMV infection
Tissue
Plasma
Amniotic fluid
Saliva
Urine
Placenta
Fetal

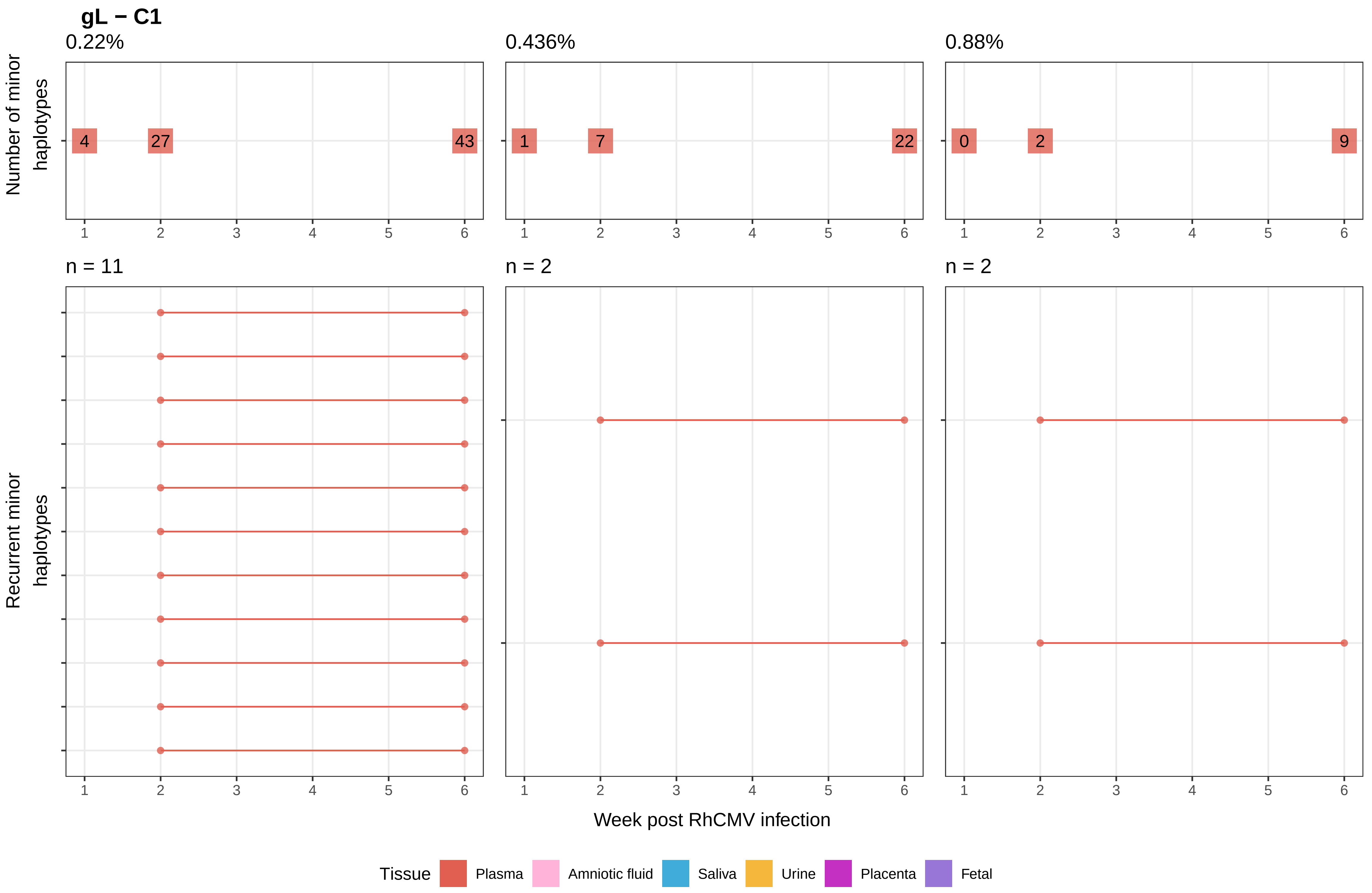
gL − C1
0.22%
0.436%
0.88%
Number of minor haplotypes
4
27
43
1
7
22
0
2
9
n = 11
n = 2
n = 2
Recurrent minor haplotypes
Week post RhCMV infection
Tissue
Plasma
Amniotic fluid
Saliva
Urine
Placenta
Fetal

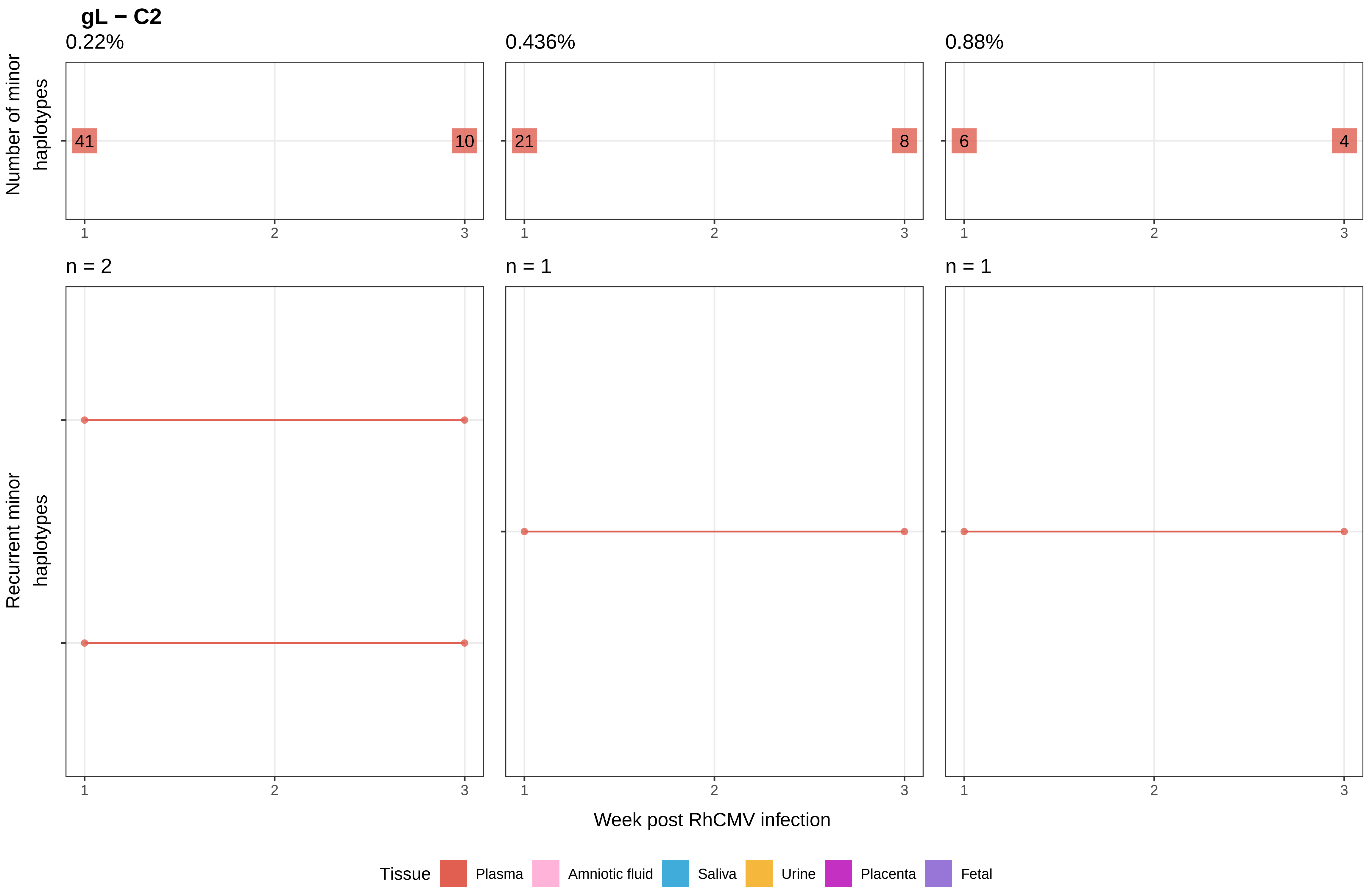
gL − C2
0.22%
0.436%
0.88%
Number of minor haplotypes
41
10
21
8
6
4
n = 2
n = 1
n = 1
Recurrent minor haplotypes
1
2
3
Week post RhCMV infection
Tissue
Plasma
Amniotic fluid
Saliva
Urine
Placenta
Fetal

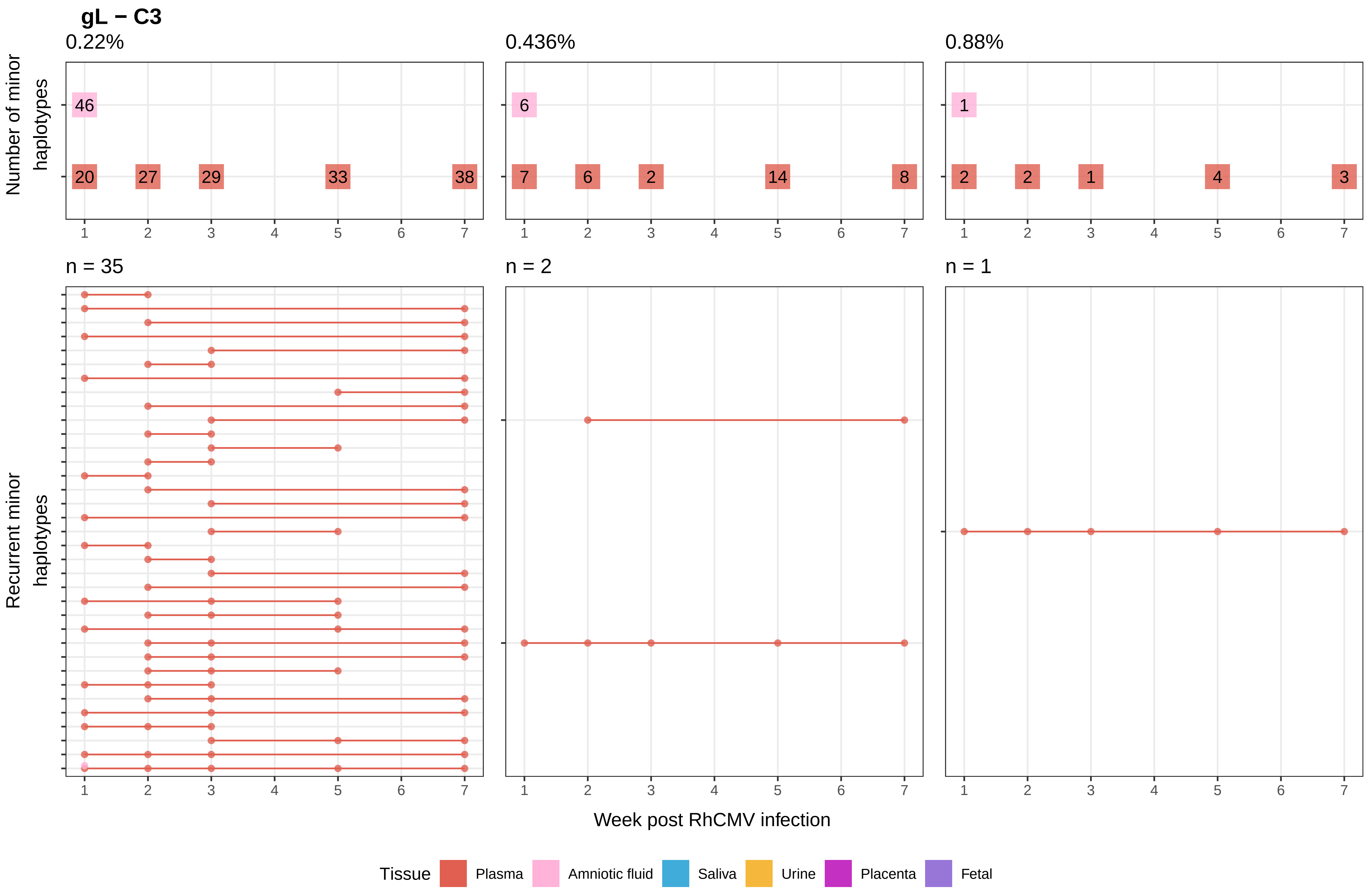

gL − C3
0.22%
0.436%
0.88%
Number of minor haplotypes
46
20
27
29
33
38
6
7
6
2
14
8
1
2
2
1
4
3
n = 35
n = 2
n = 1
Recurrent minor haplotypes
Week post RhCMV infection
Tissue
Plasma
Amniotic fluid
Saliva
Urine
Placenta
Fetal

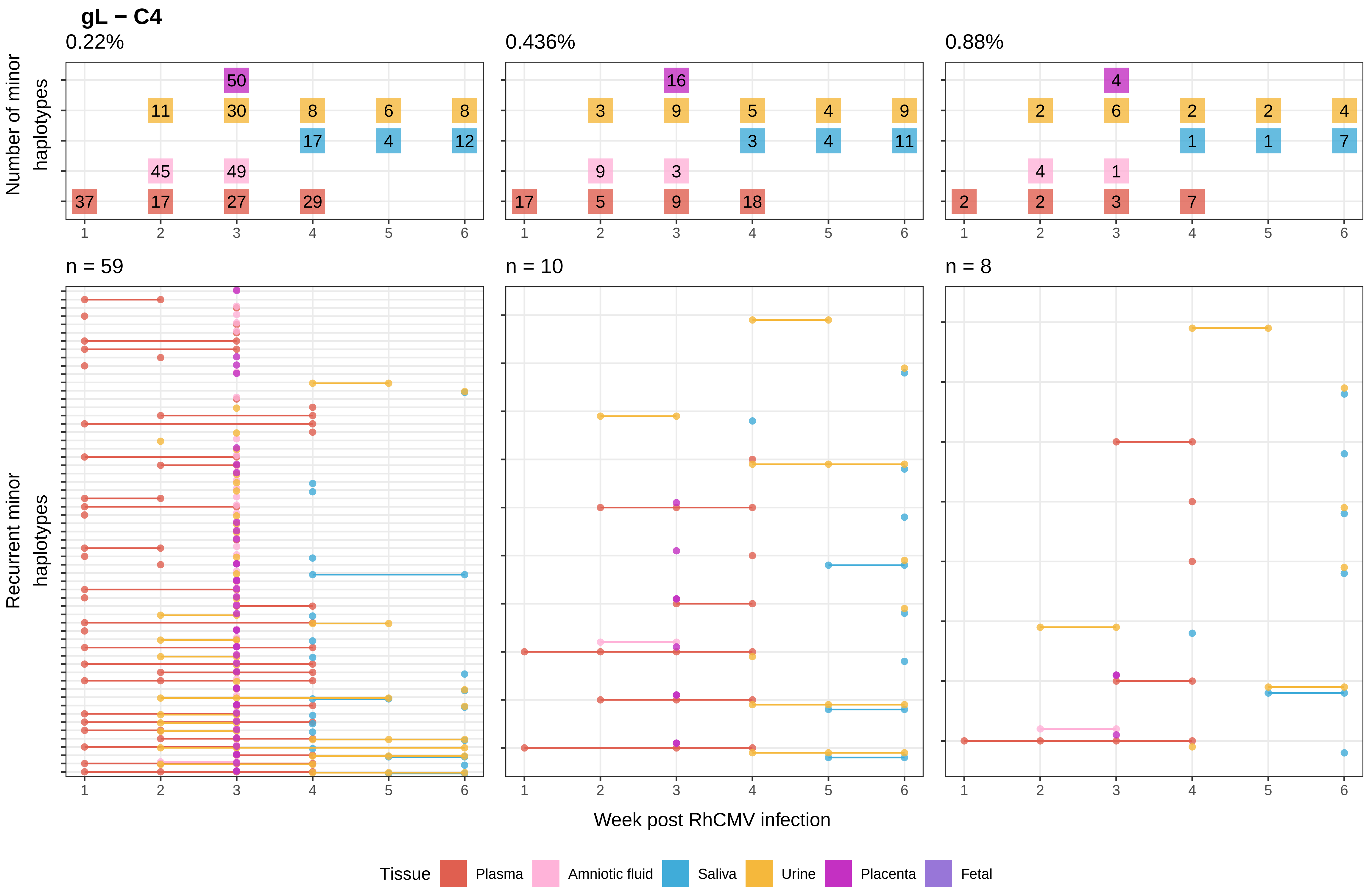

gL − C4
0.22%
0.436%
0.88%
Number of minor haplotypes
50
11
30
8
6
8
17
4
12
45
49
37
17
27
29
16
3
9
5
4
9
3
4
11
9
3
17
5
9
18
4
2
6
2
2
4
1
1
7
4
1
2
2
3
7
n = 59
n = 10
n = 8
Recurrent minor haplotypes
Week post RhCMV infection
Tissue
Plasma
Amniotic fluid
Saliva
Urine
Placenta
Fetal

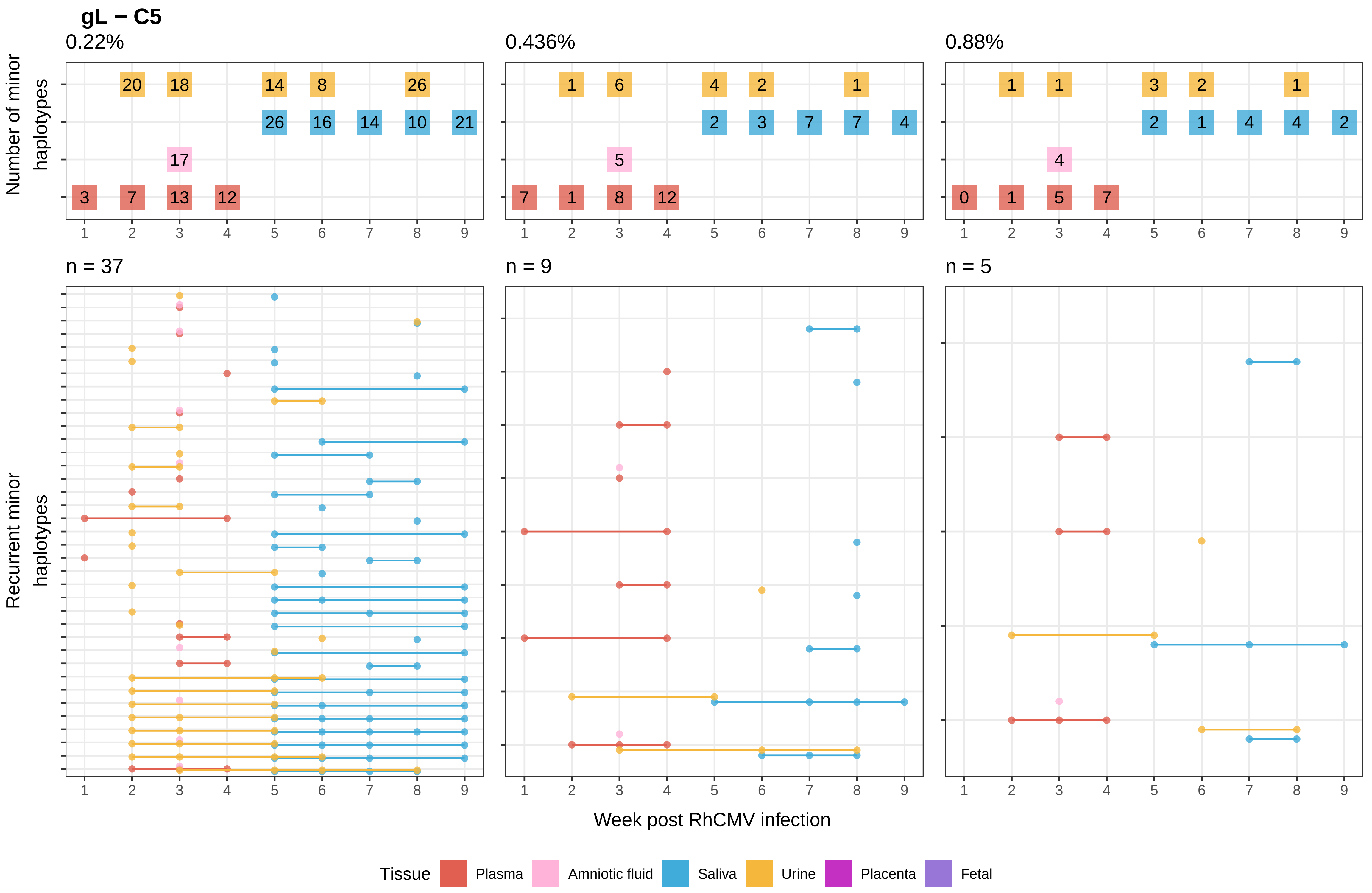

gL − C5
0.22%
0.436%
0.88%
Number of minor haplotypes
20
18
14
8
26
26
16
14
10
21
17
3
7
13
12
1
6
4
2
1
2
3
7
7
4
5
7
1
8
12
1
1
3
2
1
2
1
4
4
2
4
0
1
5
7
n = 37
n = 9
n = 5
Recurrent minor haplotypes
Week post RhCMV infection
Tissue
Plasma
Amniotic fluid
Saliva
Urine
Placenta
Fetal

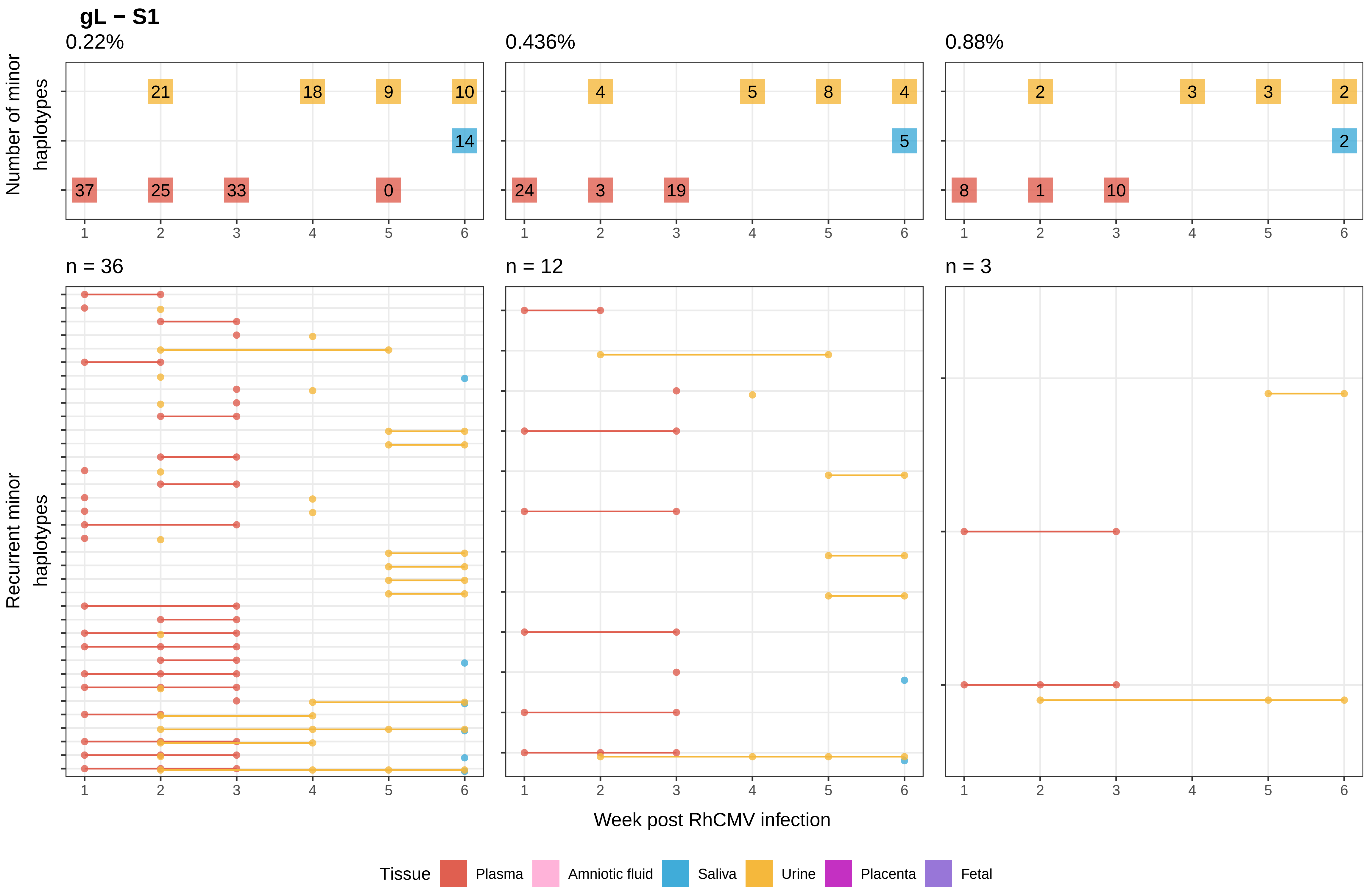

gL − S1
0.22%
0.436%
0.88%
Number of minor haplotypes
21
18
9
10
14
37
25
33
0
4
5
8
4
5
24
3
19
2
3
3
2
2
8
1
10
n = 36
n = 12
n = 3
Recurrent minor haplotypes
Week post RhCMV infection
Tissue
Plasma
Amniotic fluid
Saliva
Urine
Placenta
Fetal

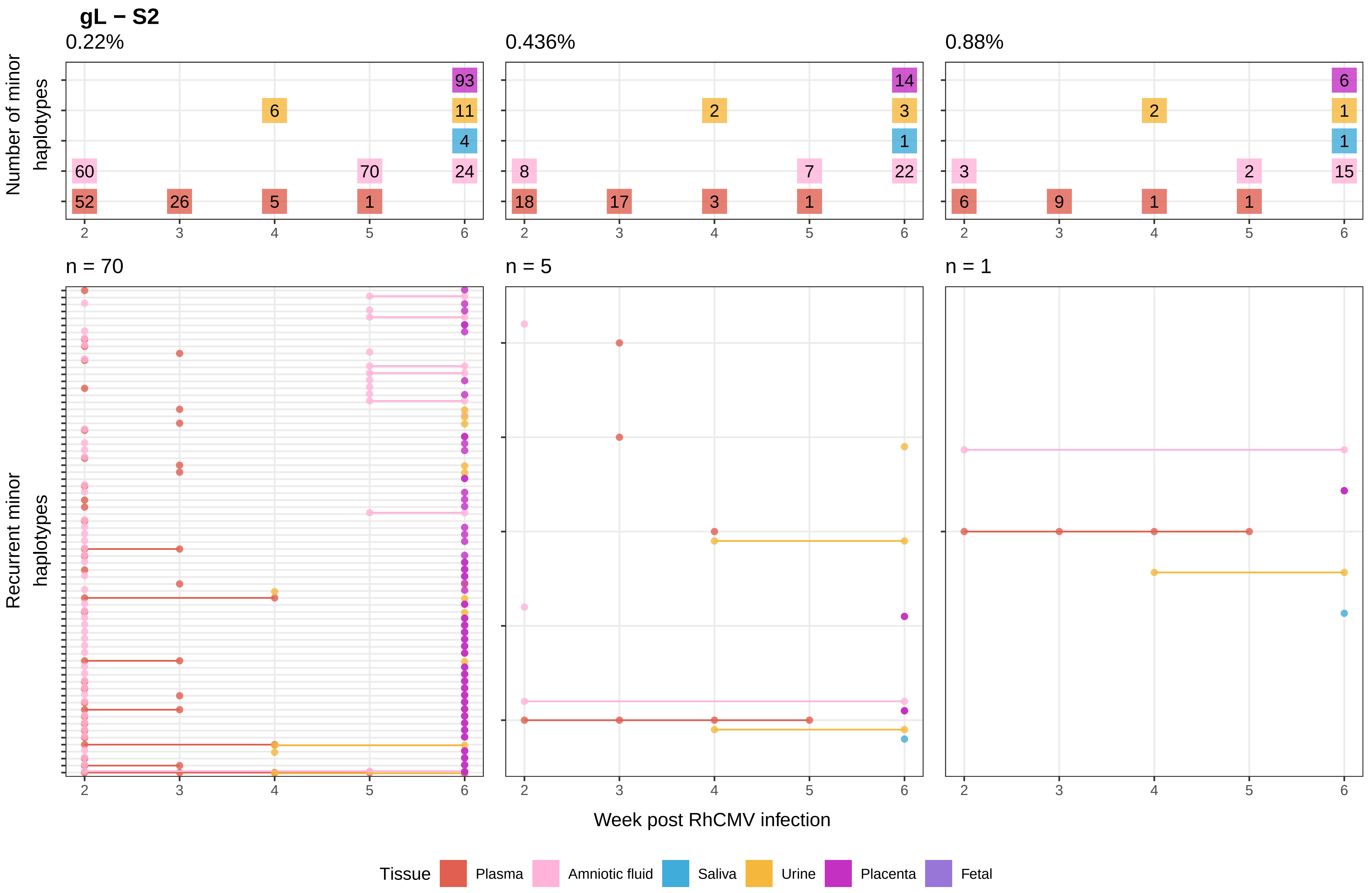

gL − S2
0.22%
0.436%
0.88%
Number of minor haplotypes
93
6
11
4
60
70
24
52
26
5
1
14
2
3
1
8
7
22
18
17
3
1
6
2
1
1
3
2
15
6
9
1
1
n = 70
n = 5
n = 1
Recurrent minor haplotypes
Week post RhCMV infection
Tissue
Plasma
Amniotic fluid
Saliva
Urine
Placenta
Fetal

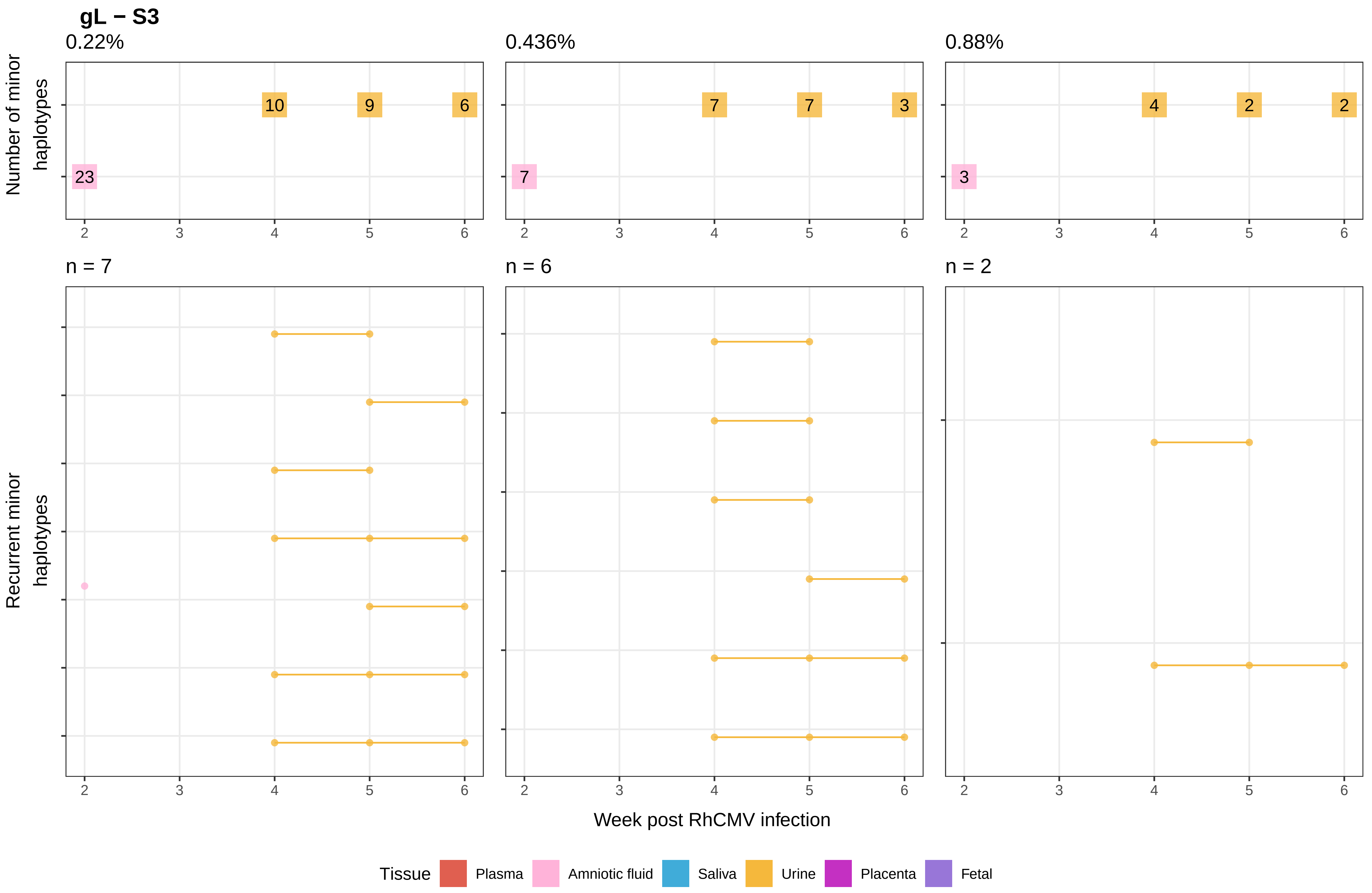
gL − S3
0.22%
0.436%
0.88%
Number of minor haplotypes
10
9
6
23
7
7
3
7
4
2
2
3
n = 7
n = 6
n = 2
Recurrent minor haplotypes
2
3
4
5
6
Week post RhCMV infection
Tissue
Plasma
Amniotic fluid
Saliva
Urine
Placenta
Fetal

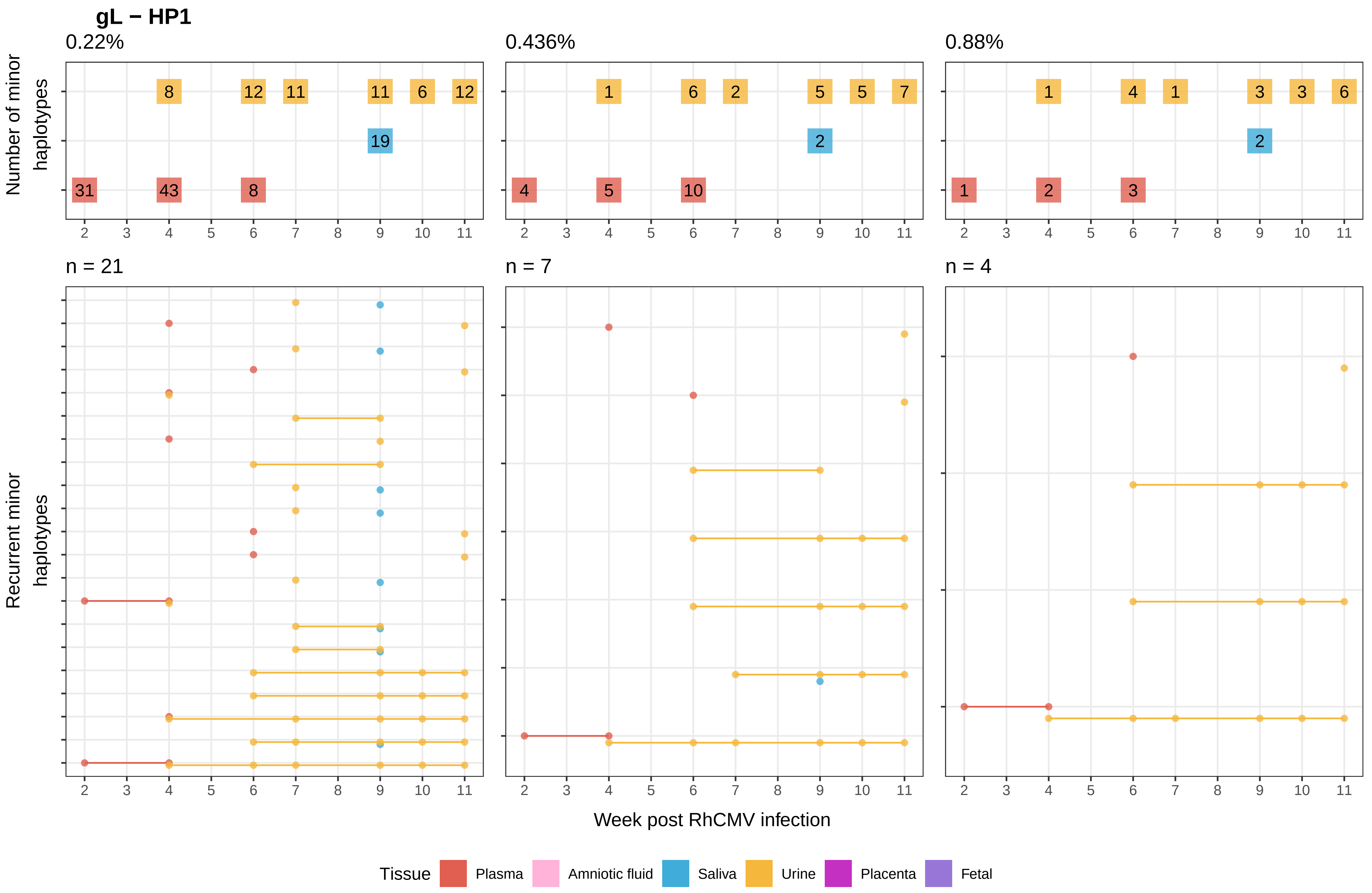
gL − HP1
0.22%
0.436%
0.88%
Number of minor haplotypes
n = 21
n = 7
n = 4
Recurrent minor haplotypes
Week post RhCMV infection
Tissue
Plasma
Amniotic fluid
Saliva
Urine
Placenta
Fetal

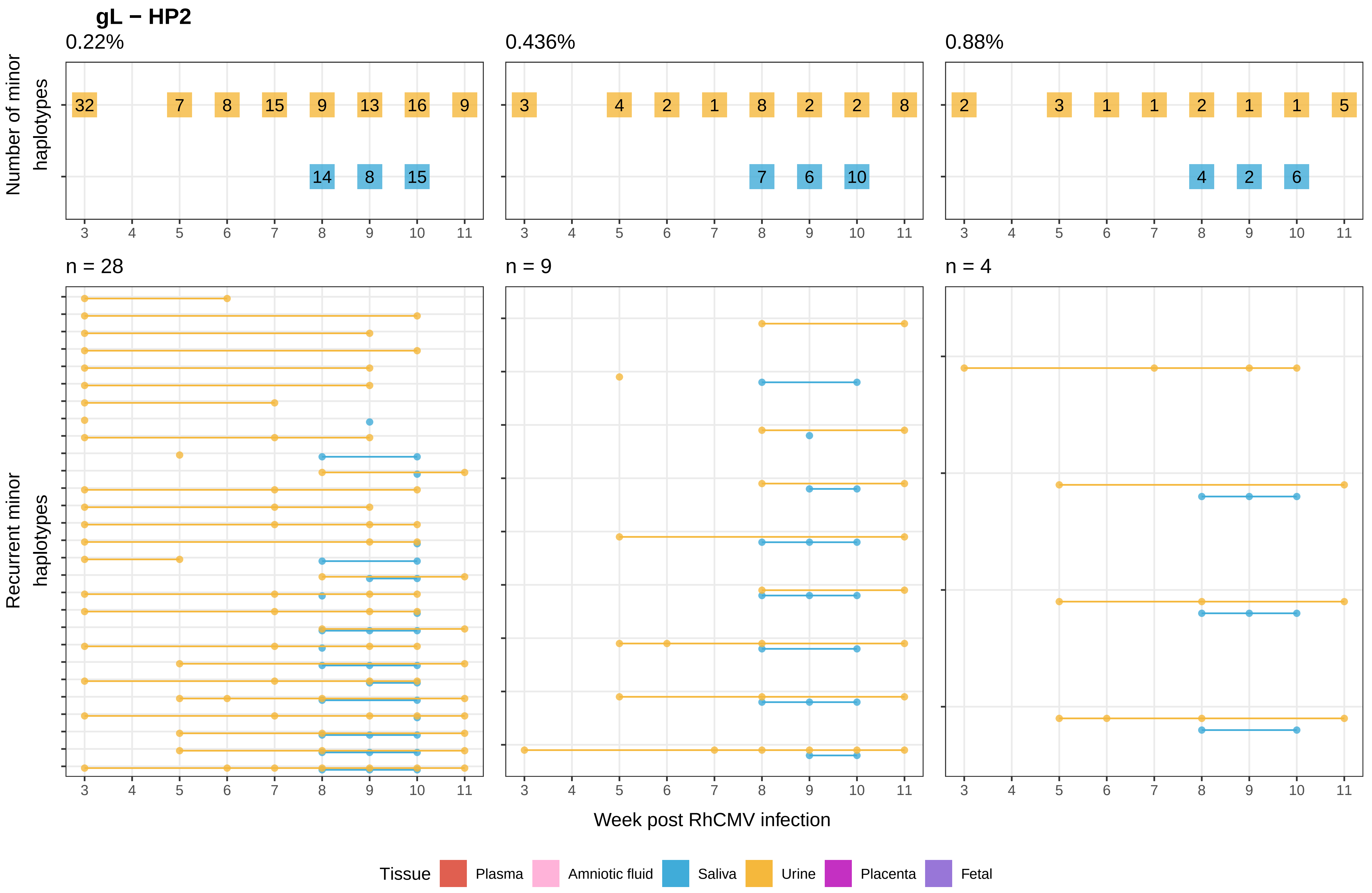
gL − HP2
0.22%
0.436%
0.88%
Number of minor haplotypes
32 7 8 15 9 13 16 9
14 8 15
3 4 2 1 8 2 2 8
7 6 10
2 3 1 1 2 1 1 5
4 2 6
n = 28
n = 9
n = 4
Recurrent minor haplotypes
Week post RhCMV infection
Tissue
Plasma
Amniotic fluid
Saliva
Urine
Placenta
Fetal

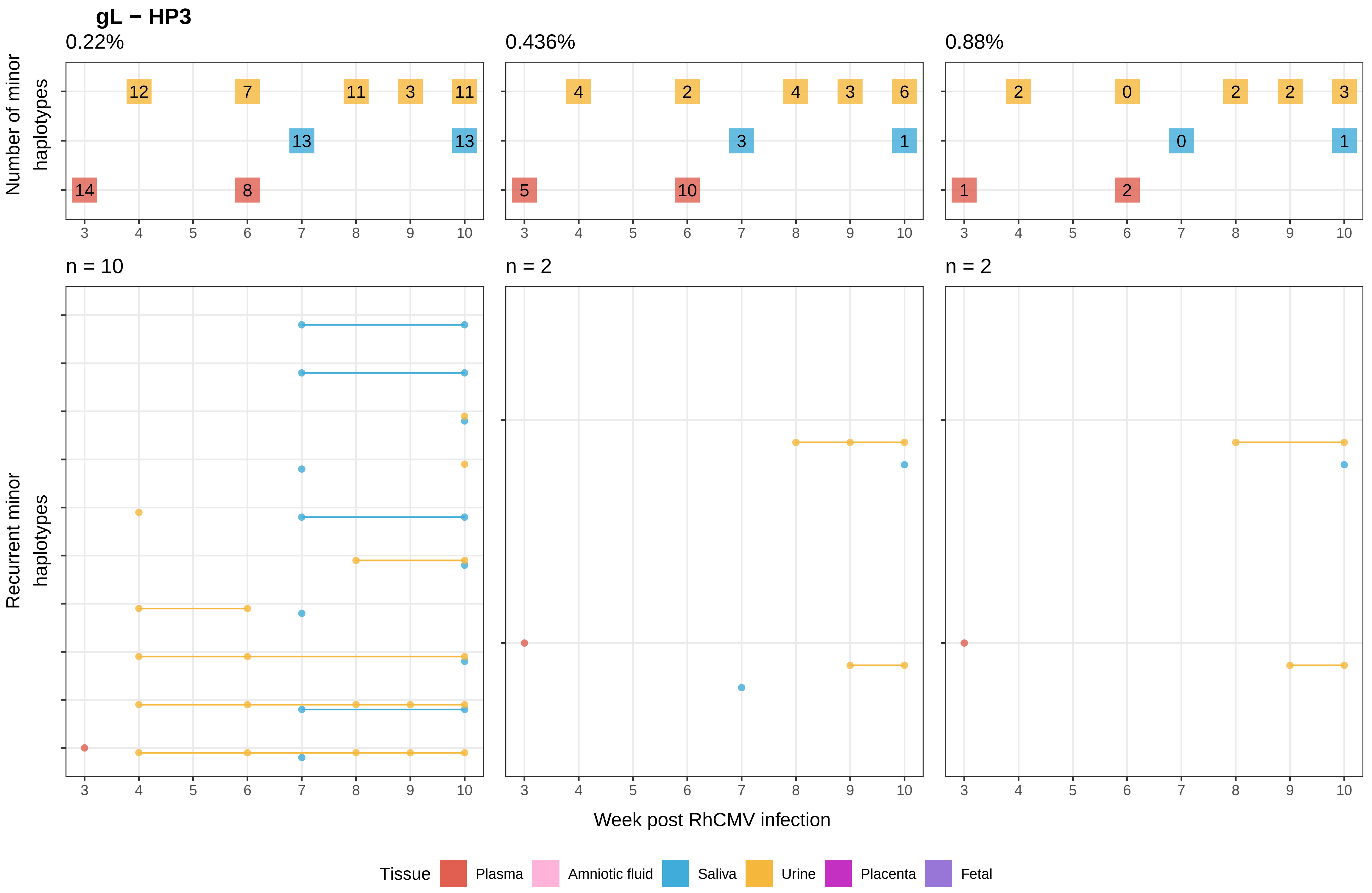

gL − HP3
0.22%
0.436%
0.88%
Number of minor haplotypes
12
7
11
3
11
13
13
14
8
4
2
4
3
6
3
1
5
10
2
0
2
2
3
0
1
1
2
3 4 5 6 7 8 9 10
n = 10
n = 2
n = 2
Recurrent minor haplotypes
Week post RhCMV infection
Tissue
Plasma
Amniotic fluid
Saliva
Urine
Placenta
Fetal
